# Genome-wide identification and multiple abiotic stress transcript profiling of potassium transport gene homologs in *Sorghum bicolor*

**DOI:** 10.3389/fpls.2022.965530

**Published:** 2022-09-02

**Authors:** S. Anil Kumar, P. Hima Kumari, Marka Nagaraju, Palakolanu Sudhakar Reddy, T. Durga Dheeraj, Alexis Mack, Ramesh Katam, P. B. Kavi Kishor

**Affiliations:** ^1^Department of Biotechnology, Vignan’s Foundation for Science, Technology & Research (Deemed to be University), Guntur, India; ^2^Department of Biological Sciences, Florida A&M University, Tallahassee, FL, United States; ^3^Biochemistry Division, National Institute of Nutrition, Hyderabad, India; ^4^International Crops Research Institute for the Semi-Arid Tropics (ICRISAT), Patancheru, India; ^5^Department of Biology, Florida State University, Tallahassee, FL, United States

**Keywords:** HAK/KT/KUP, KEA, K^+^ channels, K^+^ transporters, *Sorghum*, Trk/HKT

## Abstract

Potassium (K^+^) is the most abundant cation that plays a crucial role in various cellular processes in plants. Plants have developed an efficient mechanism for the acquisition of K^+^ when grown in K^+^ deficient or saline soils. A total of 47 K^+^ transport gene homologs (27 HAKs, 4 HKTs, 2 KEAs, 9 AKTs, 2 KATs, 2 TPCs, and 1 VDPC) have been identified in *Sorghum bicolor*. Of 47 homologs, 33 were identified as K^+^ transporters and the remaining 14 as K^+^ channels. Chromosome 2 has been found as the hotspot of K^+^ transporters with 9 genes. Phylogenetic analysis revealed the conservation of sorghum K^+^ transport genes akin to *Oryza sativa*. Analysis of regulatory elements indicates the key roles that K^+^ transport genes play under different biotic and abiotic stress conditions. Digital expression data of different developmental stages disclosed that expressions were higher in milk, flowering, and tillering stages. Expression levels of the genes *SbHAK27* and *SbKEA2* were higher during milk, *SbHAK17*, *SbHAK11*, *SbHAK18*, and *SbHAK7* during flowering, *SbHAK18*, *SbHAK10*, and 23 other gene expressions were elevated during tillering inferring the important role that K^+^ transport genes play during plant growth and development. Differential transcript expression was observed in different tissues like root, stem, and leaf under abiotic stresses such as salt, drought, heat, and cold stresses. Collectively, the in-depth genome-wide analysis and differential transcript profiling of K^+^ transport genes elucidate their role in ion homeostasis and stress tolerance mechanisms.

## Introduction

Potassium (K^+^) is an essential macronutrient and most ubiquitous monovalent cation in plants. It contributes up to 10% of total plant dry weight and plays an overriding role in diverse cellular processes such as ion homeostasis, plant growth, development, transport, and signaling (Feng et al., 2019; Hussain et al., 2021). Despite its abundance, K^+^ is not readily available to plants since they absorb it in the ionic form only, and the concentrations at the root surface often fall below or up to μM range (Ashley et al., 2006). K^+^ uptake in plants is mediated by two mechanisms: a low affinity system that functions when extracellular K^+^ concentration is high (> 200 μM to mM) and a high affinity system that functions when extracellular concentration is low (20 μM Rb^+^) (Gierth et al., 2005). Na^+^ competes with K^+^ but does not fulfill the physiological functions, and higher K^+^/Na^+^ is critical in maintaining electro-neutrality of the cells (Hussain et al., 2021). K^+^ transport occurs through five major families, classified under 2 categories as K^+^ transporters and channels. The transporters include HAK (high-affinity K^+^)/KUP (K^+^ uptake)/KT (K^+^ transporter) family, Trk/HKT family, and KEA (K^+^ efflux anti-porter) family, while the K^+^ channels include the shakers/voltage-gated channels (AKT and KAT) and non-voltage-gated [tandem-pore K^+^ (TPK) and two-pore (TPC)] channels (Hedrich, 2012). HAK/KUP/KT transporters are critical in maintaining osmotic potential and salt tolerance (Feng et al., 2019) and are the largest family of K^+^ transporters (Ahn et al., 2004). While HKTs are involved in the uptake of K^+^ during short-term K^+^ starvation (Riedelsberger et al., 2021), KEAs are implicated in the regulation of thylakoid and stromal pH (Sánchez-McSweeney et al., 2021). Abiotic stresses like salt, drought, heat, and cold impair the final yields (Teklić et al., 2021). But K^+^ is a vital regulator of plant responses and imparts tolerance to the abiotic stresses (Hasanuzzaman et al., 2018; Sardans and Peñuelas, 2021). K^+^ reduces the adverse effects of drought stress tolerance alongside maintaining turgor pressure at low water potentials, alleviates salt stress by achieving homeostatic balance, enhances seed yield during heat stress by reducing the silique canopy, improves freezing tolerance by accumulation of osmolytes (Assaha et al., 2017; Aksu and Altay, 2020; Hu et al., 2021; Saadati et al., 2021).

Genome-wide analyses have been widely performed in HAK/KUP/KT family, but studies were limited to K^+^ transporters. Analysis of HAK/KUP/KT transporters on a genome scale has been carried out in *Oryza sativa* (Banuelos et al., 2002), *Arabidopsis thaliana* (Ahn et al., 2004), *Populus trichocarpa* (He et al., 2012), *Zea mays* (Zhang et al., 2012), *Solanum lycopersicum* (Hyun et al., 2014), *Prunus persica* (Song et al., 2015), *Triticum aestivum* (Cheng et al., 2018), *Pyrus betulifolia* (Li Y. et al., 2018), *Manihot esculenta* (Ou et al., 2018), *Nicotiana tabacum* (Song et al., 2019), *Saccharum spontaneum* (Feng et al., 2020b), *Gossypium* (Yang X. et al., 2020), and *Ipomoea batatas* (Jin et al., 2021). Genome-wide analysis of K^+^ transport gene family has been reported in *Oryza sativa* (Amrutha et al., 2007), *Glycine max* (Rehman et al., 2017), *Cicer arietinum* (Azeem et al., 2018), *Cajanus cajan* (Siddique et al., 2021), and *Gossypium raimondii* (Azeem et al., 2022) but not in *Sorghum bicolor*.

Sorghum, a moderately drought stress-tolerant crop is the fifth most important cereal. It is the staple food for human populations in arid regions and a good source of feed and fuel in the global agronomics and economics. It is a self-pollinated, C_4_ photosynthetic plant with a smaller genome size of 730 Mb (Paterson et al., 2009). In the present study, the discovery, and identification of K^+^ transport gene homologs in *Sorghum bicolor* were conducted including their expression profiles in different tissues under various abiotic stresses. Further, chromosomal locations, gene characterization, protein modeling, conserved motifs analysis, cellular localization, promoter analysis, evolutionary relationship, and protein-protein interactions were investigated, resulting in the characterization of candidate genes.

## Materials and methods

### *In silico* prediction, identification, and characterization of K^+^ transport gene homologs

All the 49 rice full-length cDNA sequences of K^+^ encoding genes were collected from rice (Amrutha et al., 2007). The homology search of the collected FASTA sequences was performed by BLASTN against Sorghum genome in the Gramene database with default settings.^1^ The coding sequences (CDS) and corresponding protein sequences were retrieved from the BLAST output using GENSCAN web server.^2^ To check the presence of the K^+^ domain, Conserved Domain Database search and SMART were employed.^3^, ^4^ For analysis of transmembrane, TMHMM (Moller et al., 2001), for prediction of gene structure, Gene Structure Display Server,^5^ for prediction of subcellular localization of the protein, WoLFPSORT,^6^ for isoelectric point (pI) molecular weight (MW), GRAVY (grand average of hydropathy) instability, and aliphatic indexes, ProtParam software,^7^ for identification of phosphorylation sites, NetPhos 3.1^8^ for amino acid composition and net charge, pepcalc^9^ for conserved motifs, MEME^10^ with parameters like 10 number of motifs, 2–20 motif sites, 6–20 wide motif width were used. The genes on the chromosomes were mapped based on their physical location using MG2C^11^ tool.

### Prediction of *cis*-elements, protein modeling and protein-protein interactions

Promoter elements were identified for all the transporter and channel genes by taking 2 kb sequence upstream to all the sorghum K^+^ transport homologs using PLACE tools.^12^ The 3D structures of all the K^+^ transport proteins were predicted using SWISS-MODEL server (Biasini et al., 2014). The predicted 3D structures of proteins were evaluated for stability using protein structure verification server (PSVS).^13^ The stability of the proteins was analyzed by Ramachandran plots. The predicted protein-protein interaction (PPI) map of sorghum K^+^ transport homologs was generated from the STRING database.^14^

### Phylogenetic analysis and generation of synteny maps

A phylogenetic tree was constructed with amino acid sequences of *Sorghum bicolor* (Sb), *Oryza sativa* (Os), and *Arabidopsis thaliana* (At) using MEGA 10.0 software, by Neighbor-Joining method with 1000 bootstrap replicates (Kumar et al., 2018). Evolutionary analysis of orthologs and paralogs was performed by calculating synonymous (dS) and non-synonymous (dN) substitution rates using the PAL2NAL program.^15^ Synonymous (dS) and non-synonymous (dN) substitution rates were calculated by codeml in the PAML package. Synteny and collinearity were analyzed to identify K^+^ homologs using TBtools (Chen et al., 2020).

### Digital and qRT-PCR analysis of K^+^ transport gene homologs under different abiotic stresses

For digital expression profiling of K^+^ transport genes, Genevestigator^16^ was used. The mRNA-seq data were used for analysis. The data are available for all the 46 genes (except SbHAK26) for 2 stress conditions (cold and drought) in 3 tissues (root, shoot, and leaf), and 4 developmental stages (milk stage, seedling stage, tillering stage, and flowering stage). Using hierarchical clustering, heat maps were generated separately for anatomical, developmental, and perturbations. Seeds of *Sorghum bicolor* L. BTx623 genotype were collected from the International Crops Research Institute for the Semi-Arid Tropics and used. Seventy-five-day-old seedlings maintained in green house at 28/20^°^C day/night temperatures were treated with salt (200 mM NaCl), drought (200 mM mannitol), heat (42^°^C), and cold (4^°^C) stresses for 4 h. Control (without any stress) plants were treated with tap water. Root, stem, and leaftissue samples were collected immediately, snap frozen in liquid nitrogen and stored at −80°C. Total RNA was isolated from all the samples using Macherey-Nagel NucleoSpin RNA plant kit (740949.50) by following the instructions manual. Total RNA (2 μg) was taken as a template for first strand cDNA synthesis using RevertAid First Strand cDNA Synthesis Kit (#K1622, Thermo Scientific EU, Reinach, Switzerland). The relative expression levels of K^+^ gene homologs were studied using Mx3000p (Agilent) with 2X applied biosystems (ABI) Master Mix with gene specific primers (Supplementary Table 1) with the following thermal cycles: 1 cycle at 95^°^C for 10 min, followed by 40 cycles alternatively at 95^°^C for 15 s and 60^°^C for 1 min. The expression of each gene in various samples was normalized with *ACTIN* gene. The experiment was performed with two biological replicates and for each sample three technical replicates were used. The comparative 2*^–^*^ΔΔ*CT*^ method was used to calculate the relative quantities of each transcript in the samples (Schmittgen and Livak, 2008).

## Results

### Discovery, identification and characterization of K^+^ gene homologs

**FIGURE 1:**
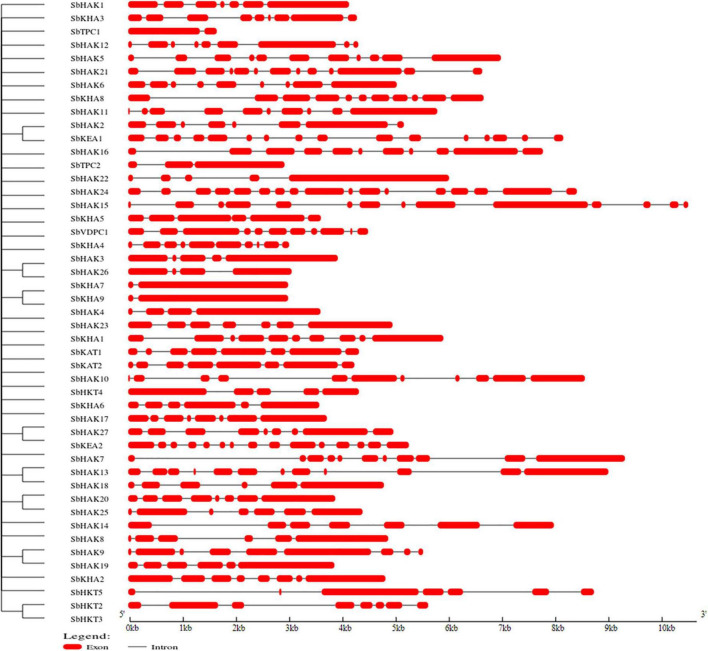
Characterization of K^+^ transporter gene homologs. Exons are represented as red boxes and introns as black lines. Sb, *Sorghum bicolor*; HAK, high affinity potassium; KEA, K^+^ efflux antiporter; VDPC, voltage-dependent potassium channel; TPC, two-pore channels.

**FIGURE 2:**
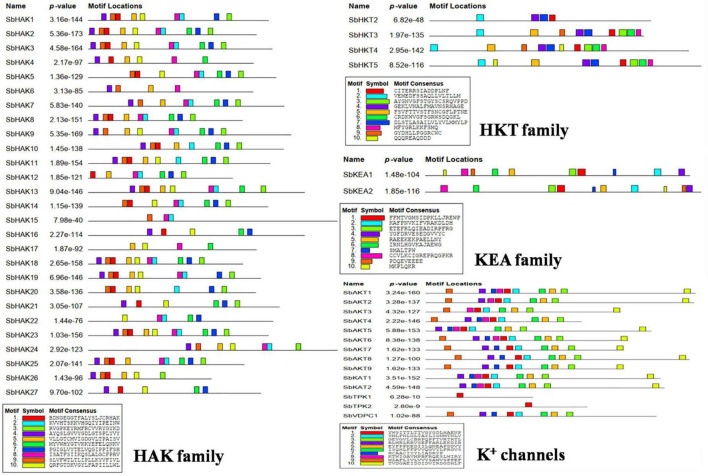
Conserved motif analysis of K^+^ transporter (HAK, HKT, and KEA) and channel (AKT, KAT, VDPC, and TPC) proteins. The consensus motif GVVYGDLGTSPLY was identified in all the HAK transporters except HAK5, HAK12, and HAK22. The motifs, VEMEDFSSAQLLVLTLLM, FSVFTTVSTFSNCGFLPTNE, GEKLVNALFMAVNSRHAGE, and LSTLASAILVLYVLMMYLP were observed in all the sorghum HKT transport proteins. The KEA family displayed FFMTVGMSIDPKLLJREWP and KAFPNVKIFVRAKDLDH motifs. All the channel proteins displayed the motif YWSITTLTTVGYGDLHAENP. Sb, *Sorghum bicolor*; HAK, high affinity potassium; KEA, K^+^ efflux antiporter; VDPC, voltage-dependent potassium channel; TPC, two-pore channels.

A total of 47 K^+^ transport homologs have been identified in sorghum (Table 1 and Supplementary Table 2). Of the 47 transport homologs 27 belong to HAKs (*SbHAK1* to *SbHAK27*), 4 HKTs (*SbHKT2*, *SbHAT3*, *SbHKT4*, and *SbHKT5*), 9 AKTs (*SbAKT1* to *SbAKT9*), 2 KEAs (*SbKEA1* and *SbKEA2*), 2 KATs (*SbKAT1* and *SbKAT2*), 2 TPKs (*SbTPC1* and *SbTPC2*), and 1 VDPC (*SbVDPC1*) (Table 1). Homologs of *OsHKTs* (*OsHKT1*, *OsHKT6*, and *OsHKT7*) and *OsKEA3* are not available in *Sorghum bicolor*. K^+^ transporter and channel domains like K-trans, TrK, voltage-dependent K^+^ channel, KHA, Two pore potassium channel, and K^+^-efflux system protein have been identified (Table 1). Predicted amino acid sequences were used to identify the number of transmembrane segments. While the number of transmembrane domains for K^+^ transporters varies from 0 (*SbAKT2*) to 13 (*SbHAK11* and *SbHAK17*) (Table 1), the number of exons varies from 2 (*SbAKT7*, *SbAKT9*, and *SbTPC1*) to 17 (*SbHAK24*) in sorghum (Table 1 and Figure 1). Most of the K^+^ transport gene homologs are localized on the plasma membrane, followed by chloroplast, nucleus, endoplasmic reticulum, and mitochondria. SbHAK6 and HAK15 are localized on nucleus, SbAKT1, SbAKT2, and SbKEA on chloroplast, SbAKT5 and SbKAT2 on endoplasmic reticulum, and SbKAT1 on mitochondria (Table 1). The pI, MW, GRAVY, instability, aliphatic indexes (Supplementary Table 3), amino acid and net charge (Supplementary Table 4) have also been tabulated. Kinases play an important role in phosphorylation and K^+^ gene homologs showed 18 different serine, threonine, and tyrosine kinases (Supplementary Table 5). The consensus motif GVVYGDLGTSPLY was identified in all the HAK transporter proteins except HAK5, HAK12, and HAK22 (Figure 2). Another signature sequence like GGTFALYSLLCR has been observed in all the HAK transporters leaving out HAK7, HAK15, and HAK22. The motif SLVFWTLTLIPLLKYVFIVL has been detected in all the HAK transporters excluding HAK12 (Figure 2). The motifs, VEMEDFSSAQLLVLTLLM, FSVFTTVSTFSNCGFLPTNE, GEKLVNALFMAVNSRHAGE, and DLSTLASAILVLYVLMMYLP were noticed in all the sorghum HKT K^+^ transporter proteins (Figure 2). The KEA family displayed FFMTVGMSIDPKLLJREWP and KAFPNVKIFVRAKDLDH motifs (Figure 2). The sorghum K^+^ channel (AKT, KAT, TPK, and VDPC) proteins displayed the motif YWSITTLTTVGYGDLHAENP (Figure 2).

**TABLE 1:** Characterization of *Sorghum bicolor* potassium transport gene homologs.

Gene	Gene id	Cds	Aa	Chr	Domain	TMHMM	Exons	Localization
> SbHAK1	SORBI_3006G061300	2316	771	6	K-trans	12	7	PM
> SbHAK2	SORBI_3003G418100	2163	720	3	K-trans	10	8	PM
> SbHAK3	SORBI_3003G164400	2367	788	3	K-trans	11	5	PM
> SbHAK4	SORBI_3007G153001	2124	707	7	K-trans	9	4	PM
> SbHAK5	SORBI_3003G413700	2412	803	3	K-trans	10	11	PM
> SbHAK6	SORBI_3007G209900	2121	706	7	K-trans	8	9	N
> SbHAK7	SORBI_3002G411500	2517	838	2	K-trans	8	11	PM
> SbHAK8	SORBI_3001G379900	1983	660	1	K-trans	9	6	PM
> SbHAK9	SORBI_3002G417500	2604	867	2	K-trans	11	9	PM
> SbHAK10	SORBI_3010G197500	2511	836	10	K-trans	6	11	PM
> SbHAK11	SORBI_3006G213500	2340	779	6	K-trans	13	10	PM
> SbHAK12	SORBI_3007G075100	1860	619	7	K-trans	8	9	PM
> SbHAK13	SORBI_3010G224400	2784	927	10	K-trans	9	12	PM
> SbHAK14	SORBI_3002G313900	2313	770	2	K-trans	10	7	PM
> SbHAK15	SORBI_3006G210700	3261	1086	6	K-trans	3	14	N
> SbHAK16	SORBI_3001G184000	2781	926	1	K-trans	9	11	PM
> SbHAK17	SORBI_3002G220600	2160	719	2	K-trans	13	8	PM
> SbHAK18	SORBI_3002G130800	1986	661	2	K-trans	10	6	PM
> SbHAK19	SORBI_3004G160000	2217	738	4	K-trans	10	6	PM
> SbHAK20	SORBI_3006G062100	2154	717	6	K-trans	10	8	PM
> SbHAK21	SORBI_3001G183700	2457	818	1	K-trans	11	13	PM
> SbHAK22	SORBI_3002G001800	2379	792	2	K-trans	9	5	PM
> SbHAK23	SORBI_3002G188600	2316	771	2	K-trans	10	7	PM
> SbHAK24	SORBI_3010G112800	3339	1112	10	K-trans	10	17	PM
> SbHAK25	SORBI_3004G250700	2007	668	4	K-trans	7	7	PM
> SbHAK26	Unknown	1587	528	3	K-trans	5	4	PM
> SbHAK27	SORBI_3001G184300	2217	738	1	K-trans	11	9	PM
> SbHKT2	SORBI_3004G059800	1749	582	4	Trk	3	8	PM
> SbHKT3	SORBI_3006G208100	1692	563	6	Trk	7	3	PM
> SbHKT4	SORBI_3003G145800	2046	681	3	Trk	8	5	PM
> SbHKT5	SORBI_3010G251700	2145	714	10	Trk	7	7	PM
> SbAKT1	SORBI_3003G237900	2625	874	3	VDPC	5	10	C
> SbAKT2	SORBI_3002G049700	2607	868	2	VDPC	0	8	C
> SbAKT3	SORBI_3004G107500	1899	632	4	VDPC	3	9	PM
> SbAKT4	SORBI_3003G300600	1518	505	3	VDPC	4	10	PM
> SbAKT5	SORBI_3009G146800	2196	731	9	VDPC	2	6	ER
> SbAKT6	SORBI_3003G278200	1968	655	3	VDPC	5	6	PM
> SbAKT7	SORBI_3004G193100	1926	641	4	KHA	4	2	PM
> SbAKT8	SORBI_3010G102800	2565	854	10	VDPC	5	11	PM
> SbAKT9	SORBI_3006G201000	1926	641	6	KHA	4	2	PM
> SbKAT1	SORBI_3009G147500	2280	759	9	VDPC	3	8	M
> SbKAT2	SORBI_3009G147200	2319	772	9	VDPC	3	8	ER
> SbTPC1	SORBI_3001G086900	1044	347	1	TPC	6	2	PM
> SbTPC2	SORBI_3002G162400	1572	523	2	TPC	5	3	PM
> SbVDPC1	SORBI_3006G093400	2241	746	6	VDPC	4	11	PM
> SbKEA1	SORBI_3006G271800	2046	681	6	KEFC	10	16	PM
> SbKEA2	SORBI_3008G173800	2136	711	8	KEFC	7	15	C

Cds, Coding sequence; Aa, amino acid length; Chr, chromosomal location; TMHMM, transmembrane domain; Sb, Sorghum bicolor; HAK, high affinity potassium; VDPC, voltage-dependent potassium channel; TPC, two-pore channels; KEA, K^+^ efflux antiporter; K-trans, K^+^ transport; KEFC, K^+^-efflux system protein; PM, Plasma membrane; N, Nucleus; ER, Endoplasmic reticulum; C, Chloroplast; M, Mitochondria.

### Promoter, 3D protein structures, and PPI analysis

**FIGURE 3:**
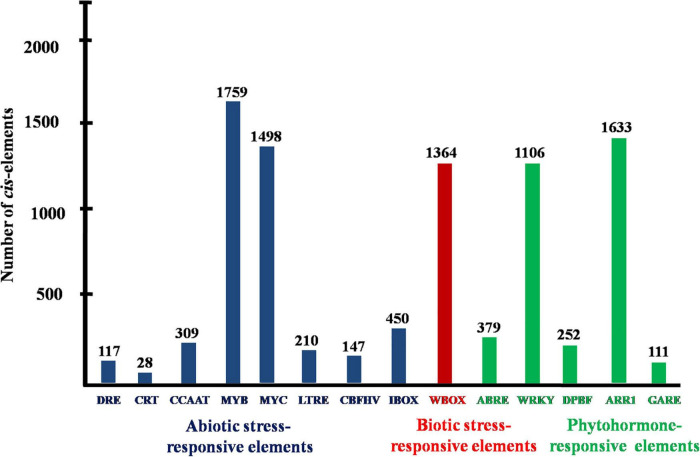
Conserved putative *cis*-acting elements of sorghum K^+^ transport gene homologs. DRE, dehydration-responsive elements; CRT, low-temperature responsive element; CCAAT, promoter of heat shock protein; MYB, responsive to drought and ABA; MYC, response to drought, cold and ABA; LTRE, low temperature and cold-responsive; CBFHV, dehydration-responsive element; IBOX, light regulation; WBOX, transcriptional repressor ERF3 gene; ABRE, early responsive to dehydration; WRKY, transcriptional repressor of the gibberellin; DPBF, ABA; AAR1, cytokinin-regulated transcription factor; GARE, gibberellic acid-responsive elements.

**FIGURE 4:**
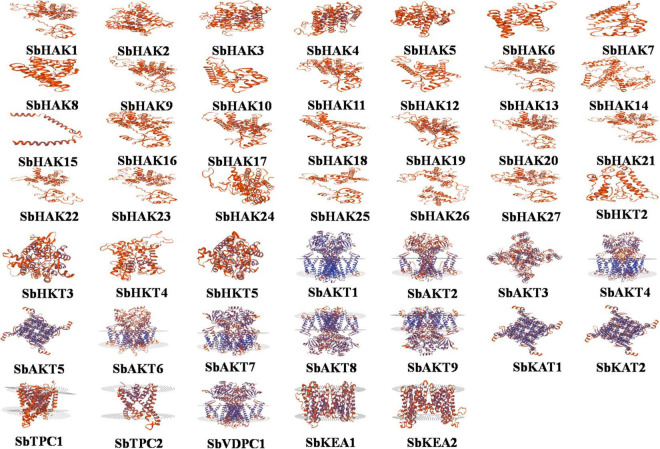
Structural analysis of 47 modeled sorghum K^+^ transport proteins. Sb, *Sorghum bicolor*; HAK, high affinity potassium; KEA, K^+^ efflux antiporter; VDPC, voltage-dependent potassium channel; TPC, two-pore channels.

**FIGURE 5:**
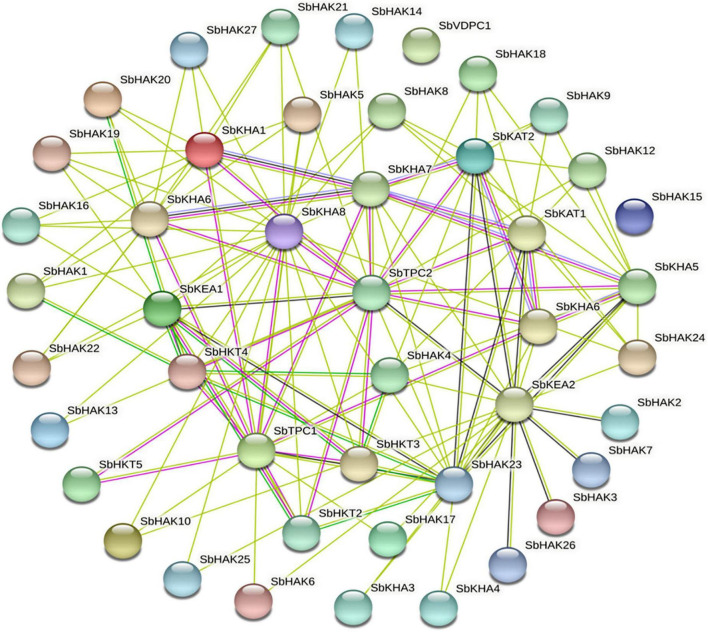
String analysis of sorghum K^+^ transport homologs. All the proteins displayed the interacting partners except SbHAK15 and SbVDPC1. Sb, *Sorghum bicolor*; HAK, high affinity potassium; KEA, K^+^ efflux antiporter; VDPC, voltage-dependent potassium channel; TPC, two-pore channels.

Promoter analysis revealed the presence of biotic, abiotic, and phytohormone-responsive putative *cis*-elements (Supplementary Table 6). Different abiotic stress elements like DRE, CRT, CCAAT, MYB, MYC, LTRE, CBHFV, and IBOX have been identified (Figure 3). MYB represented the highest number of elements in all the transporters indicating their involvement in the stress tolerance. WBOX, the biotic stress-responsive element has also been recognized in all the K^+^ transporters. Most transporters have phytohormone-responsive elements like ABRE, WRKY, DPBF, ARR1, and GARE. ARR1, the cytokinin-responsive elements have been found as the highest number of elements among the phytohormone-responsive elements and identified in all the transporter and channel gene homologs (Figure 3). Such *cis*-element studies are essential since they might contribute to find out the functional regulation of KT/HAK/KUP gene family members in sorghum. 3D structures of K^+^ transport proteins were predicted with the best PDB templates (Figure 4). The template PDB id, template description, chain, model of the oligomer, and their structure validations are represented in the Supplementary Table 7. 3D structures of SbHAK26, SbAKT1, SbAKT2, SbAKT3, SbAKT4, SbAKT5, SbAKT6, SbAKT7, SbAKT8, SbAKT9, SbKAT1, SbKAT2, and SbVDPC1 proteins displayed significant sequence similarity percent ranging from 30.77% (SbHAK26) to 68.61% (SbAKT4). 3D structures of other K^+^ transport proteins did not show any significant (< 30%) sequence similarity (Supplementary Table 7). All the generated Ramachandran plots for structure validation are represented as Supplementary Figure 1. In the predicted PPI map, sorghum K^+^ proteins displayed interactions with several other K^+^ proteins. A total of 46 K^+^ proteins, except SbAKT9 were found in the PPI map (Figure 5). All the 46 proteins have been displayed as 46 nodes with 193 edges. Each protein showed more than one interactant (Supplementary Table 8). SbTPC1, SbTPC2, SbHAK23, SbHKT3, SbHKT4, SbAKT1, SbKEA1, SbHAK8, SbKAT1, and SbKAT2 have been found to be the major interacting proteins. SbHAK15 and SbVDPC1 did not show any interactions (Figure 5). All the STRING protein names used for PPIs are available in Supplementary Table 8.

### Evolutionary divergence, chromosomal location and synteny

**FIGURE 6:**
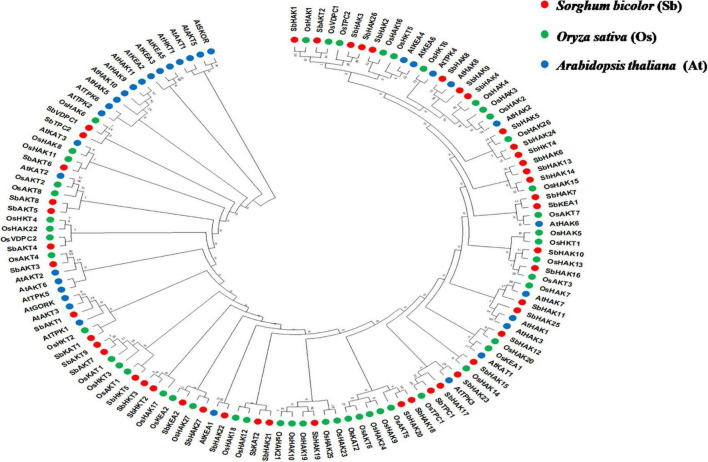
Phylogenetic tree of K^+^ transport proteins between *Sorghum bicolor* (Sb), *Oryza sativa* (Os), and *Arabidopsis thaliana* (At). Predicted amino acid sequences were used for construction of the tree. The tree was constructed by the Neighbor Joining method using MEGA-X. Values indicate the number of times (as a percentage) that each branch topology was found during bootstrap analysis. Sb, *Sorghum bicolor*; HAK, high affinity potassium; KEA, K^+^ efflux antiporter; VDPC, voltage-dependent potassium channel; TPC, two-pore channels.

**FIGURE 7:**
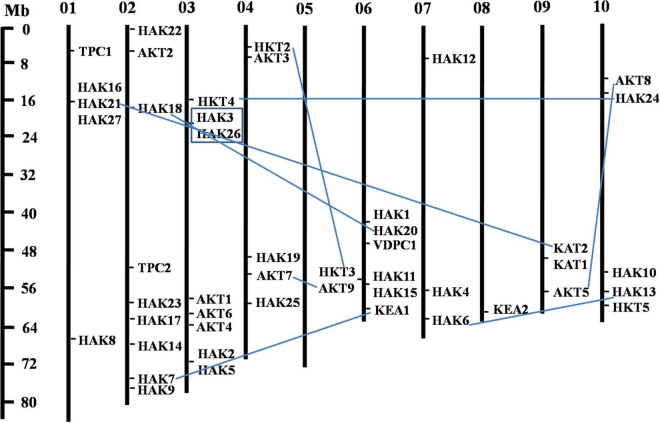
Physical mapping of sorghum K^+^ transport gene homologs. The 9 paralog gene pairs are represented in blue color. Of the 9 paralogs, 8 have been identified as segmental (SbHAK24 and SbHKT4, SbHAK6 and SbHAK13, SbHAK7 and SbKEA1, SbHAK18 and SbHAK20, SbHAK21 and SbKAT2, SbHKT2 and SbHKT3, SbAKT7 and SbAKT9, and SbAKT8 and SbAKT5) represented as lines and 1 as regional (SbHAK3 and SbHAK26) represented as box. Sb, *Sorghum bicolor*; HAK, high affinity potassium; KEA, K^+^ efflux antiporter; VDPC, voltage-dependent potassium channel; TPC, two-pore channels.

**FIGURE 8:**
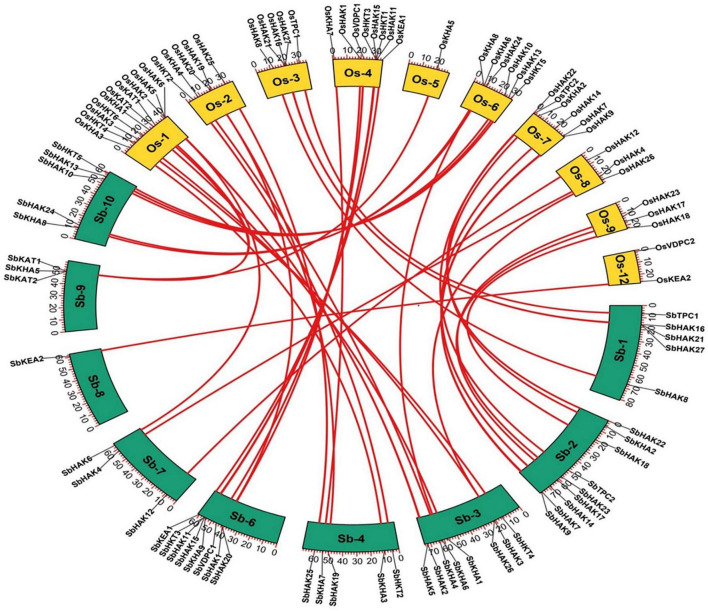
Synteny analysis of K^+^ transporter genes in *Sorghum bicolor* and *Oryza sativa*. The map was built with TB tools software. Sb, *Sorghum bicolor*; Os, *Oryza sativa*; HAK, high affinity K^+^; KEA, K^+^ efflux antiporter; VDPC, voltage-dependent K^+^ channel; TPC, two-pore channels.

The phylogenetic tree revealed the evolutionary relationship of K^+^ transport homologs of *Sorghum bicolor* with *Oryza sativa* and *Arabidopsis thaliana* (Figure 6 and Supplementary Table 9). A total of 9 paralogs have been identified (Figure 7), 1 recognized as regional (SbHAK3 and SbHAK26) and 8 as segmental (SbHAK24 and SbHKT4, SbHAK6 and SbHAK13, SbHAK7 and SbKEA1, SbHAK18 and SbHAK20, SbHAK21 and SbKAT2, SbHKT2 and SbHKT3, SbAKT7 and SbAKT9, and SbAKT8 and SbAKT5) duplications. Sorghum showed 21 ortholog pairs (Figure 6), 18 with *Oryza* (SbHAK1 and OsHAK1, SbAKT2 and OsVDPC1, SbHAK2 and OsHAK16, SbHAK4 and OsHAK4, SbHAK5 and OsHAK26, SbHAK14 and OsHAK15, SbHAK10 and OsHAK13, SbHAK16 and OsAKT3, SbHAK12 and OsHAK20, SbHAK15 and OsHAK14, SbTPC1 and OsTPC1, SbHAK19 and OsHAK19, SbHAK27 and OsHAK27, SbKEA2 and OsKEA2, SbKAT1 and OsHKT2, SbAKT3 and OsAKT4, SbAKT4 and OsVDPC2, and SbAKT6 and OsHAK11) and 3 with *Arabidopsis* (SbHAK8 and AtHAK8, SbHAK23 and AtTPK3, and SbHAK22 and AtKEA1). All the 9 sorghum paralogs (SbHAK3 and SbHAK26, SbHAK24 and SbHKT4, SbHAK6 and SbHAK13, SbHAK7 and SbKEA1, SbHAK18 and SbHAK20, SbHAK21 and SbKAT2, SbHKT2 and SbHKT3, SbAKT7 and SbAKT9, and SbAKT8 and SbAKT5) display substitution rate < 1. The lowest d_*N*_/d_*S*_ (0.0010) were observed in the regional paralog (SbHAK3 and SbHAK26) and the highest d_*N*_/d_*S*_ (0.3651) in segmental paralog (SbHAK24 and SbHKT4) gene pairs (Table 2) respectively. Sorghum K^+^ transport gene homologs have been mapped onto *Oryza* (Figure 8). *S. bicolor* chromosome 2 displays 9 (highest) homologs followed by chromosome 3 and 6 with 8 homologs each, chromosome 1, 4, and 10 with 5 homologs each, chromosome 7 and 9 with 3 homologs each, and chromosome 8 with 1 homolog respectively. Similarly, *O. sativa* displays 10 homologs on chromosome 1, followed by chromosome 4 with 8 homologs, chromosomes 6 and 7 with 6 homologs each, chromosomes 2 and 3 with 5 homologs each, chromosomes 8 and 9 with 3 homologs each, chromosome 12 with 2 homologs, and chromosome 5 with 1 homolog. *O. sativa* and *S. bicolor* chromosomes 1 and 2 show the highest number of homologs with 10 and 9, respectively (Figure 8). Chromosome 5 of sorghum, 10 and 11 of rice do not contain any of the K^+^ gene homologs. A correspondence matrix was created and automated name-based and synteny maps were generated (Figure 8). The links on the chromosomes represent the gene homologs in sorghum and rice (Figure 8).

**TABLE 2:** Non-synonymous and synonymous substitution rates of sorghum paralog genes.

Gene 1	Gene 2	d_*N*_	d_*S*_	d_*N*_/d_*S*_
SbHAK3	SbHAK26	0.0000	0.0067	0.0010
SbHAK24	SbHKT4	1.4970	4.1006	0.3651
SbHAK6	SbHAK13	0.8725	48.9776	0.0178
SbHAK7	SbKEA1	1.7874	41.0903	0.0435
SbHAK18	SbHAK20	0.6416	46.0156	0.0139
SbHAK21	SbKAT2	1.8058	33.4346	0.0540
SbHKT2	SbHKT3	11.1872	17.6762	0.6329
SbAKT7	SbAKT9	0.0000	0.0000	0.2325
SbAKT8	SbAKT5	0.7637	67.2009	0.0114

dS, synonymous substitution; dN, non-synonymous substitution; Sb, Sorghum bicolor; HAK, high affinity potassium; KEA, K^+^ efflux antiporter.

### Digital expressions and quantitative expression analysis of sorghum K^+^ transport gene homologs under abiotic stress conditions in different tissues

**FIGURE 9:**
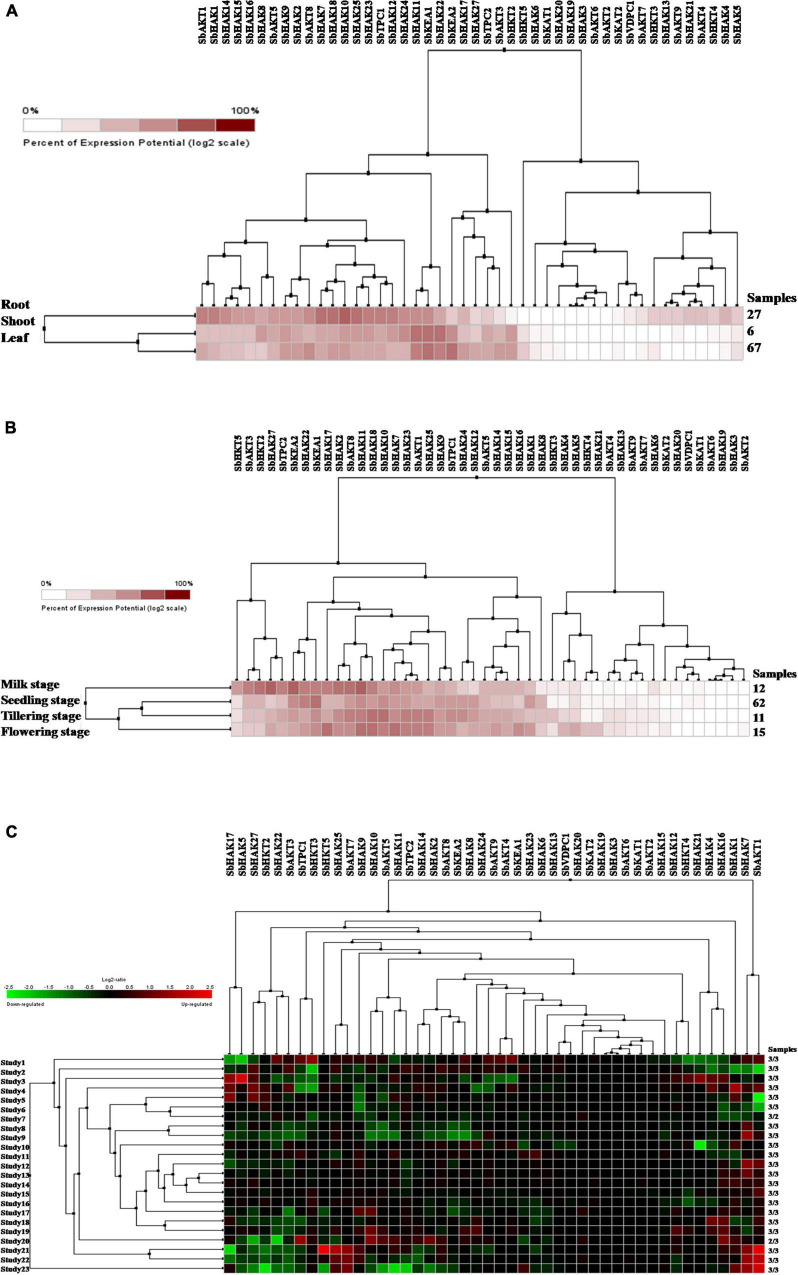
Digital expression profile of sorghum K^+^ transport genes in different tissues, developmental stages under cold and drought stresses **(A)** K^+^ transport in anatomical tissues, **(B)** K^+^ transport in developmental stages, **(C)** K^+^ transport gene expressions under cold and drought stresses. Sb, *Sorghum bicolor*; HAK, high affinity potassium; KEA, K^+^ efflux antiporter; VDPC, voltage-dependent potassium channel; TPC, two-pore channels.

Digital expression of all the 46 K^+^ transport genes was analyzed in root, shoot, and leaf tissues exposed to cold and drought stress conditions. In anatomical tissues, high expression levels of K^+^ transport genes were noticed in root compared shoot, and leaf tissues (Figure 9A). In root tissues, *SbHAK7*, *SbHAK18*, *SbHAK10*, and *SbHAK25*, in shoot tissues, *SbHAK11*, *SbKEA1*, and *SbHAK22*, *SbHAK11*, *SbKEA1*, and *SbKEA2* displayed high expressions respectively (Figure 9A). In developmental stages high expression levels of K^+^ transport genes were noticed in milk stage, flowering stage, tillering stage and seedling stage (Figure 9B). In milk stage *SbHAK27* and *SbKEA2*, in the flowering stage *SbHAK17*, *SbHAK11*, *SbHAK18*, and *SbHAK7*, in tillering stage *SbHAK18*, *SbHAK10*, and 23 genes displayed elevated expression levels than other transport genes (Figure 9B). Differential expression profile of K^+^ transport genes was noticed in cold and drought conditions (Figure 9C). Higher expression levels were noticed in cold stress compared to drought stress. *SbAKT1*, *SbHAK7*, *SbHKT5*, *SbHAK25*, and *SbAKT7* genes have higher expression levels in cold stress. SbHAK5 and SbHAK17 genes have higher expression in drought stress (Figure 9C).

**FIGURE 10:**
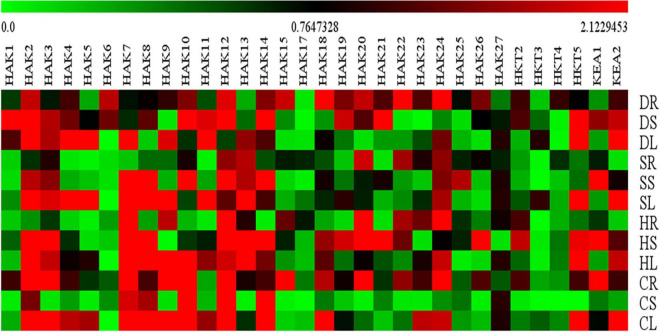
Relative expression analysis of sorghum K^+^ transporter gene homologs. Sorghum transporter expressions during salt, drought, heat, and cold stresses. Relative expression of transporters is shown during different stress conditions compared to its corresponding controls. Values represent the expression levels obtained after normalizing against control tissues. All samples were analyzed in triplicate, in two independent experiments. Names on the vertical axis indicate the tissues and the horizontal axis represents various genes. R, root; S, stem; L, leaf; S, salt; D. drought; H, heat; C, cold. Each color represents the relative expression levels of the transcripts. Sb, *Sorghum bicolor*; HAK, high affinity potassium; KEA, K^+^ efflux antiporter; VDPC, voltage-dependent potassium channel; TPC, two-pore channels.

Expression levels of only 32 K^+^ transport gene homologs (*SbHAK1, SbHAK2, SbHAK3, SbHAK4, SbHAK5, SbHAK6, SbHAK7, SbHAK8, SbHAK9, SbHAK10, SbHAK11, SbHAK12, SbHAK13, SbHAK14, SbHAK15, SbHAK17, SbHAK18, SbHAK19, SbHAK20, SbHAK21, SbHAK22, SbHAK23, SbHAK24, SbHAK25, SbHAK26, SbHAK27, SbHKT2, SbHKT3, SbHKT4, SbHKT5, SbKEA1*, and *SbKEA2*) were analyzed in sorghum root, stem, and leaf tissues subjected to salt, drought, heat, and cold stresses and shown in the heat map (Figure 10). qRT-PCR for other 15 K^+^ transport gene homologs could not be performed due to high sequence similarity. The homologs displayed differential gene expression in different tissues (Supplementary Table 10). *SbHAK2*, *SbHAK20*, *SbHAK5*, and *SbHAK3* showed markedly increased expressions under salt, heat, drought and cold stresses respectively. Among the stress treatments, a 13.86-fold increase in transcript levels was observed in *SbHAK3* in cold-stressed roots, followed by strong upregulation of *SbHAK2* (13.39-folds increase) under salt stress in the leaves. *SbHAK7* was enhanced by 11.95-folds in the leaves exposed to salt stress. Similarly, *SbHAK12*, *SbHAK20*, and *SbHAK21* displayed 10.19, 10.48, and 10.14-folds enhanced activity in stem tissues subjected to salt (SbHAK12) and heat stresses (*SbHAK20*, *SbHAK21*) respectively. While *SbHAK15* showed 9.36-fold increase in roots exposed to cold stress, *SbHAK8*, *SbHAK14*, and *SbHAK10* exhibited higher activities in stems treated with salt and cold stresses respectively. Out of all gene homologs, *SbHAK2*, *3*, *7*, *8*, *10*, *12*, *14*, *15*, *20*, *21*, and *14* recorded markedly high expressions in compared to other *SbHAK*, *SbHKT*, and *SbKEA* members. Transcripts *SbHAK7*, *SbHAK8*, *SbHAK9*, *SbHAK10*, *SbHAK11*, *SbHAK12*, *SbHAK13*, and *SbHAK14* are highly upregulated under salt, heat, and cold, while *SbHAK1*, *SbHAK4*, *SbHAK5*, *SbHAK18*, *SbHAK22*, and *SbHAK27* (4.55–7.94-folds) were well expressed under drought stress conditions. *SbHAK17* has the lowest level of expression among all the genes across different stresses and tissues (Figure 10). Transcript expressions were increased in stem and leaf tissues subjected to high temperature stress especially in most of *SbHAKs*. Among the *SbHKTs*, the transcript level of *SbHKT5* was superior (7.17-fold increase) in leaves exposed to salt stress. Similarly, *SbKEA1* expression was significantly high (10.14-folds) under salt stress in the stems of Sorghum. Activity of *SbKEA2* was 11.2-folds higher in salt-stressed leaves.

## Discussion

K^+^ plays a pivotal role as a constituent of the plant structure, in ion homeostasis, salt tolerance, plant growth, development, transport, aside from acting as a signaling molecule (Feng et al., 2019; Hussain et al., 2021). It has a regulatory function in many physiological and biochemical processes such as protein synthesis, and activation of enzymes (Hasanuzzaman et al., 2018). K^+^ is available to plants only in ionic form and higher K^+^/Na^+^ has been recognized unequivocally as a crucial molecule for maintaining electro-neutrality of the cells (Hussain et al., 2021). Under saline and water deficient conditions, K^+^ maintains ion homeostasis and modulates the osmotic balance. Further, K^+^ participates in stomatal regulation during drought stress and increases the antioxidative ability of the plants (Hasanuzzaman et al., 2018). Since many homologs have been detected in plants, we need to understand which of the homologs perform the crucial processes of plant growth, abiotic stress, and under K^+^ deprivation conditions (Hamamoto et al., 2008; Jiang et al., 2021). In the present investigation, a total of 47 K^+^ transporter gene homologs (Table 1) were discovered in all including 33 K^+^ transporters (27 HAKs, 4 HKTs, and 2 KEAs) and the remaining 14 (9 AKTs, 2 KATs, 2 TPCs, and 1 VDPC) as K^+^ channels in *S. bicolor*.

### Characterization of K^+^ transport gene homologs in sorghum

Sorghum has 47 K^+^ transporter gene homologs (Table 1 and Figure 1) in comparison with 43 in *Gossypium raimondii*, (Azeem et al., 2022), 39 in *Cajanus cajan* (Siddique et al., 2021), and 36 in *Cicer arietinum* (Azeem et al., 2018). Nevertheless, these numbers are lower than that of *Glycine max*, where 70 homologs have been detected (Rehman et al., 2017), and 49 in *Oryza sativa* (Amrutha et al., 2007). The 27 genes encoding HAK transporters in Sorghum (Figure 1) are similar in the number of HAK encoding genes detected in *Zea mays* and *Hordeum vulgare* (Zhang et al., 2012; Cai et al., 2021). *Triticum aestivum* and *Pyrus betulifolia* have 56 HAK transporters each (Cheng et al., 2018; Li Y. et al., 2018) followed by 41 in *Nicotiana tabacum (*Song et al., 2019), 31 in *Populus trichocarpa* (He et al., 2012), 30 in *Saccharum spontaneum* (Feng et al., 2020b), 29 in *Glycine max (*Rehman et al., 2017*)*, 26 in *Oryza sativa* (Amrutha et al., 2007), 22 in *Salix purpurea* and *Ipomoea batatas* (Liang et al., 2020; Jin et al., 2021), 21 in *Populus trichocarpa*, *Prunus persica*, *Manihot esculenta*, and *Camellia sinensis* (He et al., 2012; Song et al., 2015; Ou et al., 2018; Yang T. et al., 2020), 19 in *Solanum lycopersicum* (Hyun et al., 2014), 17 in *Oryza sativa* and *Cajanus cajan* (Banuelos et al., 2002; Siddique et al., 2021), 16 in *Prunus persica* and *Gossypium raimondii* (Song et al., 2015; Azeem et al., 2022), and 13 in *Arabidopsis thaliana* (Ahn et al., 2004). These studies indicate that K^+^ transport genes are highly conserved in plants during evolution. A total of 21, 24, 45, and 44 HAK/KUP/KT genes were identified in *Gossypium hirsutum*, *Gossypium barbadense*, *Gossypium raimondii*, and *Gossypium arboretum* genomes respectively (Yang X. et al., 2020). The higher number of K^+^ transporter homologs in *Triticum* is due to its ploidy nature (Kyriakidou et al., 2018*)*. Sorghum has shared 4 HKT encoding genes with *Glycine max (*Rehman et al., 2017*)*. *Oryza sativa* has 8 HKTs, followed by 4 in *Sorghum bicolor* and *Glycine max*, 2 HKTs in *Gossypium raimondii*, *Cajanus cajan*, and *Cicer arietinum* (Amrutha et al., 2007; Rehman et al., 2017; Azeem et al., 2018, 2022; Siddique et al., 2021). *Glycine max* has the highest number of KEA transporters (12) (Rehman et al., 2017), followed by 7 KEAs in *Gossypium raimondii* (Azeem et al., 2022), 6 KEAs in *Cicer arietinum* and *Cajanus cajan* (Azeem et al., 2018; Siddique et al., 2021), but 2 KEAs in sorghum, and 1 KEA in *Oryza sativa* (Amrutha et al., 2007). The number of K^+^ channels identified in sorghum corroborates the previously reported genomes. A total of 14 K^+^ channels were identified in sorghum (9 AKTs, 2 KATs, 2TPKs, and 1 VDPC), an equal number in *Oryza sativa* (14 AKTS) (Amrutha et al., 2007), belonging to the same family. But *Cajanus cajan* has 9 shakers and 5 TPKs (Siddique et al., 2021). However, 25 K^+^ channel gene homologs (16 VDPCs, 9 TPK/KCO) have been reported in *Glycine max* (Rehman et al., 2017) and 18 (11 shakers and 7 TPKs/KCO) in *Gossypium raimondii* (Azeem et al., 2022).

The conserved domains of K^+^ transporter system *viz*., K-trans, TrK, KEA, voltage-dependent K^+^ channel, KHA, and Two Pore Potassium (TPK) channel were identified in sorghum (Table 1) which corroborates the identified K^+^ genes in other plant genomes such as *Oryza sativa* (Amrutha et al., 2007), *Glycine max* (Rehman et al., 2017), *Cicer arietinum* (Azeem et al., 2018), *Cajanus cajan* (Siddique et al., 2021), and *Gossypium raimondii* (Azeem et al., 2022). The consensus motif GVVYGDLGTSPLY (Rodríguez-Navarro, 2000) was identified in all the sorghum HAK transporters except *SbHAK5*, *SbHAK12*, and *SbHAK22* (Figure 2). Similar results were also reported in *Oryza* (Amrutha et al., 2007), *Cicer arietinum* (Azeem et al., 2018), *Cajanus cajan* (Siddique et al., 2021), and *Gossypium raimondii* (Azeem et al., 2022). The motif GGTFALYSLLCR was detected in *Arabidopsis thaliana* (Ahn et al., 2004), *Cicer arietinum* (Azeem et al., 2018), *Cajanus cajan* (Siddique et al., 2021), *Gossypium* (Azeem et al., 2022) was also noticed in sorghum inferring its evolutionary conservation. The conserved K^+^ channel motif GYGD (Kuang et al., 2015) has been observed in all the sorghum K^+^ channels (Figure 2), identical to that of *Oryza sativa* (Amrutha et al., 2007), *Cicer arietinum* (Azeem et al., 2018), *Cajanus cajan* (Siddique et al., 2021), and *Gossypium* (Azeem et al., 2022). Most of the sorghum HAK transporters are localized on plasma membrane (Table 1) akin to *Triticum aestivum* (Cheng et al., 2018), *Saccharum spontaneum* (Feng et al., 2020b), *Salix purpurea* (Liang et al., 2020), *Camellia sinensis* (Yang T. et al., 2020), *Hordeum vulgare* (Cai et al., 2021), and *Ipomoea batatas* (Jin et al., 2021). *A. thaliana* AtHAK5 has been associated with the uptake of Na^+^ (Wang Q. et al., 2015). This implies that these porters subscribe to the accumulation of Na^+^ under saline conditions. Since restricting Na^+^ uptake determines salt tolerance, care must be taken while breeding the crop plants for salt stress tolerance. Also, HKTs are involved in loading Na^+^ into the xylem. Zhu et al. (2016) also noticed a link between SOS1 and HKT pathways for salt stress in wheat. These studies indicate the HKTs critical role during salt stress tolerance. K^+^ efflux antiporters KEA1 and KEA2 have been found in inner envelop membrane in *A. thaliana*, and the loss of function mutants influence both ROS and reactive nitrogen species (RNS). Double knock-out mutants of *kea1kea2* elicited an alteration of the ROS homeostasis. However, nitric oxide (NO) content has negatively affected photosynthesis increasing photorespiratory activity (Sánchez-McSweeney et al., 2021). The studies infer that KEAs maintain chloroplast osmotic balance. In sorghum, SbKEA1 is localized on chloroplast membranes and is perhaps involved in the regulation of thylakoid and stromal pH (Sánchez-McSweeney et al., 2021).

### Analysis of putative *cis*-elements and 3D protein structures, and interactions

Hyun et al. (2014) and Assaha et al. (2017) reported the involvement of *cis*-regulatory elements in abiotic stress tolerance and in K^+^ homeostasis. Similarly, analysis of promoter sequences of K^+^ transport gene homologs in sorghum revealed the presence of *cis*-elements which may be involved in diverse abiotic stress tolerances. This prediction is in line with that of *Cicer arietinum* (Azeem et al., 2018), *Cajanus cajan* (Siddique et al., 2021), and *Gossypium raimondii* (Azeem et al., 2022). In the promoter regions, regulatory elements like ABRE, MYB, MYC, GARE, WBOX, LTRE, and CCAAT have been noticed in *Cicer arietinum* (Azeem et al., 2018), *Pyrus betulifolia* (Li Y. et al., 2018), *Manihot esculenta* (Ou et al., 2018), *Nicotiana tabacum* (Song et al., 2019), *Salix purpurea* (Liang et al., 2020), *Camellia sinensis* (Yang T. et al., 2020), *Hordeum vulgare* (Cai et al., 2021), *Cajanus cajan* (Siddique et al., 2021), *Ipomoea batatas* (Jin et al., 2021), and *Gossypium* species (Yang X. et al., 2020; Azeem et al., 2022) indicating the involvement of K^+^ transport gene homologs in abiotic stress tolerance. Aside abiotic stress-responsive elements, promoter analysis also revealed the presence of biotic stress-responsive and phytohormone stress-responsive elements in sorghum (Figure 3). The predicted elements indicate that K^+^ transporters are implicated in biotic stress response and their cross-talk with hormones during stress. Protein models (Figure 4) help to understand structure-function relationships (Rasheed et al., 2020). Protein-protein interactions (PPIs) of sorghum (Figure 5) displayed interaction with other K^+^ transporters and channels like what has been noticed in *Cajanus cajan* (Siddique et al., 2021). The SbHAK transporters also interacted with other SbHAK transporters like *Ipomoea batatas* HAK transporters (Jin et al., 2021).

### Evolutionary divergence and comparative analysis

Phylogenetic analysis revealed the close relationship of *Sorghum bicolor* K^+^ transporters with *Oryza sativa* (Amrutha et al., 2007) than to *Arabidopsis thaliana* (Figure 6). A comparative phylogenetic analysis of K^+^ transporters has been carried out in *Oryza sativa* (Amrutha et al., 2007), *Glycine max* (Rehman et al., 2017), *Cicer arietinum* (Azeem et al., 2018), *Cajanus cajan* (Siddique et al., 2021), and *Gossypium raimondii* (Azeem et al., 2022). Similarly, comparative studies of HAK transporters have been reported in *Oryza sativa* (Banuelos et al., 2002), *Arabidopsis thaliana* (Ahn et al., 2004), *Populus trichocarpa* (He et al., 2012), *Zea mays* (Zhang et al., 2012), *Solanum lycopersicum* (Hyun et al., 2014), *Prunus persica* (Song et al., 2015), *Glycine max* (Rehman et al., 2017), *Triticum aestivum* (Cheng et al., 2018) and *Pyrus betulifolia* (Li Y. et al., 2018), *Manihot esculenta* (Ou et al., 2018), *Nicotiana tabacum* (Song et al., 2019), *Saccharum spontaneum* (Feng et al., 2020b), *Salix purpurea* (Liang et al., 2020), *Hordeum vulgare* (Cai et al., 2021), *Ipomoea batatas* (Jin et al., 2021), and *Camellia sinensis* (Yang T. et al., 2020). Most sorghum K^+^ homologs showed high gene conservation with *Oryza sativa* (Figure 6) as both share common ancestor (Wang X. et al., 2015). The tree indicated 9 paralog and 21 ortholog (18 with *Oryza sativa* and 3 with *Arabidopsis thaliana*) groups. Chromosomal distribution and synteny analyses revealed the presence of 1 regional (*SbHAK3* and *SbHAK26*) and 8 segmental (*SbHAK24* and *SbHKT4*, *SbHAK6* and *SbHAK13*, *SbHAK7* and *SbKEA1*, *SbHAK18* and *SbHAK20*, *SbHAK21* and *SbKAT2*, *SbHKT2* and *SbHKT3*, *SbAKT7* and *SbAKT9*, and *SbAKT8* and *SbAKT5*) duplication gene pairs. Such gene duplication events have also been reported in *Ipomoea batatas* HAK transporters (Jin et al., 2021). All the 9 sorghum paralogs (*SbHAK3* and *SbHAK26*, *SbHAK24* and *SbHKT4*, *SbHAK6* and *SbHAK13*, *SbHAK7* and *SbKEA1*, *SbHAK18* and *SbHAK20*, *SbHAK21* and *SbKAT2*, *SbHKT2* and *SbHKT3*, *SbAKT7* and *SbAKT9*, and *SbAKT8* and *SbAKT5*) have the dN/dS value < 1 (Table 2), indicating a purifying Darwinian selection during the evolution of HAK genes (Bowers et al., 2003). The HAK family of *Saccharum spontaneum* (Feng et al., 2020b) and *Ipomoea batatas* (Jin et al., 2021) have also showed the non-synonymous/synonymous value < 1. Uneven distributions of K^+^ transport gene homologs on different chromosomes have been observed in sorghum (Figure 7) similar to *Oryza sativa* (Amrutha et al., 2007), *Glycine max* (Rehman et al., 2017), *Cicer arietinum* (Azeem et al., 2018), *Cajanus cajan* (Siddique et al., 2021), and *Gossypium raimondii* (Azeem et al., 2022). Uneven distribution of HAK/KUP/KT homologs has been observed in angiosperms also (Nieves-Cordones et al., 2016). Gene order conservation of K^+^ gene homologs across sorghum and rice has been identified by circos (Figure 8). K^+^ transport homologs displayed very high conservation between sorghum and rice since they share a common ancestor (Wang X. et al., 2015). Chromosome 2 and 1 of sorghum and rice displayed the highest number of homologs with 9 and 10, respectively. Also, an equal number (3) of K^+^ transport gene homologs was observed on chromosome 9 of sorghum and rice (Figure 8).

### Digital and qRT-PCR transcript patterns of sorghum K^+^ transport genes responding to abiotic stresses

Digital expression of K^+^ transport genes have been identified in different tissues like root, shoot, and leaf (Figure 9A) and in different developmental stages like milk stage, seedling stage, tillering stage, and flowering stage (Figure 9B). Expression of sorghum K^+^ transport genes has reported under cold and drought stresses (Figure 9C). Sorghum transcript analysis revealed that K^+^ transport genes are responsive to different abiotic stresses. qRT-PCR results indicated differential gene expression of sorghum K^+^ transport gene homologs in the root, stem, and leaf tissue treated with salt, drought, heat, and cold stresses (Figure 10). Such a differential gene expression of K^+^ transporters and channels in diverse tissues and under abiotic stresses have been reported in *Triticum aestivum* (Cheng et al., 2018), *Cicer arietinum* (Azeem et al., 2018), *Saccharum spontaneum* (Feng et al., 2020b), *Cajanus cajan* (Siddique et al., 2021), *Ipomoea batatas* (Jin et al., 2021), and *Gossypium raimondii* (Azeem et al., 2022). Reports exist that HAK/KUP/KT family members ameliorate the plants from salt stress. In cotton, GhPOT5, a homolog of *OsHAK1* exhibited significantly higher expression under salt stress in comparison with other genes (Yang X. et al., 2020). Salinity reduces the uptake of K^+^ as evident in rice mutants *Oshak1*, when the levels were below 0.05 mM. Overexpression of *HAK1* resulted in salt stress tolerance in rice (Chen et al., 2015). Hamamoto et al. (2015) noticed that *AtHKT1* provides protection to the leaves under salt stress. In line with this, *SbHKT5* displayed higher activity under salt stress in the present study. Similarly, K^+^ transporter genes were upregulated in the present study under water deficit conditions. Under drought conditions, root growth is restricted and diffusion of K^+^ toward the roots (Wang et al., 2013). Also, long-term exposure to water deficit conditions led to leaf damage due to ROS formation (Wang et al., 2013). In support of this, optimization of K^+^ supply mitigates the damage caused due to the oxidative stress in barley exposed to drought stress (Tavakol et al., 2021). Silencing *HvAKT2* and *HvHAK1* in barley enhanced the ROS (H_2_O_2_) production in PEG-treated leaves (Feng et al., 2020a). In rice, overexpression of *OsHAK1* positively regulates drought stress, while the knockout lines accumulate less K^+^ and more H_2_O_2_ with stunted growth of the plants and less tolerance to drought stress (Chen et al., 2017). These results point out that K^+^ reduces the accumulation of H_2_O_2_ and thus helps the plants during drought stress. Earlier studies also revealed that HAK/KUP/KT family genes improve drought stress tolerance in plants (Wang et al., 2013; Li W. et al., 2018). Further, under water deficit conditions, K^+^ regulates opening of stomata and make the plants adaptive to drought (Tang et al., 2015). K^+^ increases the antioxidant defense in plants under abiotic stress conditions (Wang et al., 2013; Amanullah et al., 2016). Under extreme temperatures, osmolytes accumulate and K^+^ helps to maintain stomatal conductance and therefore avoids the damage (Azedo-Silva et al., 2004; Hasanuzzaman et al., 2018). These studies point out that K^+^ transporter genes play pivotal roles during environmental adversities and impart tolerance to multiple abiotic stresses.

In conclusion, genome-wide analysis of sorghum has led to the identification of 47 K^+^ transport gene homologs; 33 K^+^ transporters (27 HAKs, 4 HKTs, and 2 KEAs) and 14 K^+^ channels (9 AKTs, 2 KATs, 2 TPCs, and 1 VDPC). Gene characterization, conserved domains, motif identifications, localization, phylogenetic analysis revealed the close relation of *Sorghum bicolor* K^+^ transport gene homologs with its relative *Oryza sativa*. Identification of *cis*-acting elements would be helpful to explore further and to manipulate K^+^ porters as well as channels for designing better crops. Gene expression data indicate that such genes can be utilized effectively in breeding programs aimed at abiotic stress tolerance. The results bring forth precious information candidate gene identification for functional analyses and subsequent utilization in genetic engineering, and traditional breeding programs to improve sorghum for abiotic stress tolerance.

## Data availability statement

The datasets presented in this study can be found in online repositories. The names of the repository/repositories and accession number(s) can be found in the article/Supplementary material.

## Author contributions

SAK and PBK designed the experiments. PHK, MN, TDD, AM, and PSR carried out the bioinformatics analysis. SAK and MN performed the qRT-PCR experiments. SAK, PHK, MN, TDD, PSR, and PBK analyzed the data. SAK, RK, and PBK prepared the manuscript and refined it. All authors have read and approved the manuscript.

## Footnotes

1
http://www.gramene.org/

2
http://genes.mit.edu/GENSCAN.html

3
https://www.ncbi.nlm.nih.gov/Structure/cdd/wrpsb.cgi

4
http://smart.embl-heidelberg.de/

5
http://gsds.gao-lab.org/

6
https://wolfpsort.hgc.jp/

7
http://web.expasy.org/protparam/

8
https://services.healthtech.dtu.dk/service.php?NetPhos-3.1

9
https://pepcalc.com/

10
https://meme-suite.org/meme/tools/meme

11
http://mg2c.iask.in/mg2c_v2.1/

12
https://www.dna.affrc.go.jp/PLACE/?action=newplace

13
https://montelionelab.chem.rpi.edu/PSVS/PSVS/

14
https://string-db.org/

15
http://www.bork.embl.de/pal2nal/

16
https://genevestigator.com/gv

## Supplementary material

Supplementary Figure 1 Ramachandran plot validation for 3D structures of K^+^ transport proteins.

Supplementary Table 1 List of qRT-PCR primers used for transcript profiling of *Sb* K^+^ transport genes.

Supplementary Table 2 Nucleotide and protein sequences of *Sb* K^+^ transport homologs with ids.

Supplementary Table 3 The pI, MW, GRAVY, instability, and aliphatic indexes of *Sb* K^+^ transport proteins.

Supplementary Table 4 Amino acid composition and net charge of the *Sb* K^+^ transport genes.

Supplementary Table 5 Types of protein kinases in the phosphorylation of *Sb* K^+^ transport genes.

Supplementary Table 6 Conserved *cis*-acting regulatory elements in *Sb* K^+^ transport gene promoters.

Supplementary Table 7 Modeled *Sb* K^+^ transport proteins using SWISS-MODEL server.

Supplementary Table 8 STRING ids of *Sb* K^+^ transport proteins used for protein-protein interactions.

Supplementary Table 9 List of K^+^ transport genes from *Sorghum bicolor, Oryza sativa*, and *Arabidopsis thaliana* used for construction of phylogenetic tree.

Supplementary Table 10 Relative expression analysis of K^+^ transport gene homologs.
