# Supplementary material for: Genome-wide identification and multiple abiotic stress transcript profiling of potassium transport gene homologs in Sorghum bicolor
Source: Front Plant Sci. 2022 Sep 2;13:965530. doi: 10.3389/fpls.2022.965530 (PMC9478208; doi:10.3389/fpls.2022.965530)
Supplement: Supplementary Figure 1 — Ramachandran plot validation for 3D structures of K+ transport proteins. [file Data_Sheet_1.PDF]

PROCHECK-NMR

Ramachandran Plot

SbHAK1_noHs (1 models)

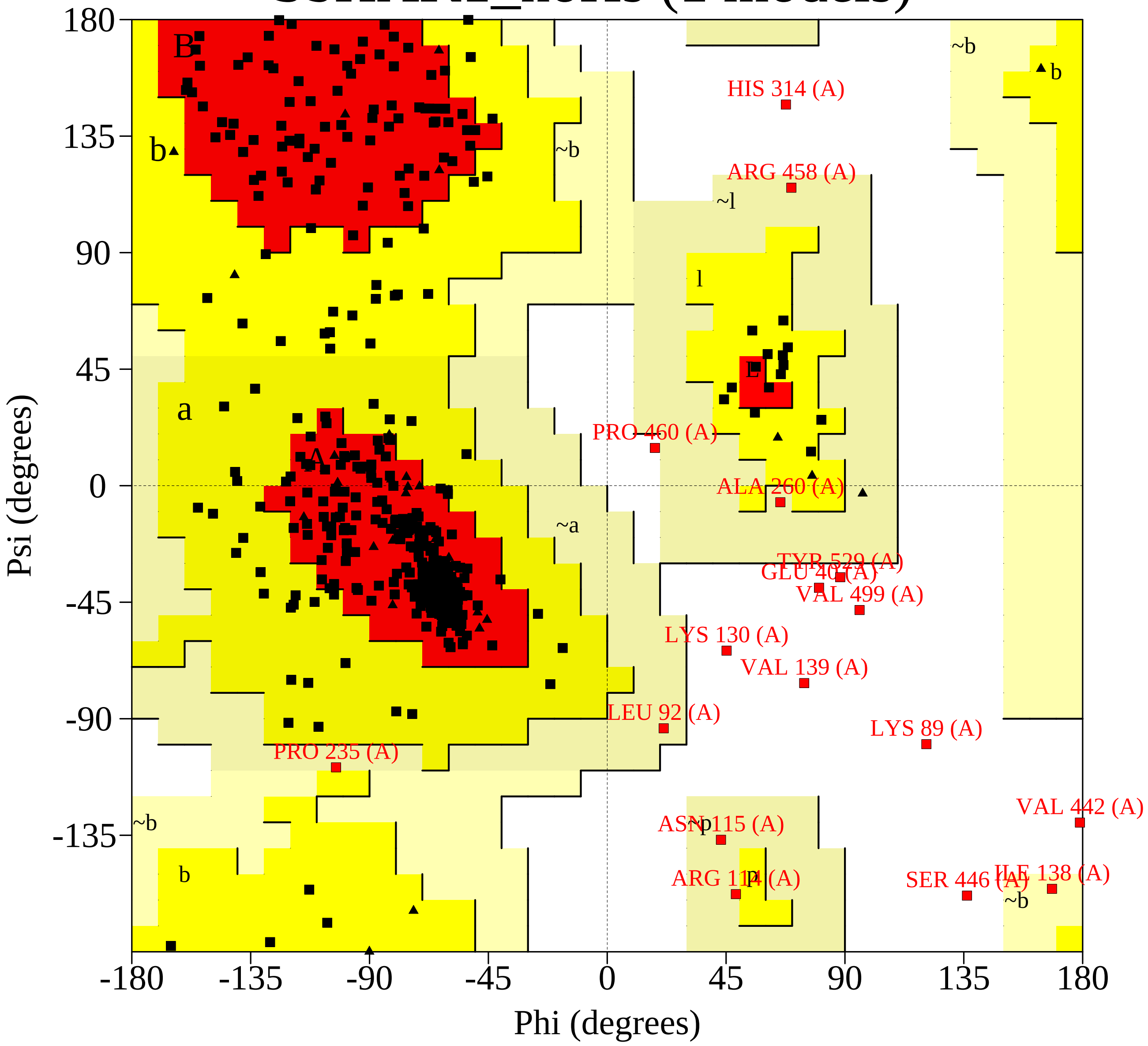

Plot statistics

Residues in most favoured regions [A,B,L]	388	82.6%
Residues in additional allowed regions [a,b,l,p]	67	14.3%
Residues in generously allowed regions [~a,~b,~l,~p]	6	1.3%
Residues in disallowed regions	9	1.9%
	----	------
Number of non-glycine and non-proline residues	470	100.0%
Number of end-residues (excl. Gly and Pro)	2	
Number of glycine residues (shown as triangles)	38	
Number of proline residues	23	

Total number of residues	533	

Based on an analysis of 118 structures of resolution of at least 2.0 Angstroms and R-factor no greater than 20%, a good quality model would be expected to have over 90% in the most favoured regions.

PROCHECK-NMR

Ramachandran Plot

SbHAK2_noHs (1 models)

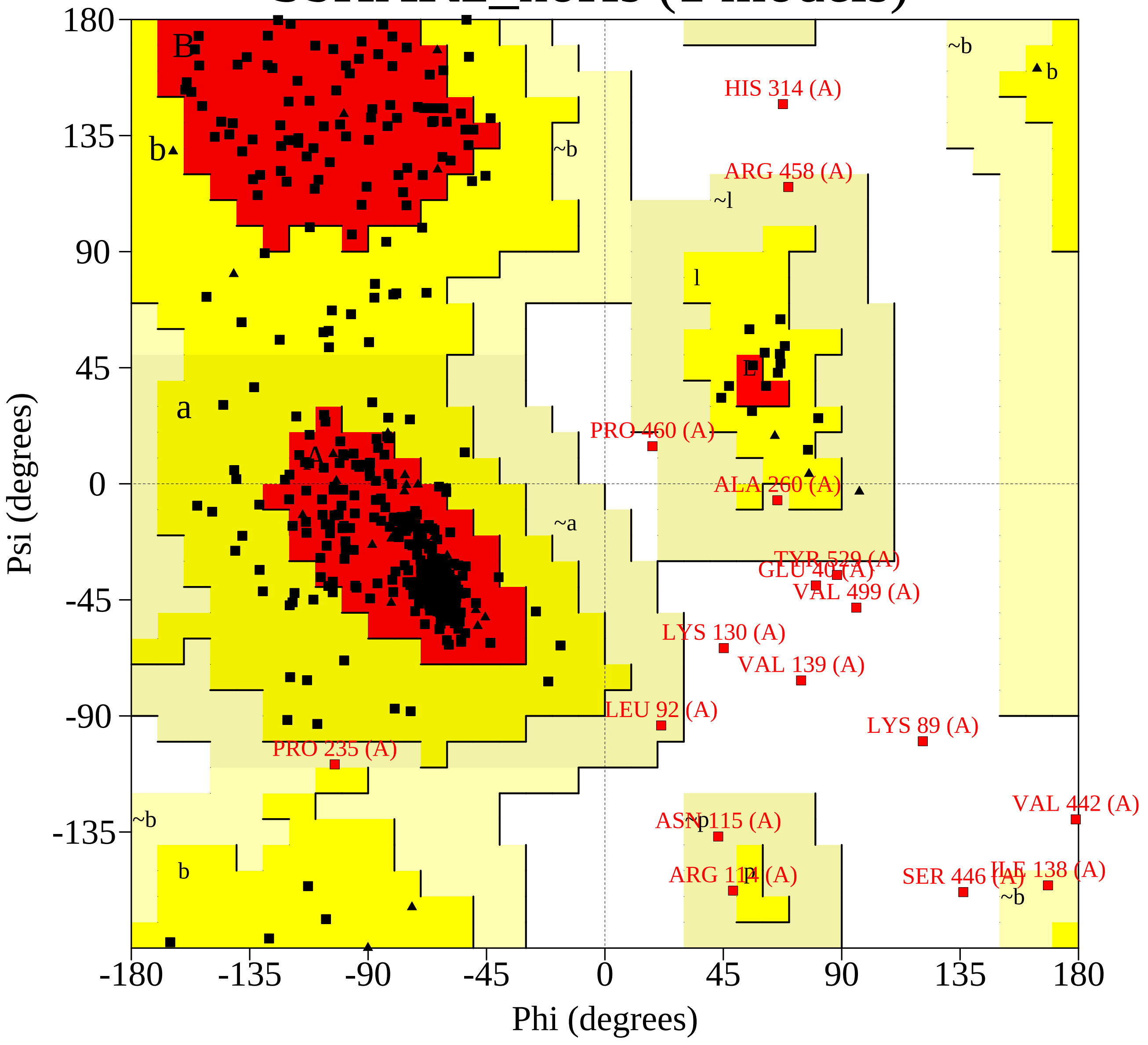

Plot statistics

Residues in most favoured regions [A,B,L]	388	82.6%
Residues in additional allowed regions [a,b,l,p]	67	14.3%
Residues in generously allowed regions [~a,~b,~l,~p]	6	1.3%
Residues in disallowed regions	9	1.9%
	----	------
Number of non-glycine and non-proline residues	470	100.0%
Number of end-residues (excl. Gly and Pro)	2	
Number of glycine residues (shown as triangles)	38	
Number of proline residues	23	

Total number of residues	533	

Based on an analysis of 118 structures of resolution of at least 2.0 Angstroms and R-factor no greater than 20%, a good quality model would be expected to have over 90% in the most favoured regions.

SbHAK2_noHs_01_ramachand.ps

PROCHECK-NMR

Ramachandran Plot

SbHAK3_noHs (1 models)

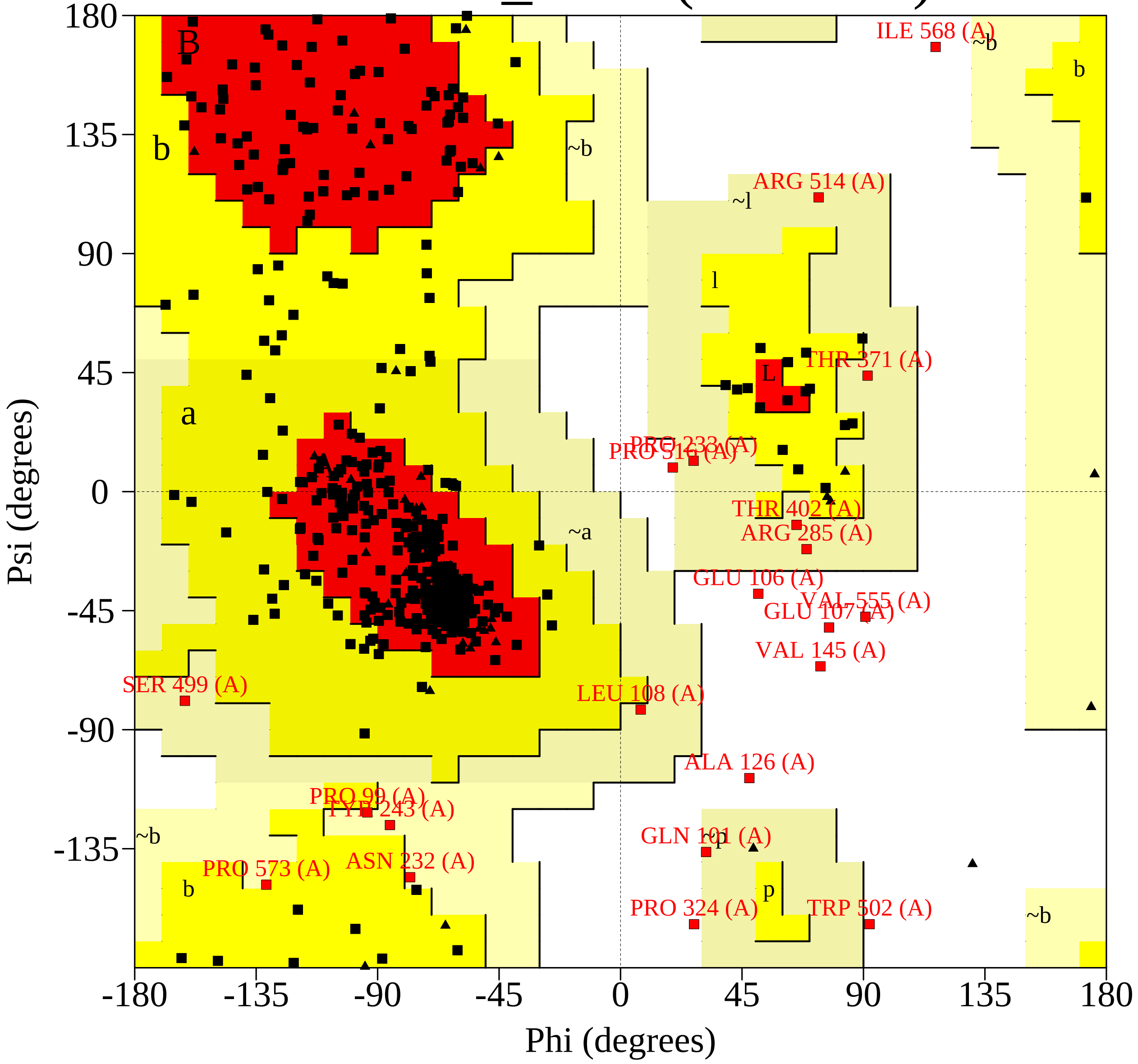

Plot statistics

Residues in most favoured regions [A,B,L]	416	81.7%
Residues in additional allowed regions [a,b,l,p]	77	15.1%
Residues in generously allowed regions [~a,~b,~l,~p]	9	1.8%
Residues in disallowed regions	7	1.4%
	----	------
Number of non-glycine and non-proline residues	509	100.0%
Number of end-residues (excl. Gly and Pro)	2	
Number of glycine residues (shown as triangles)	47	
Number of proline residues	26	

Total number of residues	584	

Based on an analysis of 118 structures of resolution of at least 2.0 Angstroms and R-factor no greater than 20%, a good quality model would be expected to have over 90% in the most favoured regions.

PROCHECK-NMR

Ramachandran Plot

SbHAK4_noHs (1 models)

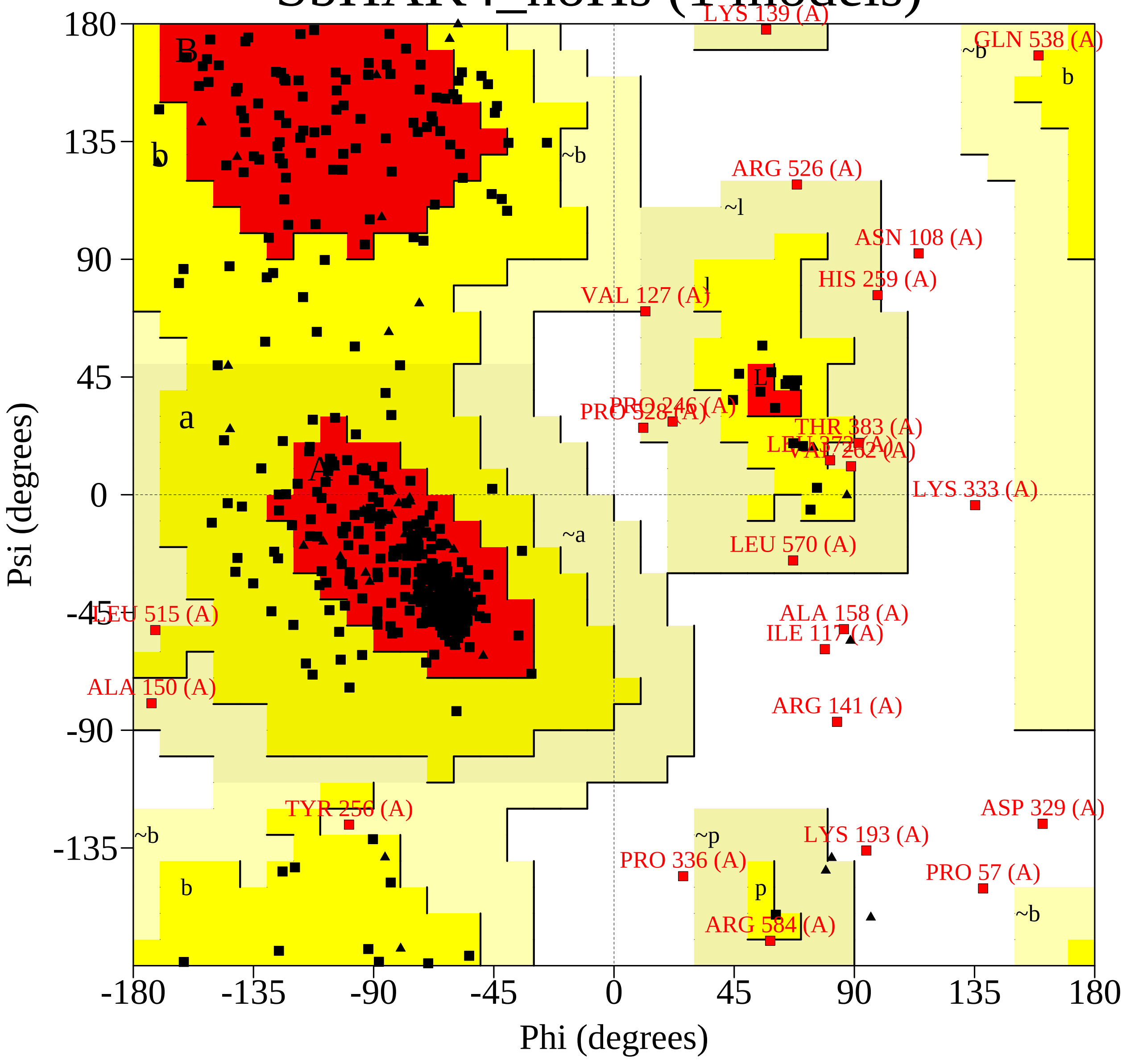

Plot statistics

Residues in most favoured regions [A,B,L]	402	80.9%
Residues in additional allowed regions [a,b,l,p]	75	15.1%
Residues in generously allowed regions [~a,~b,~l,~p]	13	2.6%
Residues in disallowed regions	7	1.4%
	----	------
Number of non-glycine and non-proline residues	497	100.0%
Number of end-residues (excl. Gly and Pro)	2	
Number of glycine residues (shown as triangles)	48	
Number of proline residues	29	

Total number of residues	576	

Based on an analysis of 118 structures of resolution of at least 2.0 Angstroms and R-factor no greater than 20%, a good quality model would be expected to have over 90% in the most favoured regions.

PROCHECK-NMR

Ramachandran Plot

SbHAK5_noHs (1 models)

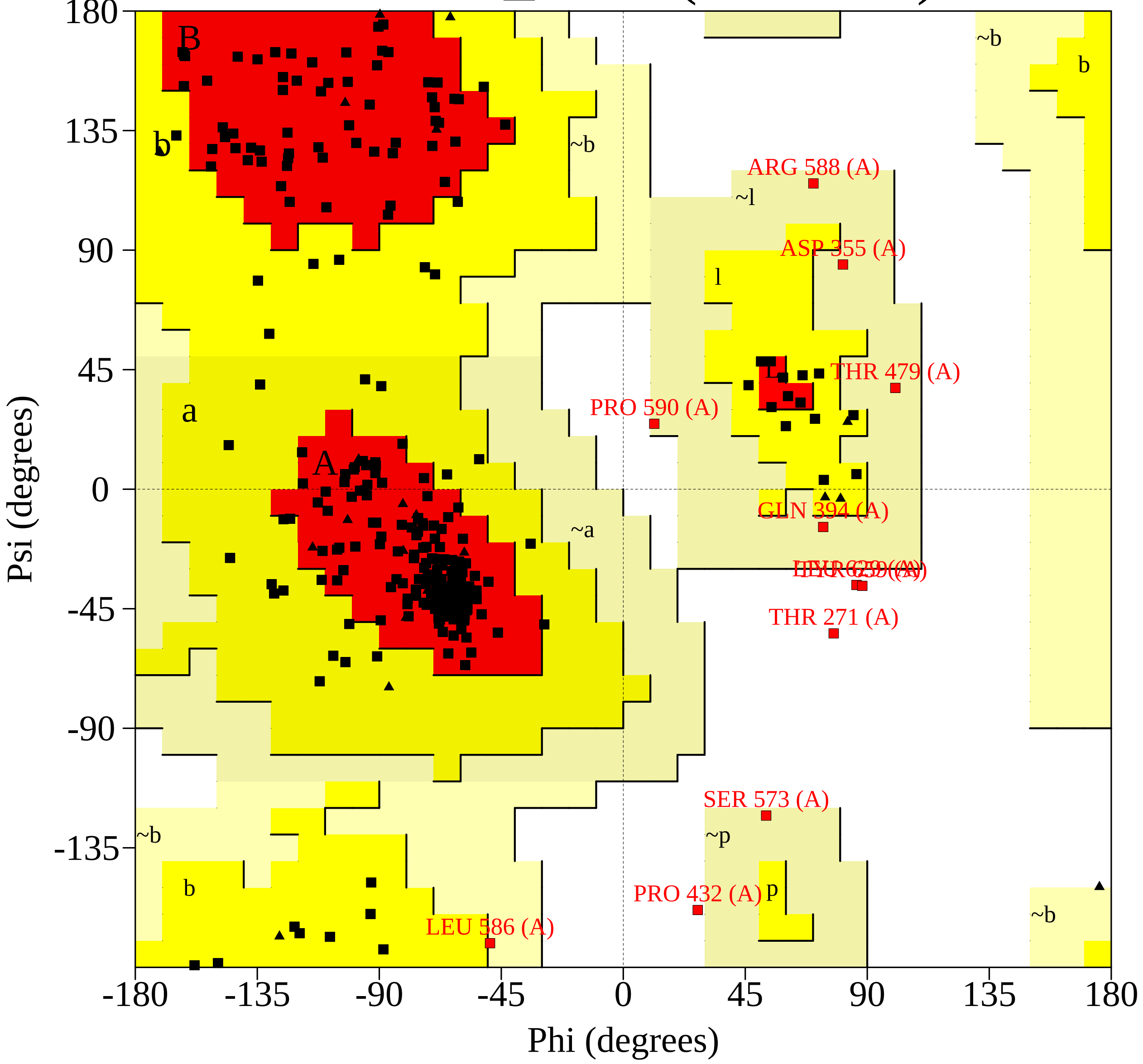

Plot statistics

Residues in most favoured regions [A,B,L]	301	85.5%
Residues in additional allowed regions [a,b,l,p]	42	11.9%
Residues in generously allowed regions [~a,~b,~l,~p]	6	1.7%
Residues in disallowed regions	3	0.9%
	----	------
Number of non-glycine and non-proline residues	352	100.0%
Number of end-residues (excl. Gly and Pro)	2	
Number of glycine residues (shown as triangles)	29	
Number of proline residues	19	

Total number of residues	402	

Based on an analysis of 118 structures of resolution of at least 2.0 Angstroms and R-factor no greater than 20%, a good quality model would be expected to have over 90% in the most favoured regions.

PROCHECK-NMR

Ramachandran Plot

SbHAK6_noHs (1 models)

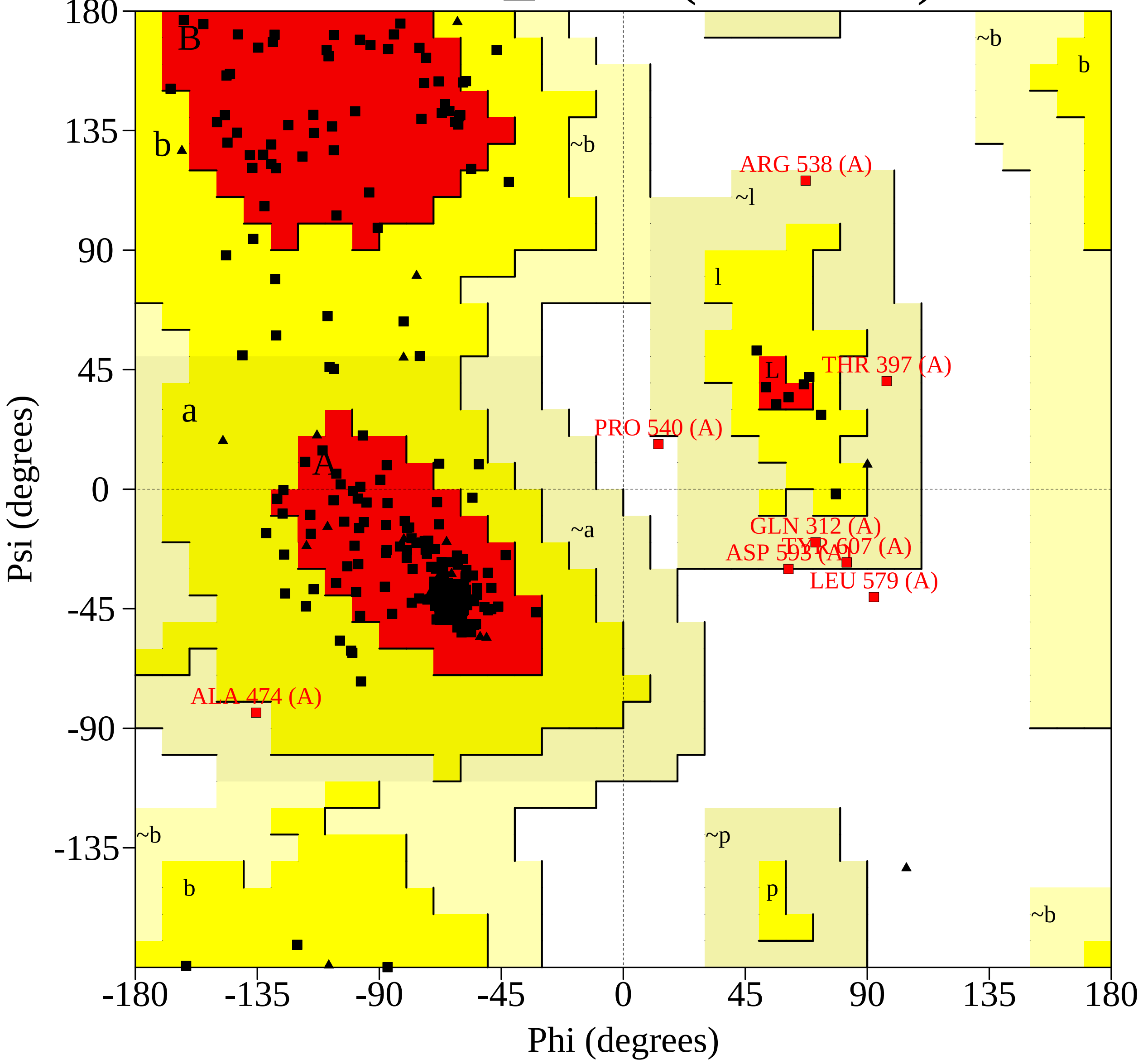

Plot statistics

Residues in most favoured regions [A,B,L]	250	87.1%
Residues in additional allowed regions [a,b,l,p]	30	10.5%
Residues in generously allowed regions [~a,~b,~l,~p]	5	1.7%
Residues in disallowed regions	2	0.7%
	----	------
Number of non-glycine and non-proline residues	287	100.0%
Number of end-residues (excl. Gly and Pro)	2	
Number of glycine residues (shown as triangles)	20	
Number of proline residues	15	

Total number of residues	324	

Based on an analysis of 118 structures of resolution of at least 2.0 Angstroms and R-factor no greater than 20%, a good quality model would be expected to have over 90% in the most favoured regions.

PROCHECK-NMR

Ramachandran Plot

SbHAK7_noHs (1 models)

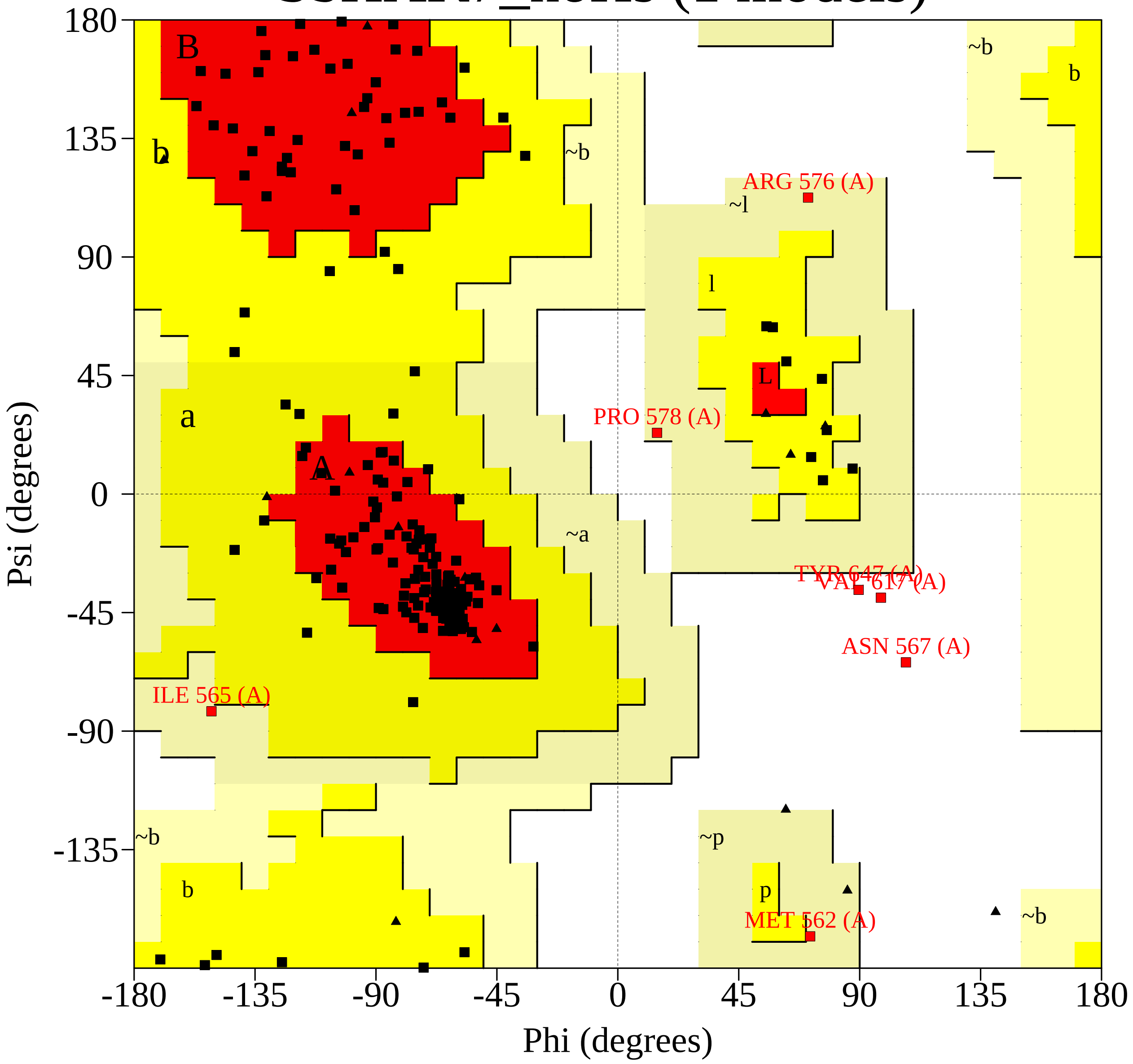

Plot statistics

Residues in most favoured regions [A,B,L]	199	85.0%
Residues in additional allowed regions [a,b,l,p]	29	12.4%
Residues in generously allowed regions [~a,~b,~l,~p]	3	1.3%
Residues in disallowed regions	3	1.3%
	----	------
Number of non-glycine and non-proline residues	234	100.0%
Number of end-residues (excl. Gly and Pro)	2	
Number of glycine residues (shown as triangles)	19	
Number of proline residues	13	

Total number of residues	268	

Based on an analysis of 118 structures of resolution of at least 2.0 Angstroms and R-factor no greater than 20%, a good quality model would be expected to have over 90% in the most favoured regions.

PROCHECK-NMR

Ramachandran Plot

SbHAK8_noHs (1 models)

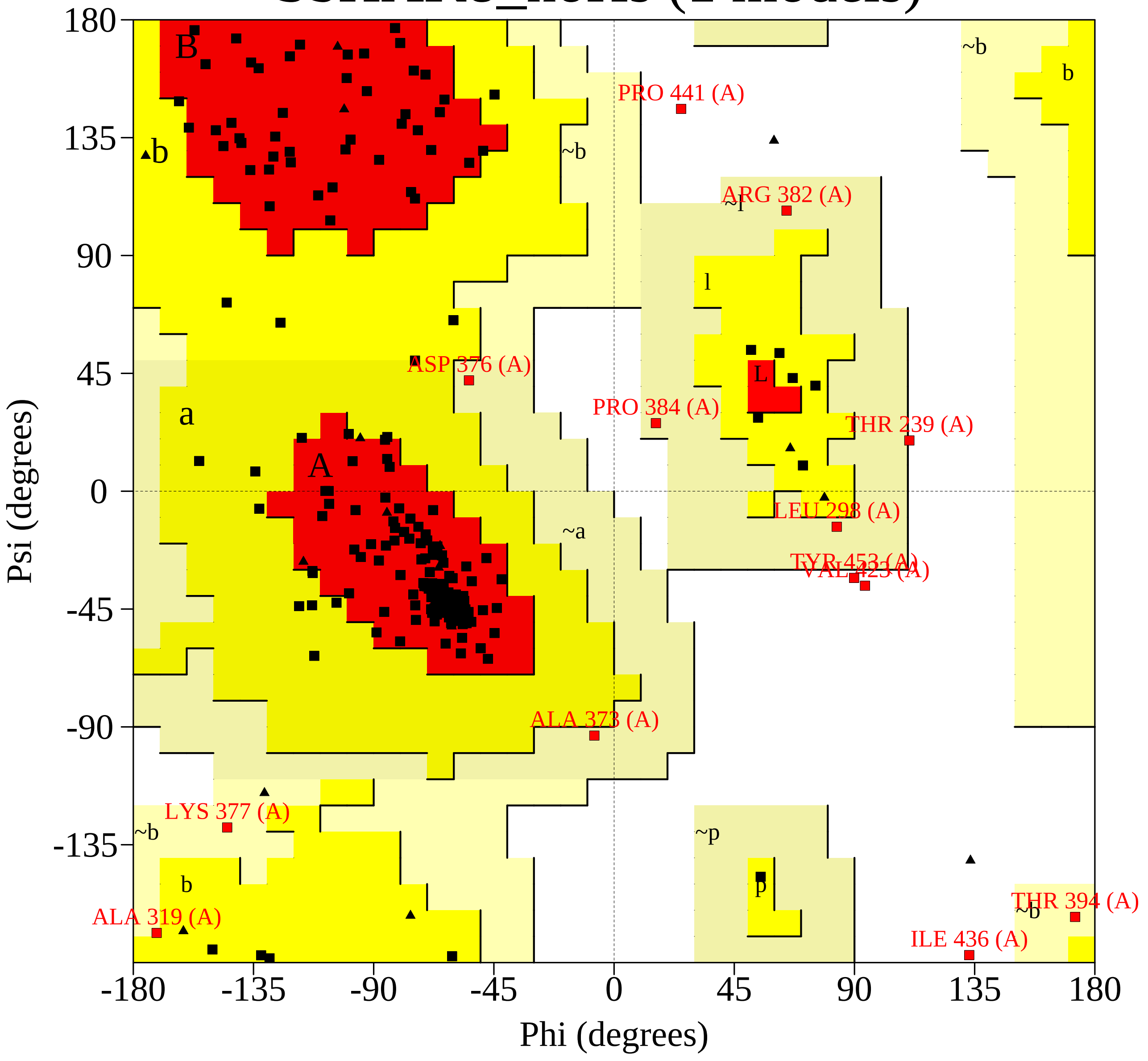

Plot statistics

Residues in most favoured regions [A,B,L]	194	84.0%
Residues in additional allowed regions [a,b,l,p]	26	11.3%
Residues in generously allowed regions [~a,~b,~l,~p]	7	3.0%
Residues in disallowed regions	4	1.7%
	----	------
Number of non-glycine and non-proline residues	231	100.0%
Number of end-residues (excl. Gly and Pro)	2	
Number of glycine residues (shown as triangles)	17	
Number of proline residues	14	

Total number of residues	264	

Based on an analysis of 118 structures of resolution of at least 2.0 Angstroms and R-factor no greater than 20%, a good quality model would be expected to have over 90% in the most favoured regions.

SbHAK8_noHs_01_ramachand.ps

PROCHECK-NMR

Ramachandran Plot

SbHAK9_noHs (1 models)

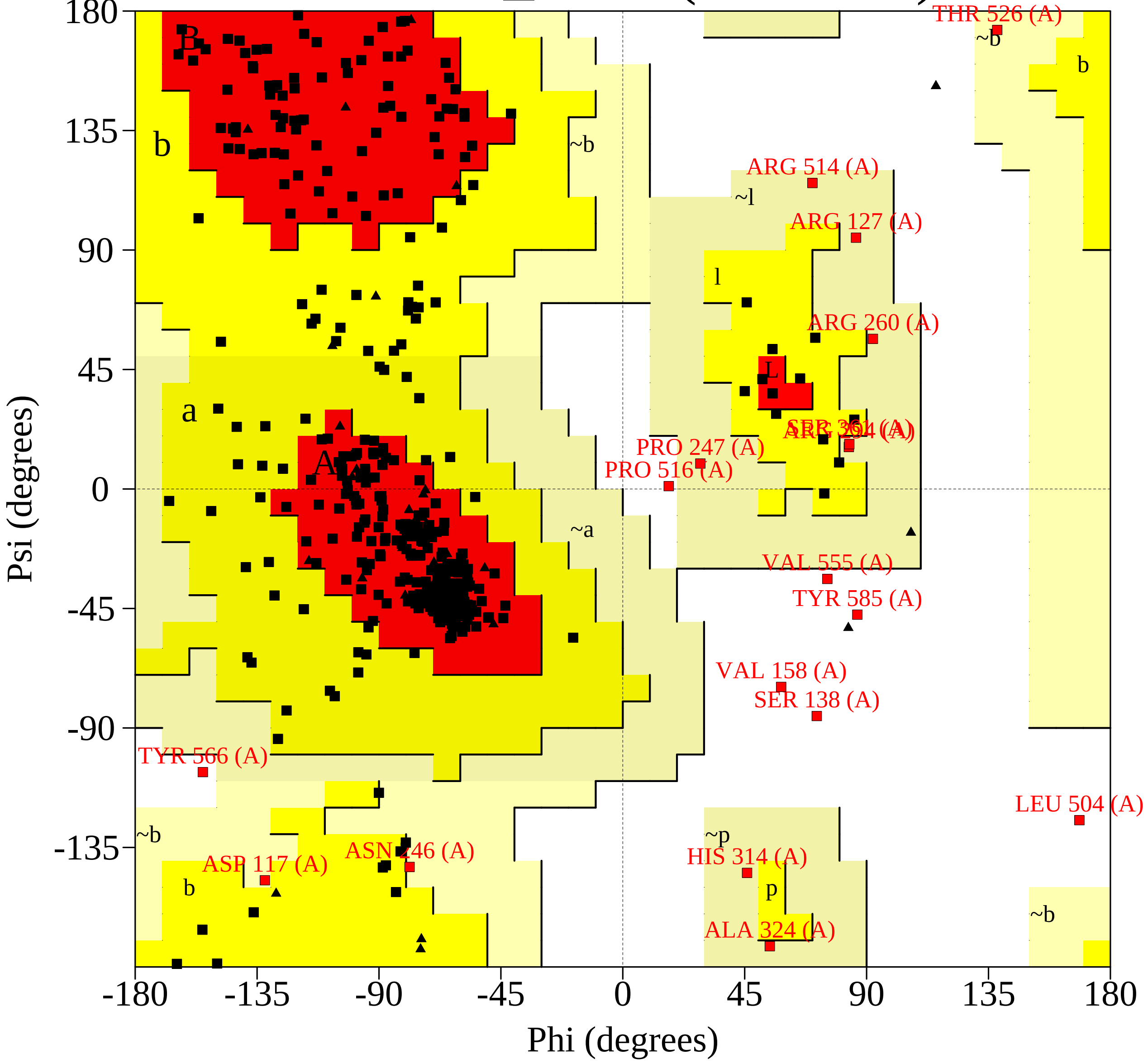

Plot statistics

Residues in most favoured regions [A,B,L]	426	82.9%
Residues in additional allowed regions [a,b,l,p]	72	14.0%
Residues in generously allowed regions [~a,~b,~l,~p]	10	1.9%
Residues in disallowed regions	6	1.2%
	----	------
Number of non-glycine and non-proline residues	514	100.0%
Number of end-residues (excl. Gly and Pro)	2	
Number of glycine residues (shown as triangles)	36	
Number of proline residues	23	

Total number of residues	575	

Based on an analysis of 118 structures of resolution of at least 2.0 Angstroms and R-factor no greater than 20%, a good quality model would be expected to have over 90% in the most favoured regions.

SbHAK9_noHs_01_ramachand.ps

PROCHECK-NMR

Ramachandran Plot

SbHAK10_noHs (1 models)

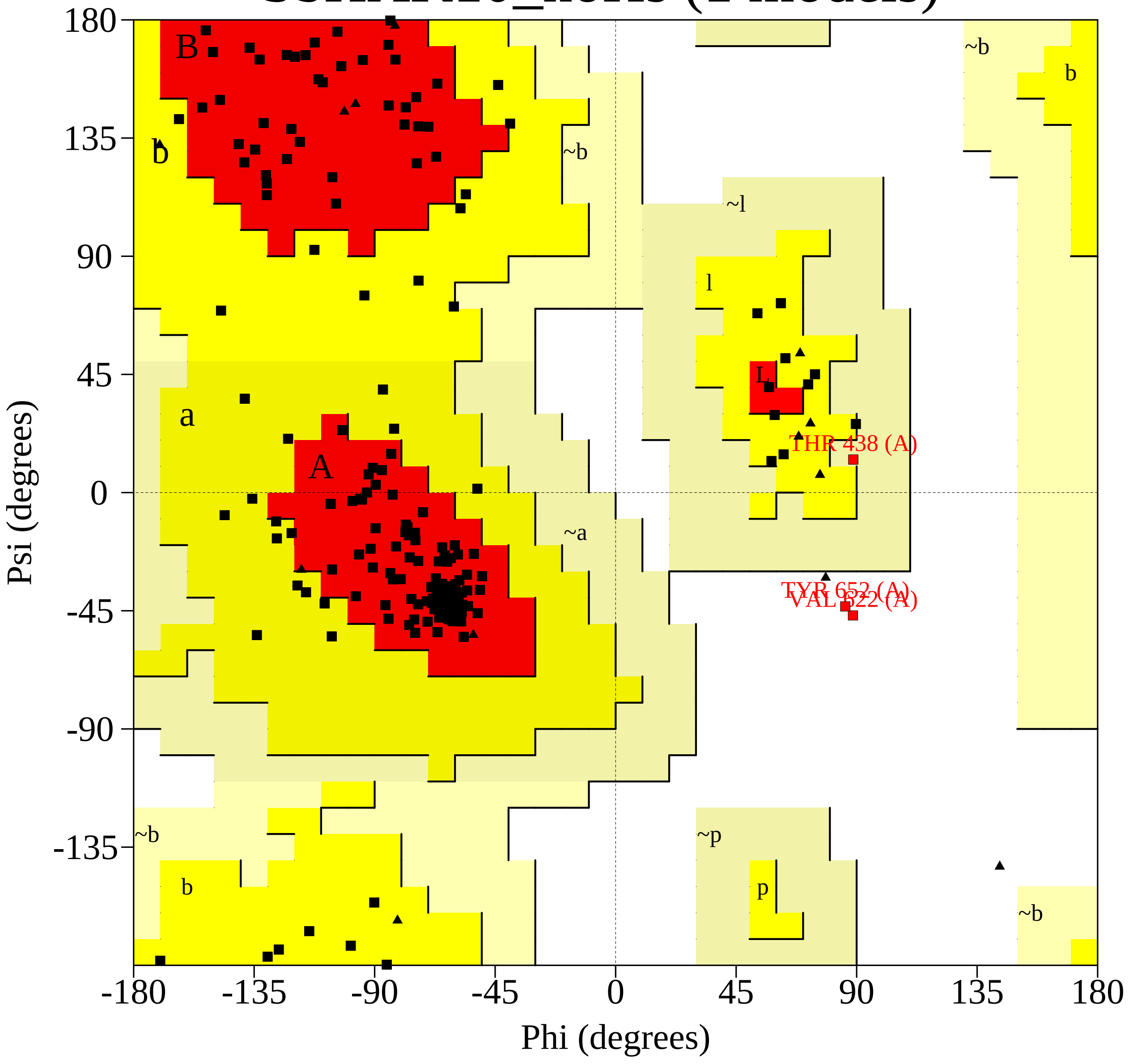

Plot statistics

Residues in most favoured regions [A,B,L]	168	81.2%
Residues in additional allowed regions [a,b,l,p]	36	17.4%
Residues in generously allowed regions [~a,~b,~l,~p]	1	0.5%
Residues in disallowed regions	2	1.0%
	----	------
Number of non-glycine and non-proline residues	207	100.0%
Number of end-residues (excl. Gly and Pro)	2	
Number of glycine residues (shown as triangles)	16	
Number of proline residues	10	

Total number of residues	235	

Based on an analysis of 118 structures of resolution of at least 2.0 Angstroms and R-factor no greater than 20%, a good quality model would be expected to have over 90% in the most favoured regions.

PROCHECK-NMR

Ramachandran Plot

SbHAK11_noHs (1 models)

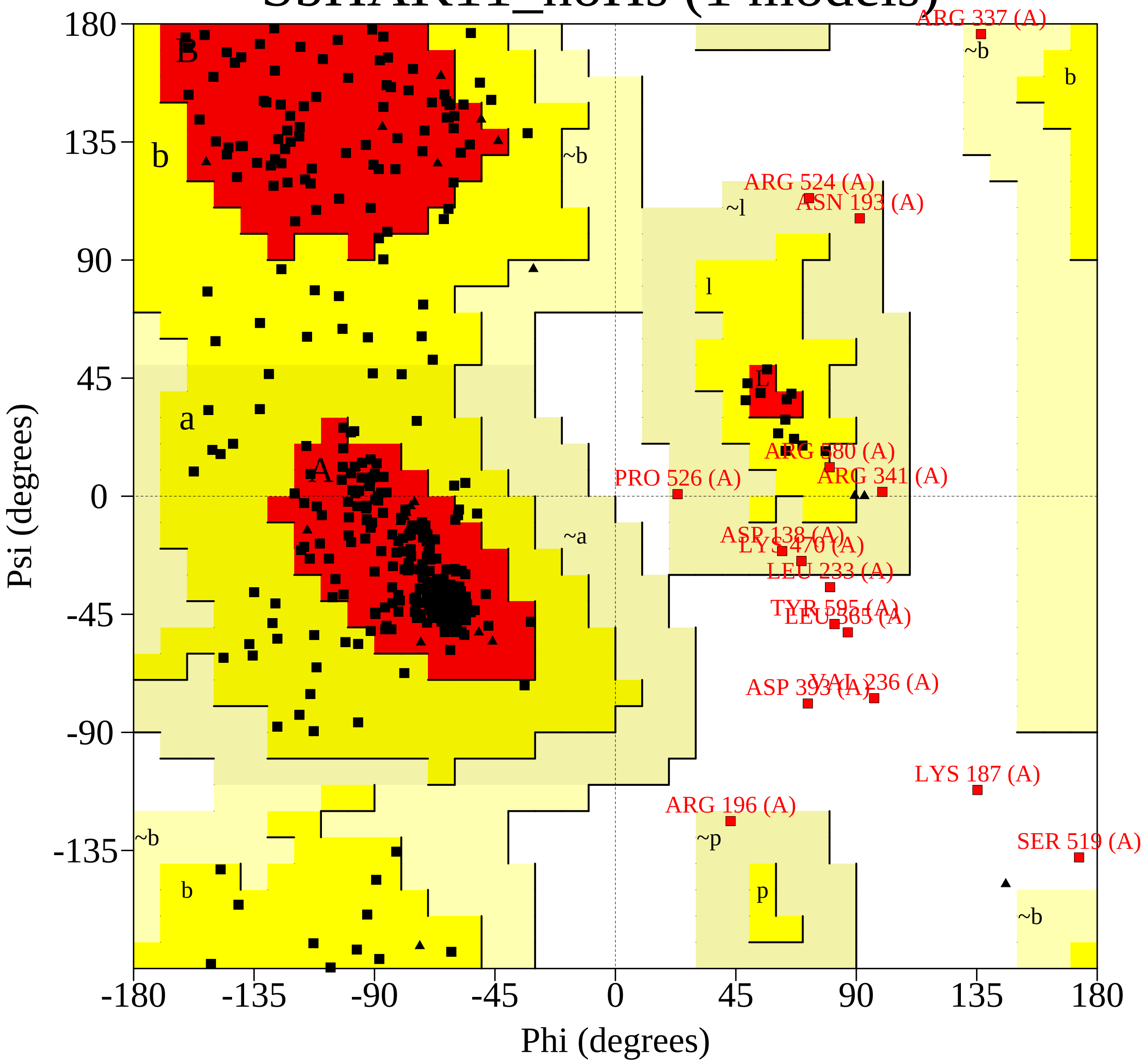

Plot statistics

Residues in most favoured regions [A,B,L]	357	80.6%
Residues in additional allowed regions [a,b,l,p]	71	16.0%
Residues in generously allowed regions [~a,~b,~l,~p]	8	1.8%
Residues in disallowed regions	7	1.6%
	----	------
Number of non-glycine and non-proline residues	443	100.0%
Number of end-residues (excl. Gly and Pro)	2	
Number of glycine residues (shown as triangles)	37	
Number of proline residues	19	

Total number of residues	501	

Based on an analysis of 118 structures of resolution of at least 2.0 Angstroms and R-factor no greater than 20%, a good quality model would be expected to have over 90% in the most favoured regions.

PROCHECK-NMR

Ramachandran Plot

SbHAK12_noHs (1 models)

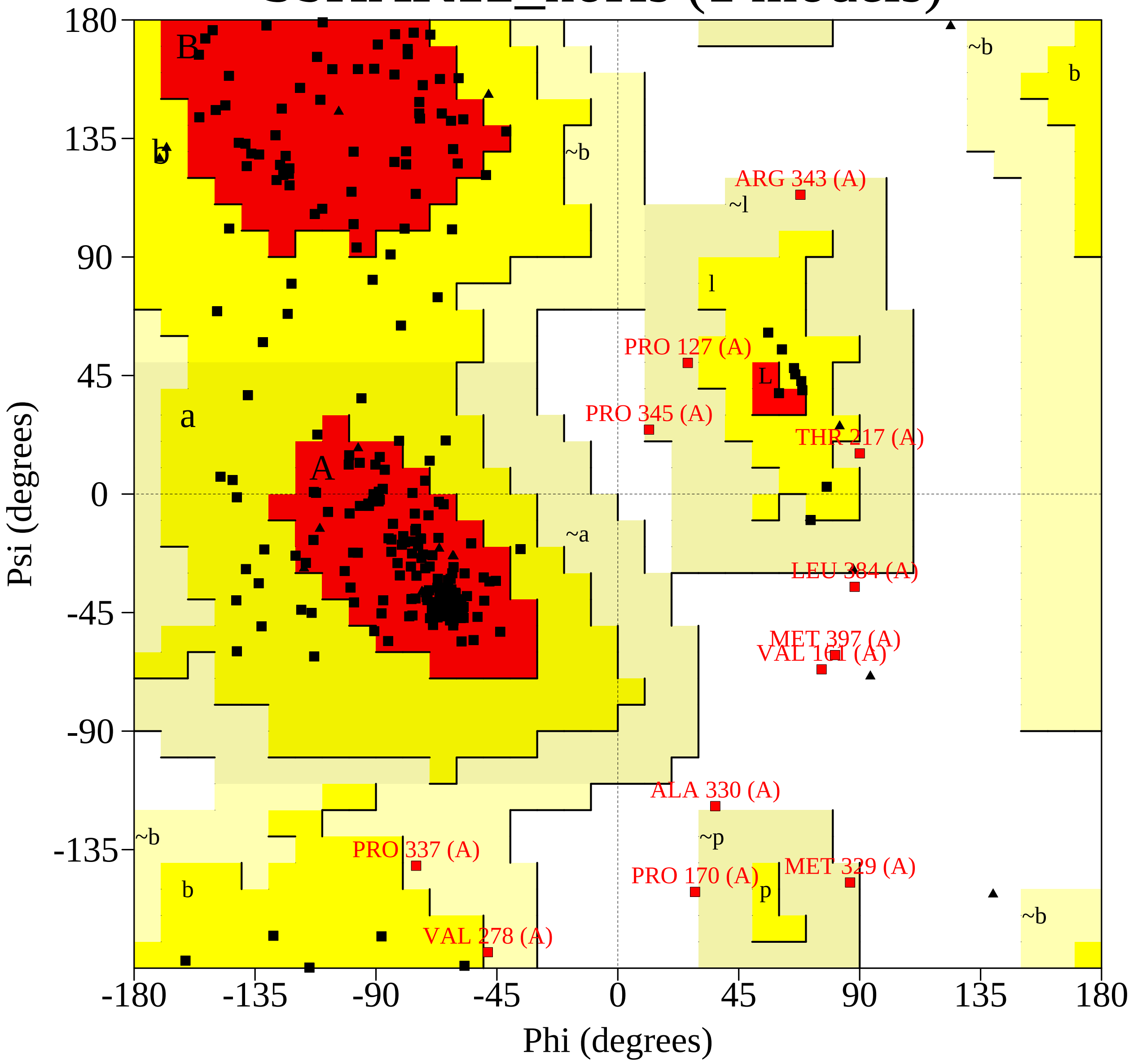

Plot statistics

Residues in most favoured regions [A,B,L]	231	82.2%
Residues in additional allowed regions [a,b,l,p]	42	14.9%
Residues in generously allowed regions [~a,~b,~l,~p]	4	1.4%
Residues in disallowed regions	4	1.4%
	----	------
Number of non-glycine and non-proline residues	281	100.0%
Number of end-residues (excl. Gly and Pro)	2	
Number of glycine residues (shown as triangles)	16	
Number of proline residues	15	

Total number of residues	314	

Based on an analysis of 118 structures of resolution of at least 2.0 Angstroms and R-factor no greater than 20%, a good quality model would be expected to have over 90% in the most favoured regions.

SbHAK12_noHs_01_ramachand.ps

PROCHECK-NMR

Ramachandran Plot

SbHAK13_noHs (1 models)

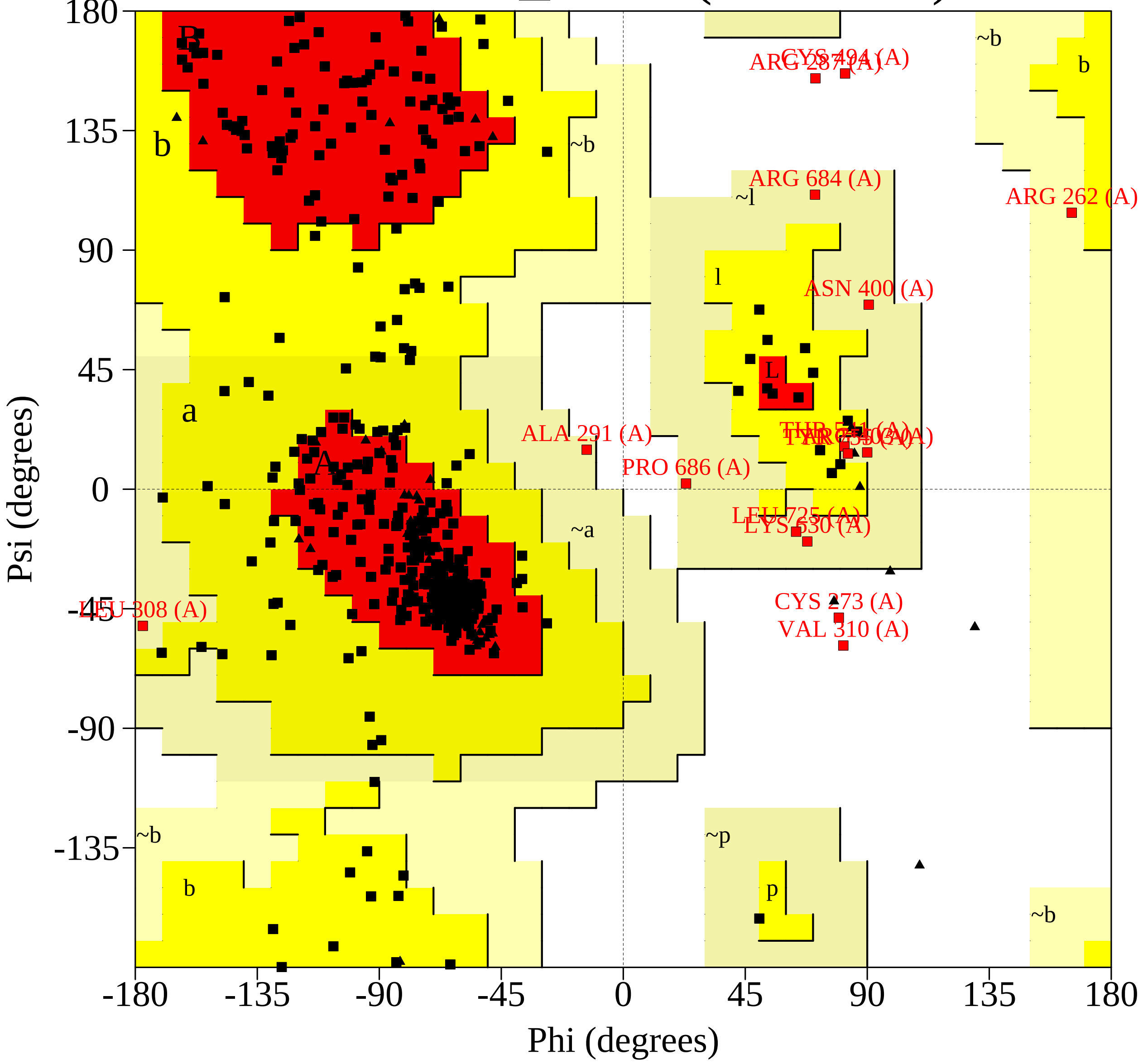

Plot statistics

Residues in most favoured regions [A,B,L]	408	81.0%
Residues in additional allowed regions [a,b,l,p]	82	16.3%
Residues in generously allowed regions [~a,~b,~l,~p]	10	2.0%
Residues in disallowed regions	4	0.8%
	----	------
Number of non-glycine and non-proline residues	504	100.0%
Number of end-residues (excl. Gly and Pro)	2	
Number of glycine residues (shown as triangles)	54	
Number of proline residues	23	

Total number of residues	583	

Based on an analysis of 118 structures of resolution of at least 2.0 Angstroms and R-factor no greater than 20%, a good quality model would be expected to have over 90% in the most favoured regions.

PROCHECK-NMR

Ramachandran Plot

SbHAK14_noHs (1 models)

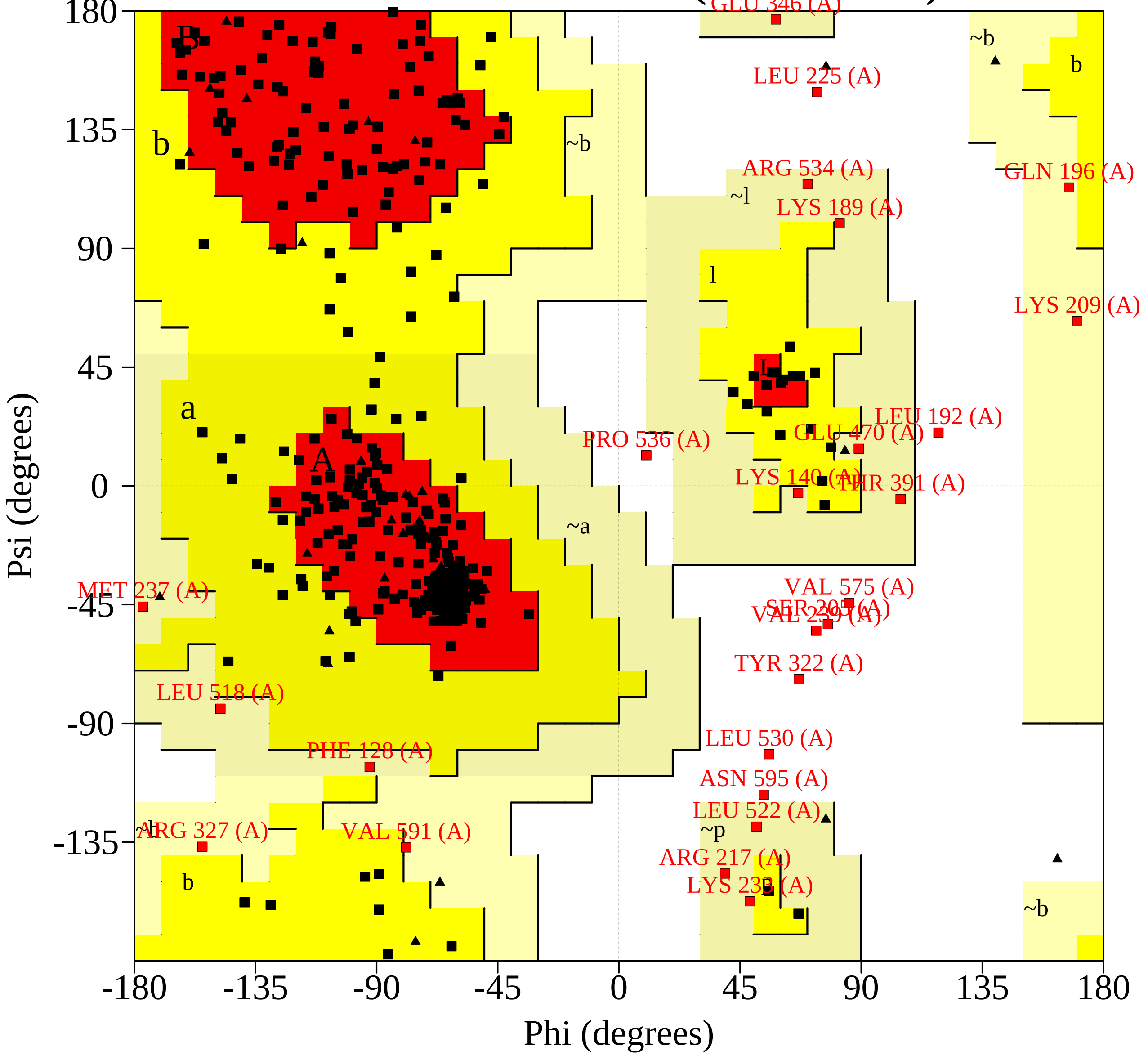

Plot statistics

Residues in most favoured regions [A,B,L]	363	81.4%
Residues in additional allowed regions [a,b,l,p]	59	13.2%
Residues in generously allowed regions [~a,~b,~l,~p]	16	3.6%
Residues in disallowed regions	8	1.8%
	----	------
Number of non-glycine and non-proline residues	446	100.0%
Number of end-residues (excl. Gly and Pro)	2	
Number of glycine residues (shown as triangles)	37	
Number of proline residues	19	

Total number of residues	504	

Based on an analysis of 118 structures of resolution of at least 2.0 Angstroms and R-factor no greater than 20%, a good quality model would be expected to have over 90% in the most favoured regions.

SbHAK14_noHs_01_ramachand.ps

PROCHECK-NMR

Ramachandran Plot

SbHAK15_noHs (1 models)

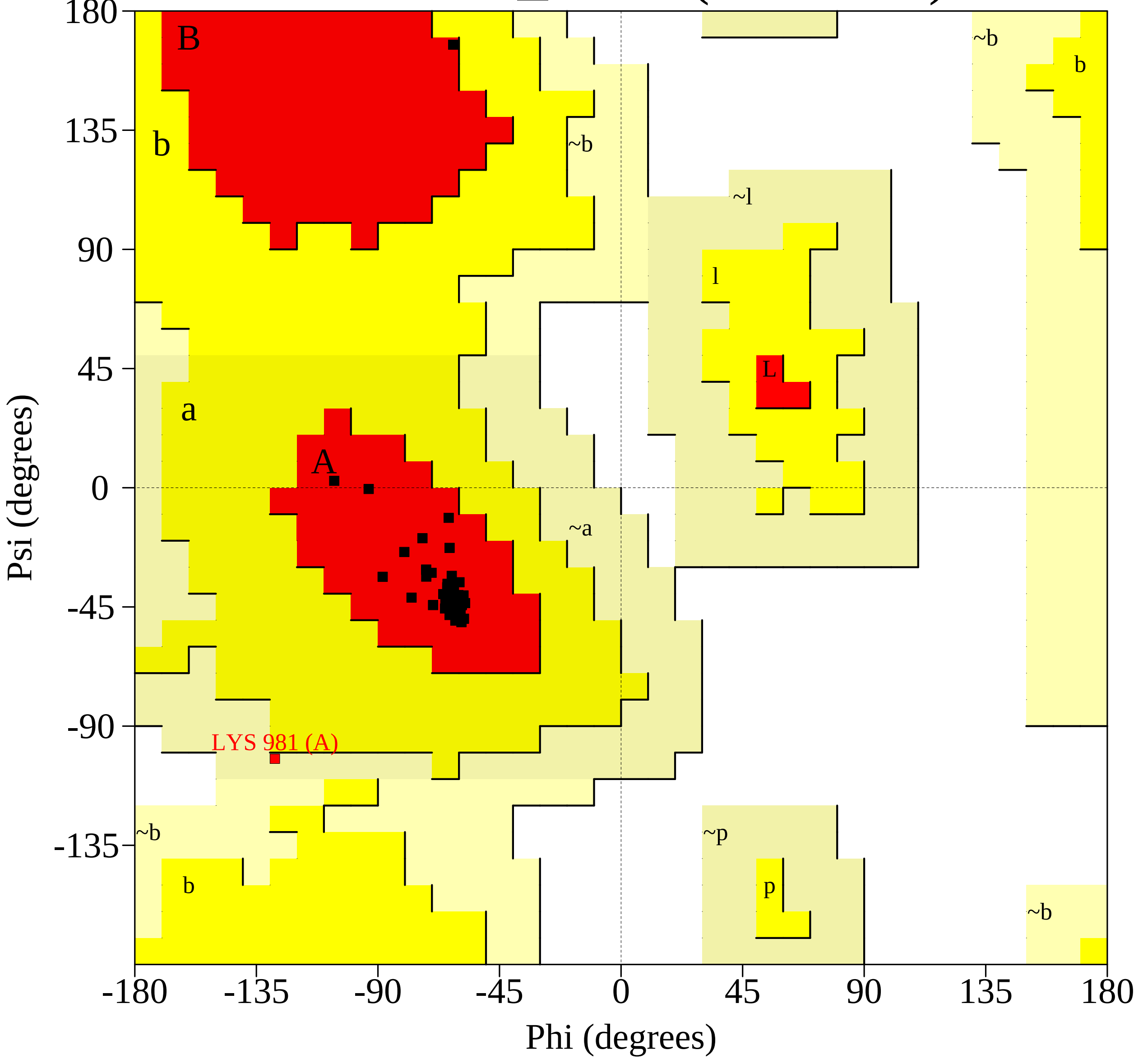

Plot statistics

Residues in most favoured regions [A,B,L]	54	98.2%
Residues in additional allowed regions [a,b,l,p]	0	0.0%
Residues in generously allowed regions [~a,~b,~l,~p]	1	1.8%
Residues in disallowed regions	0	0.0%
	----	------
Number of non-glycine and non-proline residues	55	100.0%
Number of end-residues (excl. Gly and Pro)	2	
Number of glycine residues (shown as triangles)	1	
Number of proline residues	0	

Total number of residues	58	

Based on an analysis of 118 structures of resolution of at least 2.0 Angstroms and R-factor no greater than 20%, a good quality model would be expected to have over 90% in the most favoured regions.

PROCHECK-NMR

Ramachandran Plot

SbHAK16_noHs (1 models)

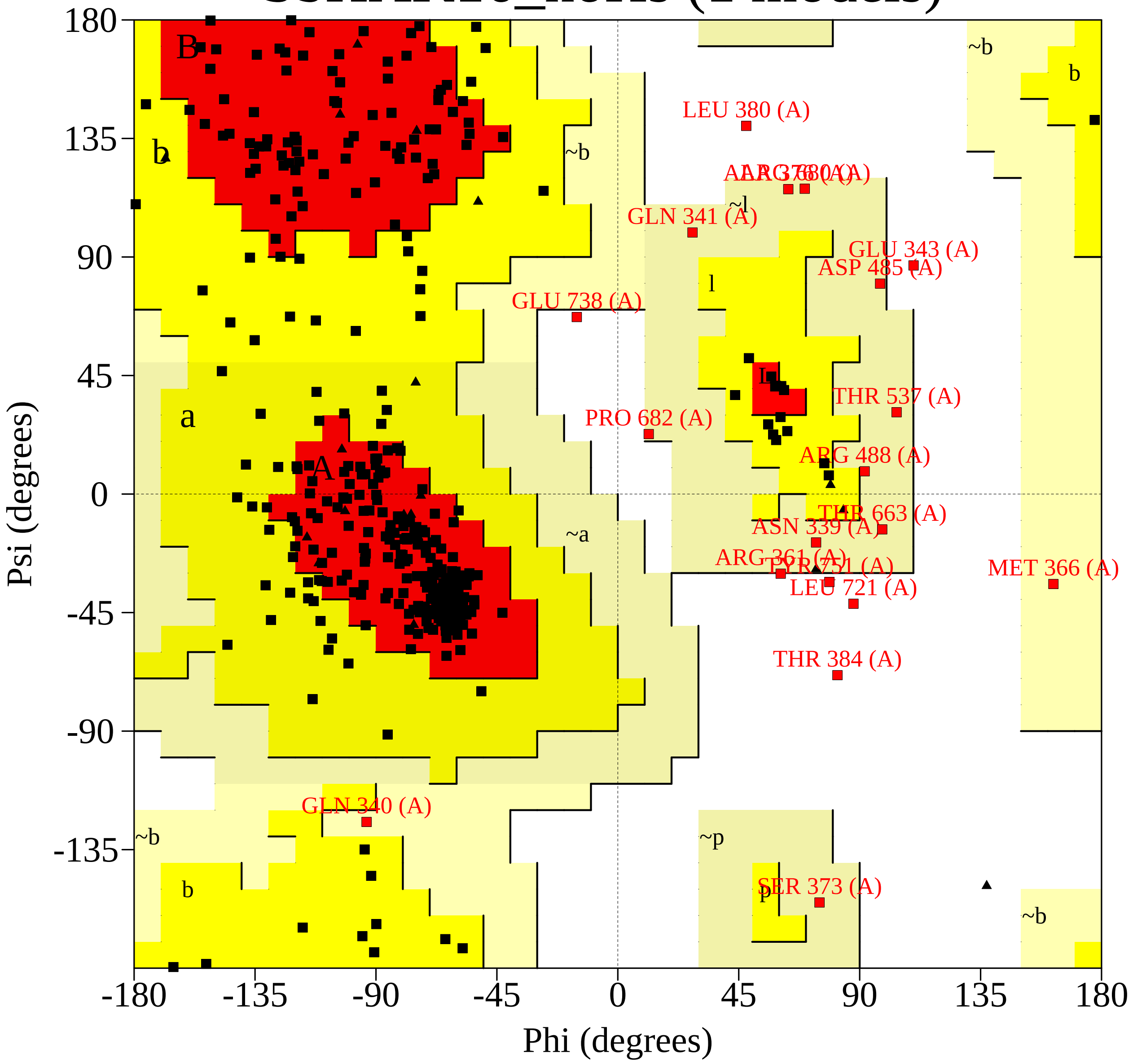

Plot statistics

Residues in most favoured regions [A,B,L]	378	80.9%
Residues in additional allowed regions [a,b,l,p]	71	15.2%
Residues in generously allowed regions [~a,~b,~l,~p]	11	2.4%
Residues in disallowed regions	7	1.5%
	----	------
Number of non-glycine and non-proline residues	467	100.0%
Number of end-residues (excl. Gly and Pro)	2	
Number of glycine residues (shown as triangles)	25	
Number of proline residues	18	

Total number of residues	512	

Based on an analysis of 118 structures of resolution of at least 2.0 Angstroms and R-factor no greater than 20%, a good quality model would be expected to have over 90% in the most favoured regions.

PROCHECK-NMR

Ramachandran Plot

SbHAK17_noHs (1 models)

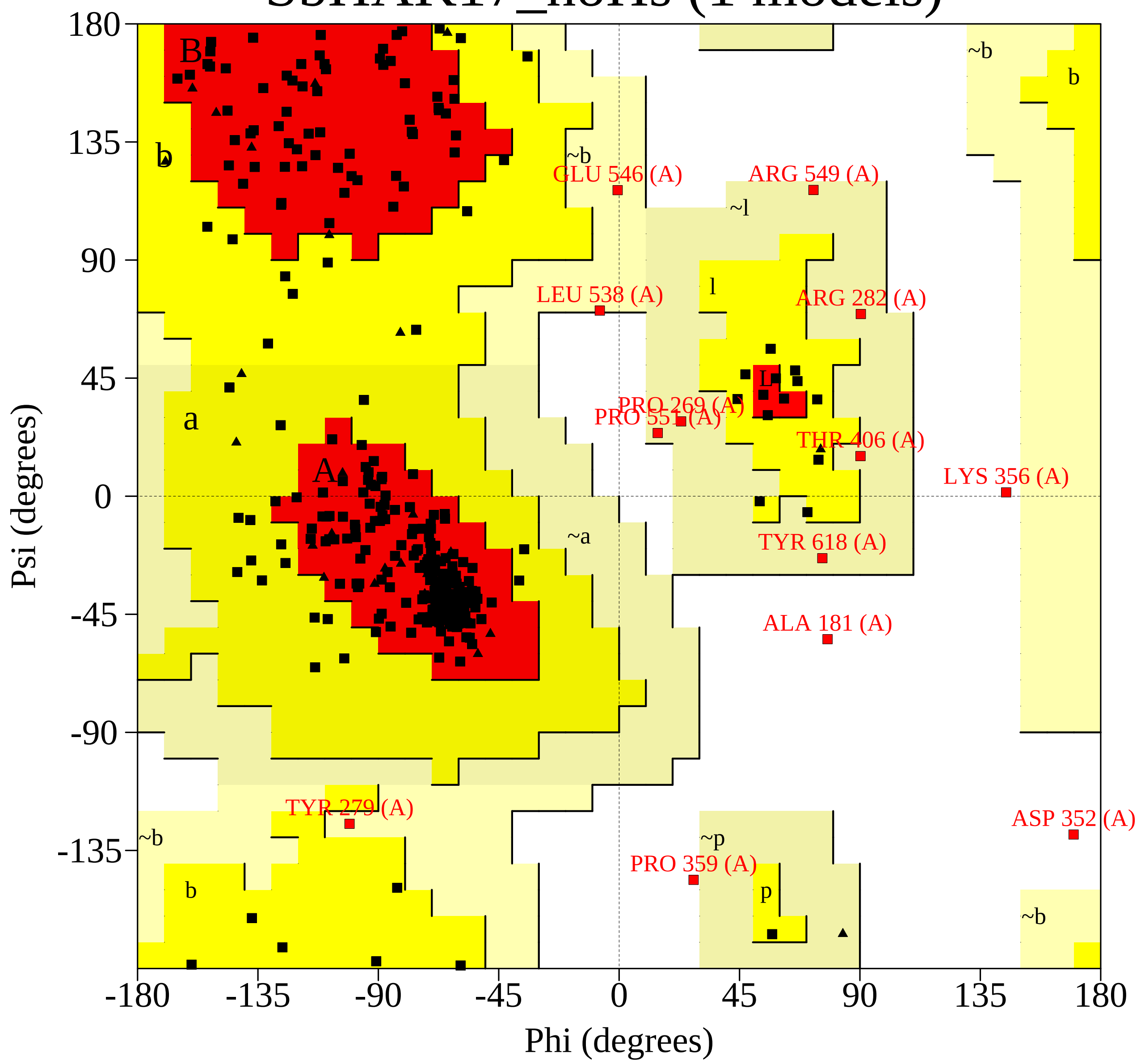

Plot statistics

Residues in most favoured regions [A,B,L]	336	86.6%
Residues in additional allowed regions [a,b,l,p]	42	10.8%
Residues in generously allowed regions [~a,~b,~l,~p]	7	1.8%
Residues in disallowed regions	3	0.8%
	----	------
Number of non-glycine and non-proline residues	388	100.0%
Number of end-residues (excl. Gly and Pro)	2	
Number of glycine residues (shown as triangles)	35	
Number of proline residues	22	

Total number of residues	447	

Based on an analysis of 118 structures of resolution of at least 2.0 Angstroms and R-factor no greater than 20%, a good quality model would be expected to have over 90% in the most favoured regions.

PROCHECK-NMR

Ramachandran Plot

SbHAK18_noHs (1 models)

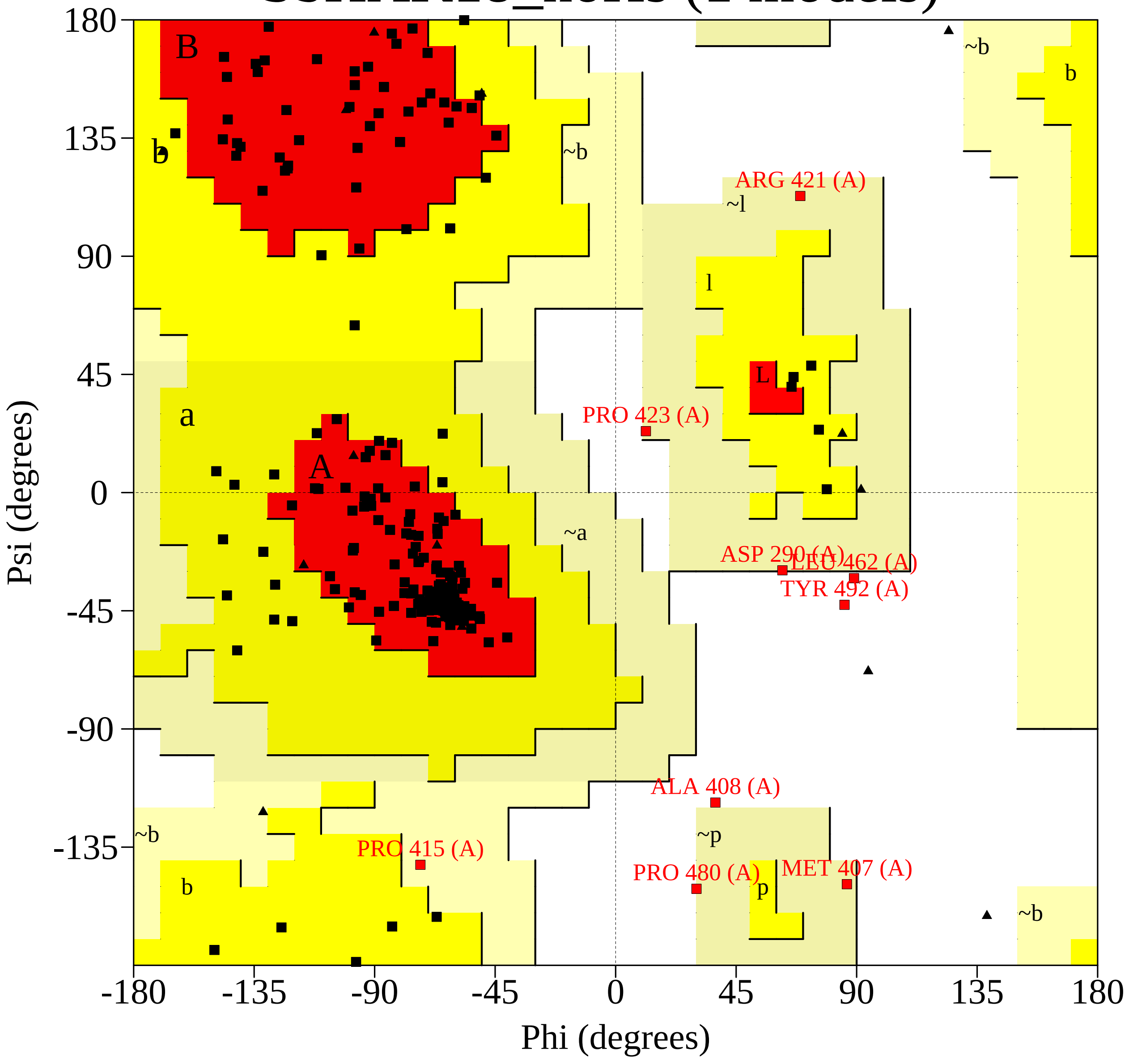

Plot statistics

Residues in most favoured regions [A,B,L]	212	86.5%
Residues in additional allowed regions [a,b,l,p]	27	11.0%
Residues in generously allowed regions [~a,~b,~l,~p]	3	1.2%
Residues in disallowed regions	3	1.2%
	----	------
Number of non-glycine and non-proline residues	245	100.0%
Number of end-residues (excl. Gly and Pro)	2	
Number of glycine residues (shown as triangles)	15	
Number of proline residues	13	

Total number of residues	275	

Based on an analysis of 118 structures of resolution of at least 2.0 Angstroms and R-factor no greater than 20%, a good quality model would be expected to have over 90% in the most favoured regions.

SbHAK18_noHs_01_ramachand.ps

PROCHECK-NMR

Ramachandran Plot

SbHAK19_noHs (1 models)

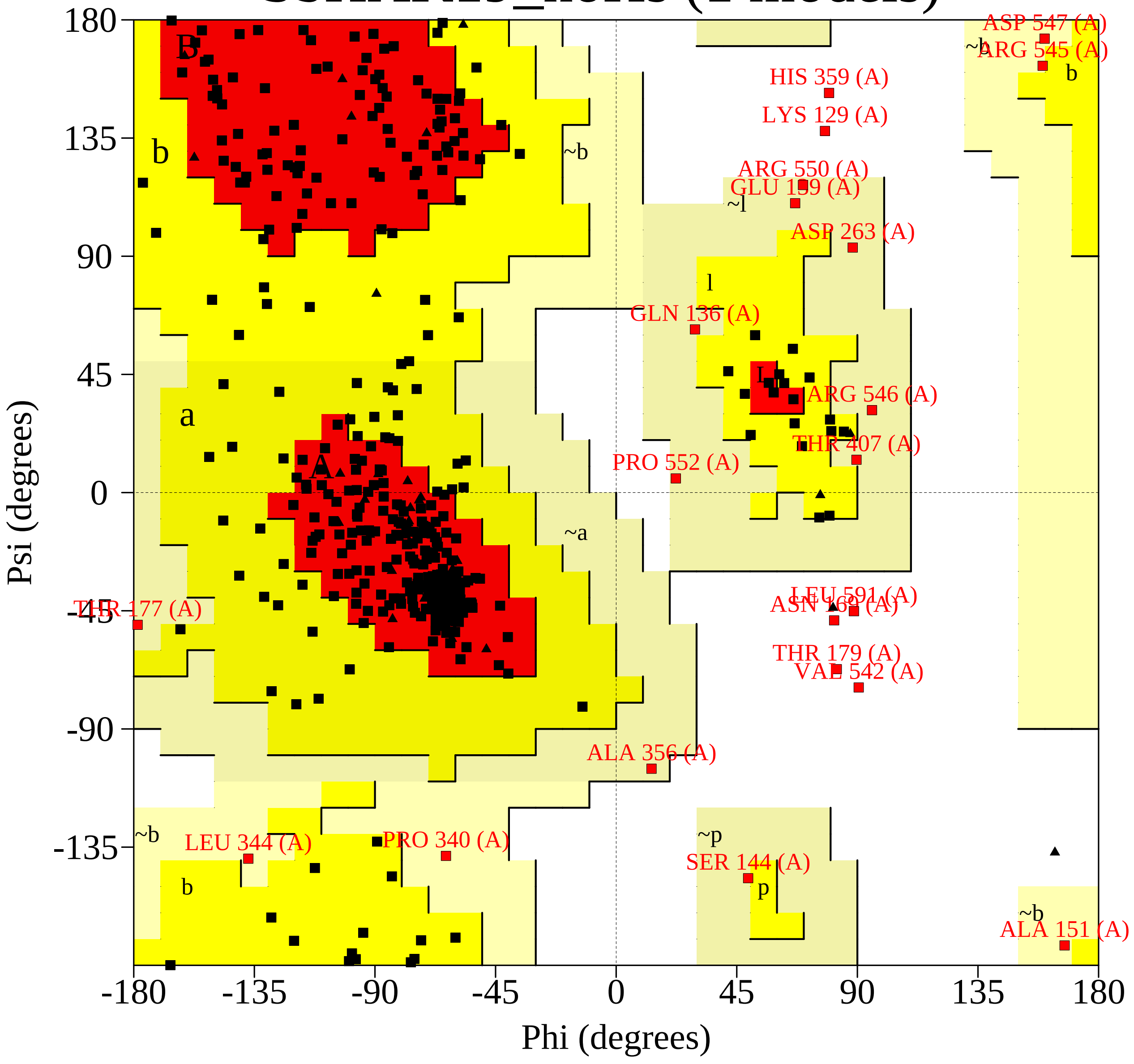

Plot statistics

Residues in most favoured regions [A,B,L]	417	81.0%
Residues in additional allowed regions [a,b,l,p]	79	15.3%
Residues in generously allowed regions [~a,~b,~l,~p]	13	2.5%
Residues in disallowed regions	6	1.2%
	----	------
Number of non-glycine and non-proline residues	515	100.0%
Number of end-residues (excl. Gly and Pro)	2	
Number of glycine residues (shown as triangles)	38	
Number of proline residues	27	

Total number of residues	582	

Based on an analysis of 118 structures of resolution of at least 2.0 Angstroms and R-factor no greater than 20%, a good quality model would be expected to have over 90% in the most favoured regions.

SbHAK19_noHs_01_ramachand.ps

PROCHECK-NMR

Ramachandran Plot

SbHAK20_noHs (1 models)

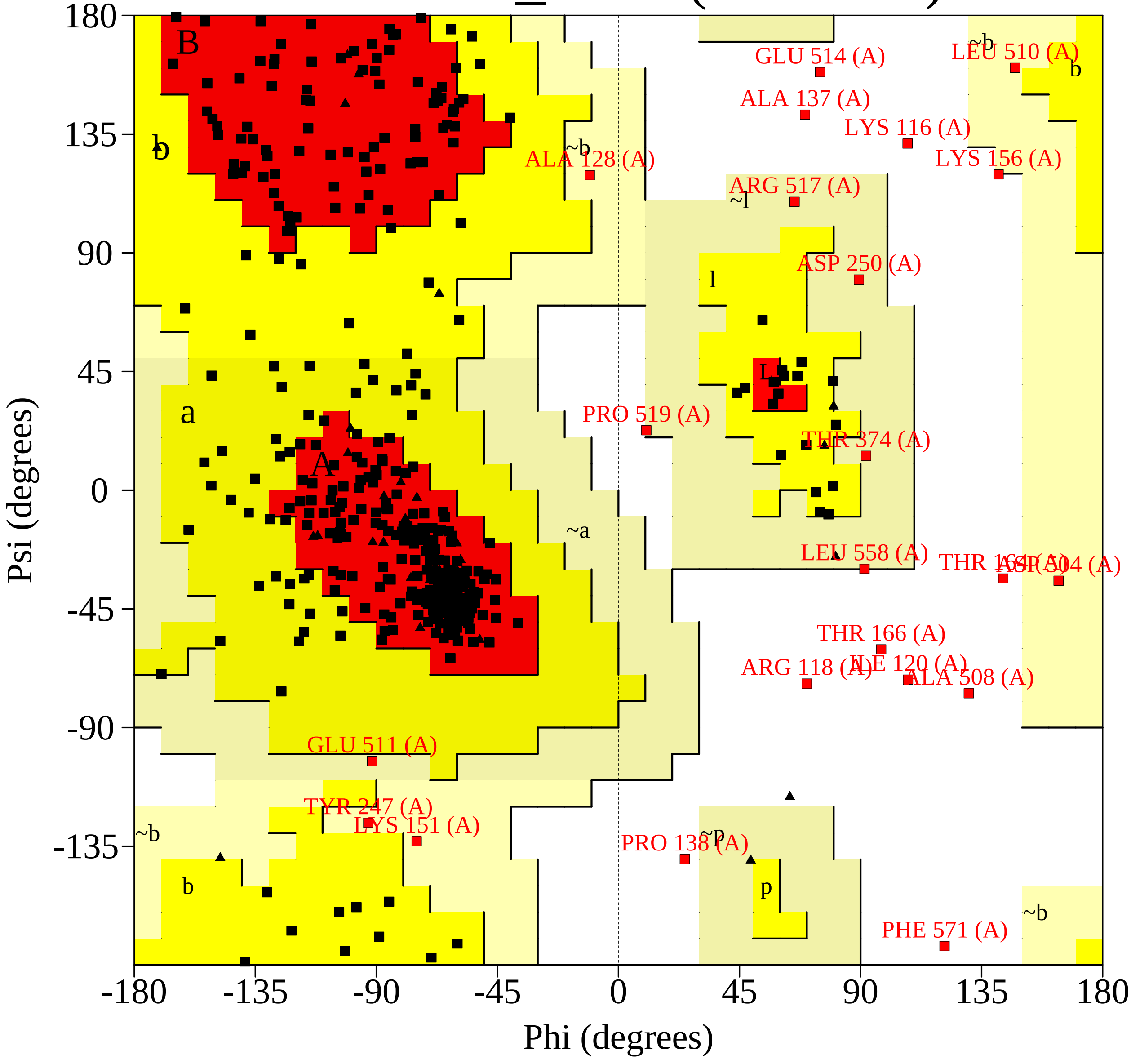

Plot statistics

Residues in most favoured regions [A,B,L]	401	81.0%
Residues in additional allowed regions [a,b,l,p]	74	14.9%
Residues in generously allowed regions [~a,~b,~l,~p]	10	2.0%
Residues in disallowed regions	10	2.0%
	----	------
Number of non-glycine and non-proline residues	495	100.0%
Number of end-residues (excl. Gly and Pro)	2	
Number of glycine residues (shown as triangles)	37	
Number of proline residues	26	

Total number of residues	560	

Based on an analysis of 118 structures of resolution of at least 2.0 Angstroms and R-factor no greater than 20%, a good quality model would be expected to have over 90% in the most favoured regions.

PROCHECK-NMR

Ramachandran Plot

SbHAK21_noHs (1 models)

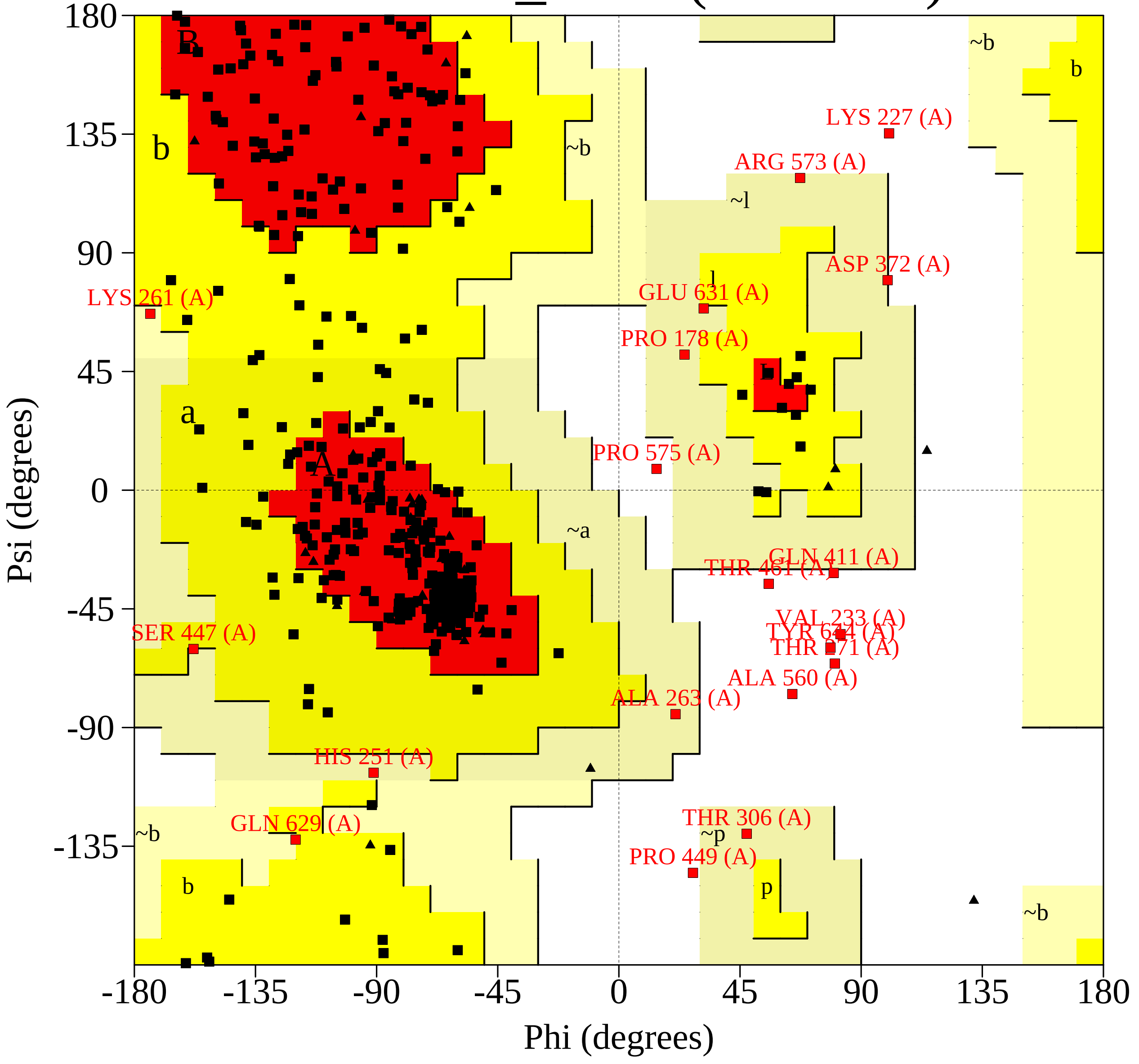

Plot statistics

Residues in most favoured regions [A,B,L]	365	81.1%
Residues in additional allowed regions [a,b,l,p]	69	15.3%
Residues in generously allowed regions [~a,~b,~l,~p]	9	2.0%
Residues in disallowed regions	7	1.6%
	----	------
Number of non-glycine and non-proline residues	450	100.0%
Number of end-residues (excl. Gly and Pro)	2	
Number of glycine residues (shown as triangles)	35	
Number of proline residues	22	

Total number of residues	509	

Based on an analysis of 118 structures of resolution of at least 2.0 Angstroms and R-factor no greater than 20%, a good quality model would be expected to have over 90% in the most favoured regions.

PROCHECK-NMR

Ramachandran Plot

SbHAK22_noHs (1 models)

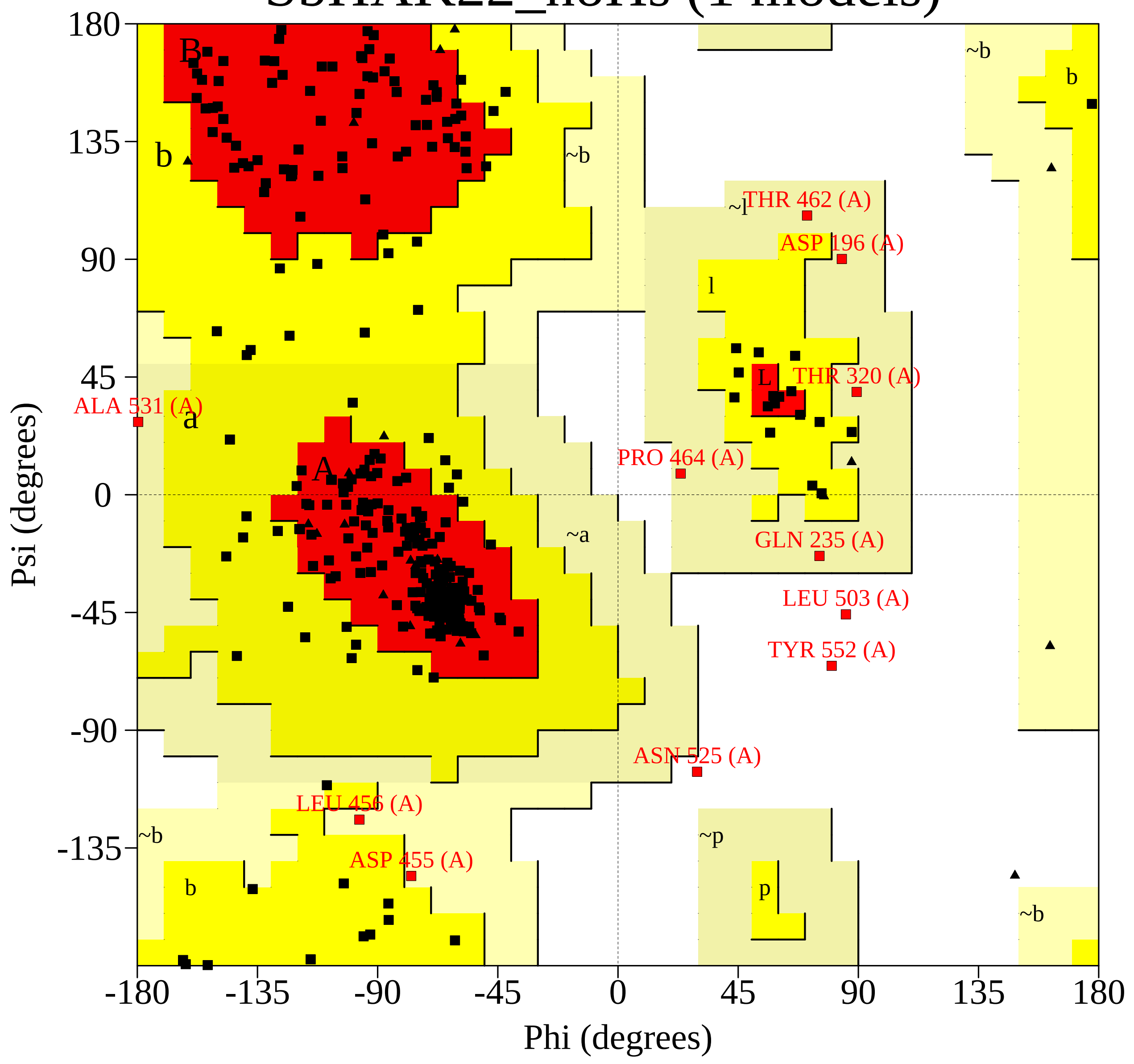

Plot statistics

Residues in most favoured regions [A,B,L]	338	84.3%
Residues in additional allowed regions [a,b,l,p]	53	13.2%
Residues in generously allowed regions [~a,~b,~l,~p]	7	1.7%
Residues in disallowed regions	3	0.7%
	----	------
Number of non-glycine and non-proline residues	401	100.0%
Number of end-residues (excl. Gly and Pro)	2	
Number of glycine residues (shown as triangles)	33	
Number of proline residues	22	

Total number of residues	458	

Based on an analysis of 118 structures of resolution of at least 2.0 Angstroms and R-factor no greater than 20%, a good quality model would be expected to have over 90% in the most favoured regions.

SbHAK22_noHs_01_ramachand.ps

PROCHECK-NMR

Ramachandran Plot

SbHAK23_noHs (1 models)

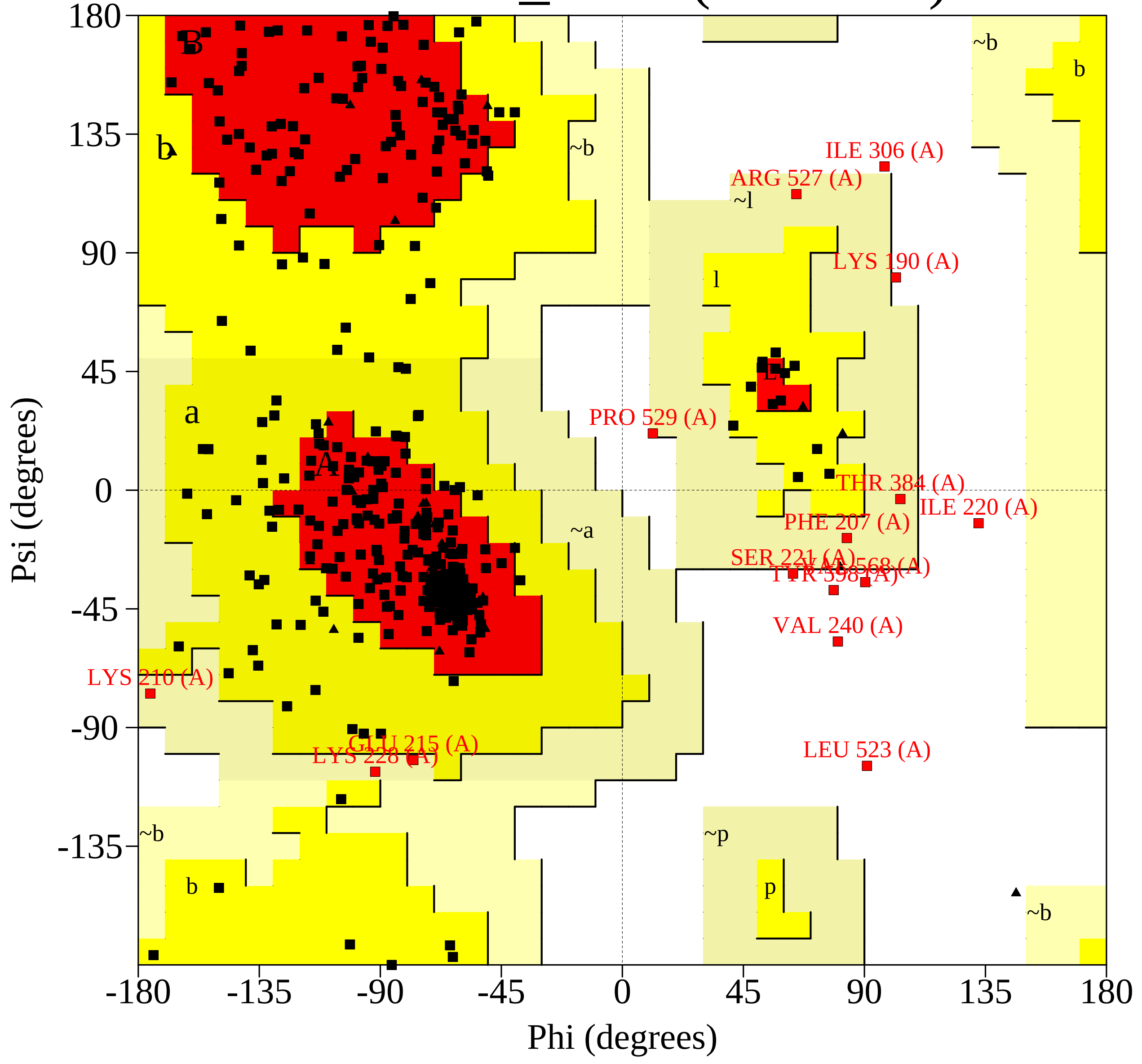

Plot statistics

Residues in most favoured regions [A,B,L]	352	80.0%
Residues in additional allowed regions [a,b,l,p]	74	16.8%
Residues in generously allowed regions [~a,~b,~l,~p]	6	1.4%
Residues in disallowed regions	8	1.8%
	----	------
Number of non-glycine and non-proline residues	440	100.0%
Number of end-residues (excl. Gly and Pro)	2	
Number of glycine residues (shown as triangles)	33	
Number of proline residues	21	

Total number of residues	496	

Based on an analysis of 118 structures of resolution of at least 2.0 Angstroms and R-factor no greater than 20%, a good quality model would be expected to have over 90% in the most favoured regions.

SbHAK23_noHs_01_ramachand.ps

PROCHECK-NMR

Ramachandran Plot

SbHAK24_noHs (1 models)

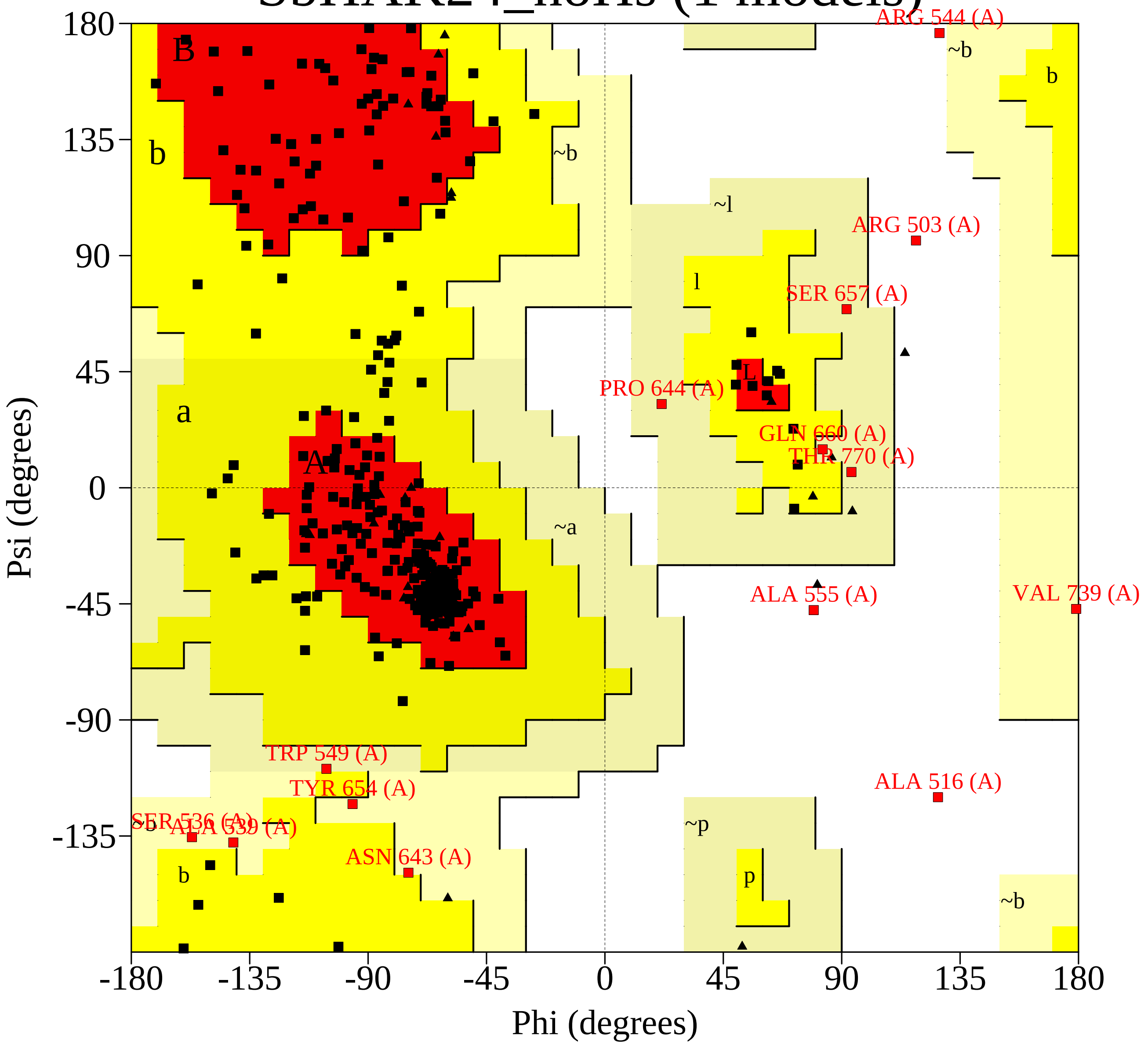

Plot statistics

Residues in most favoured regions [A,B,L]	291	81.3%
Residues in additional allowed regions [a,b,l,p]	54	15.1%
Residues in generously allowed regions [~a,~b,~l,~p]	9	2.5%
Residues in disallowed regions	4	1.1%
	----	------
Number of non-glycine and non-proline residues	358	100.0%
Number of end-residues (excl. Gly and Pro)	2	
Number of glycine residues (shown as triangles)	35	
Number of proline residues	13	

Total number of residues	408	

Based on an analysis of 118 structures of resolution of at least 2.0 Angstroms and R-factor no greater than 20%, a good quality model would be expected to have over 90% in the most favoured regions.

PROCHECK-NMR

Ramachandran Plot

SbHAK25_noHs (1 models)

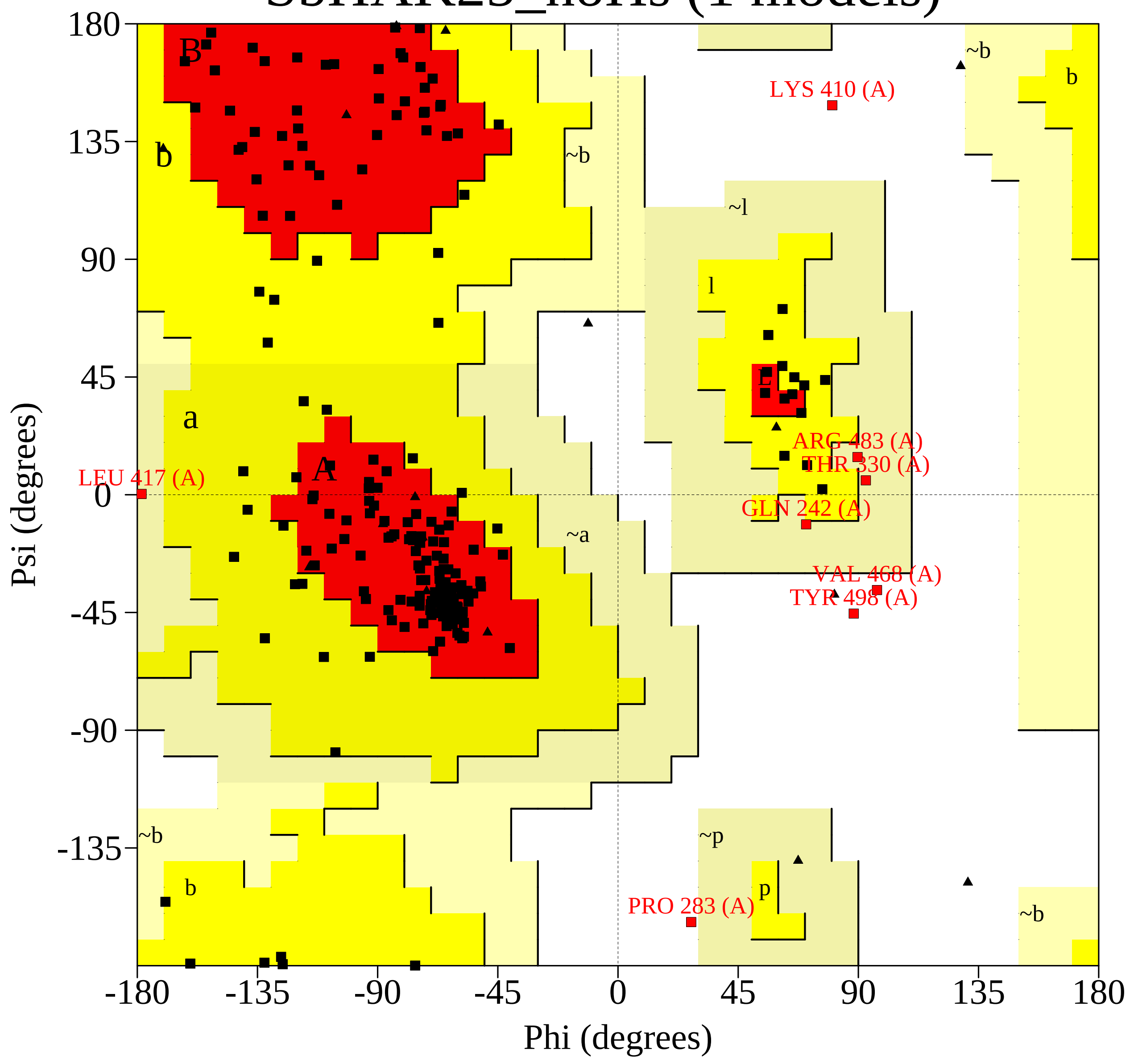

Plot statistics

Residues in most favoured regions [A,B,L]	211	83.1%
Residues in additional allowed regions [a,b,l,p]	36	14.2%
Residues in generously allowed regions [~a,~b,~l,~p]	4	1.6%
Residues in disallowed regions	3	1.2%
	----	------
Number of non-glycine and non-proline residues	254	100.0%
Number of end-residues (excl. Gly and Pro)	2	
Number of glycine residues (shown as triangles)	18	
Number of proline residues	13	

Total number of residues	287	

Based on an analysis of 118 structures of resolution of at least 2.0 Angstroms and R-factor no greater than 20%, a good quality model would be expected to have over 90% in the most favoured regions.

SbHAK25_noHs_01_ramachand.ps

PROCHECK-NMR

Ramachandran Plot

SbHAK26_noHs (1 models)

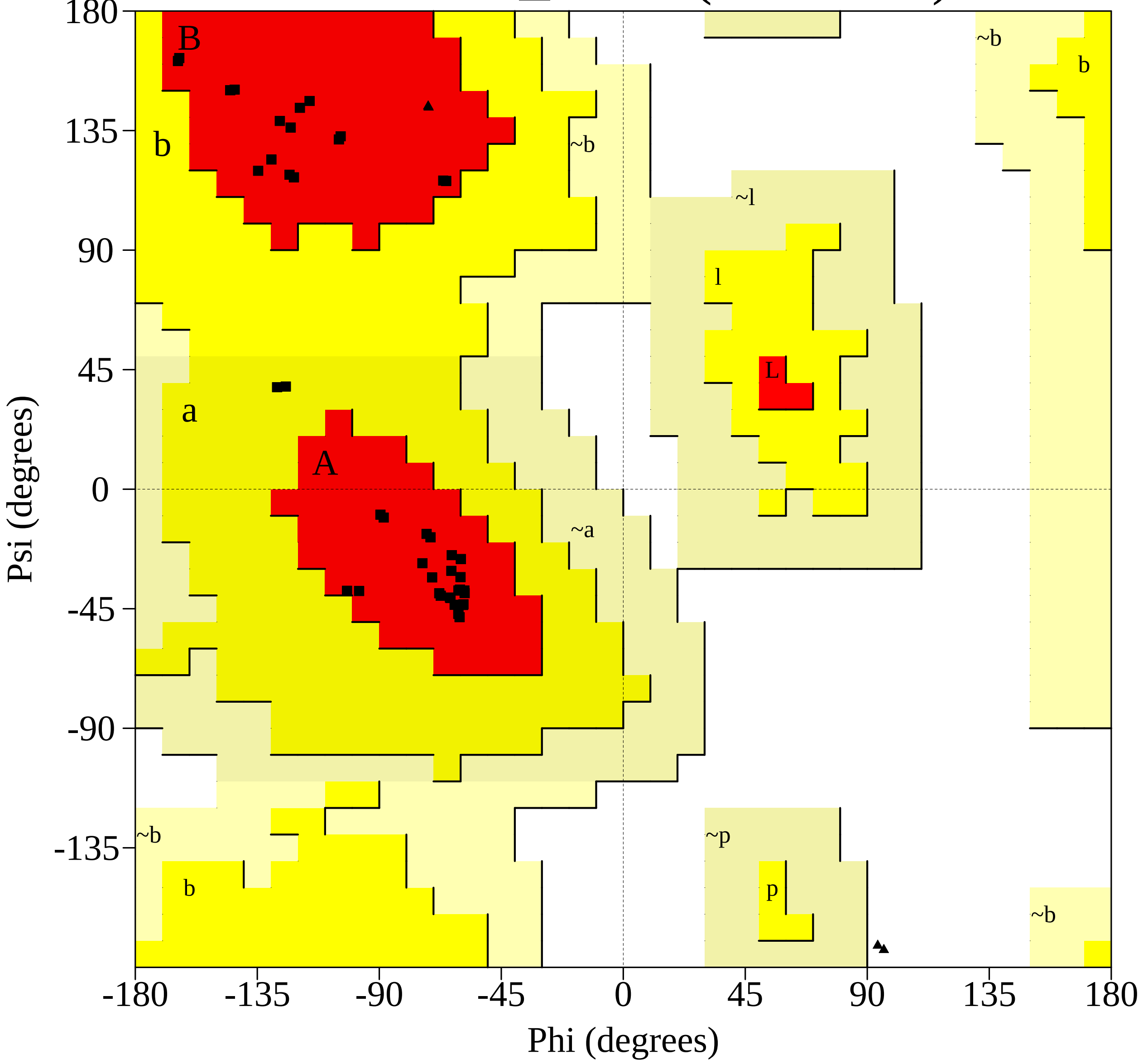

Plot statistics

Residues in most favoured regions [A,B,L]	40	95.2%
Residues in additional allowed regions [a,b,l,p]	2	4.8%
Residues in generously allowed regions [~a,~b,~l,~p]	0	0.0%
Residues in disallowed regions	0	0.0%
	----	------
Number of non-glycine and non-proline residues	42	100.0%
Number of end-residues (excl. Gly and Pro)	4	
Number of glycine residues (shown as triangles)	4	
Number of proline residues	2	

Total number of residues	52	

Based on an analysis of 118 structures of resolution of at least 2.0 Angstroms and R-factor no greater than 20%, a good quality model would be expected to have over 90% in the most favoured regions.

PROCHECK-NMR

Ramachandran Plot

SbHAK27_noHs (1 models)

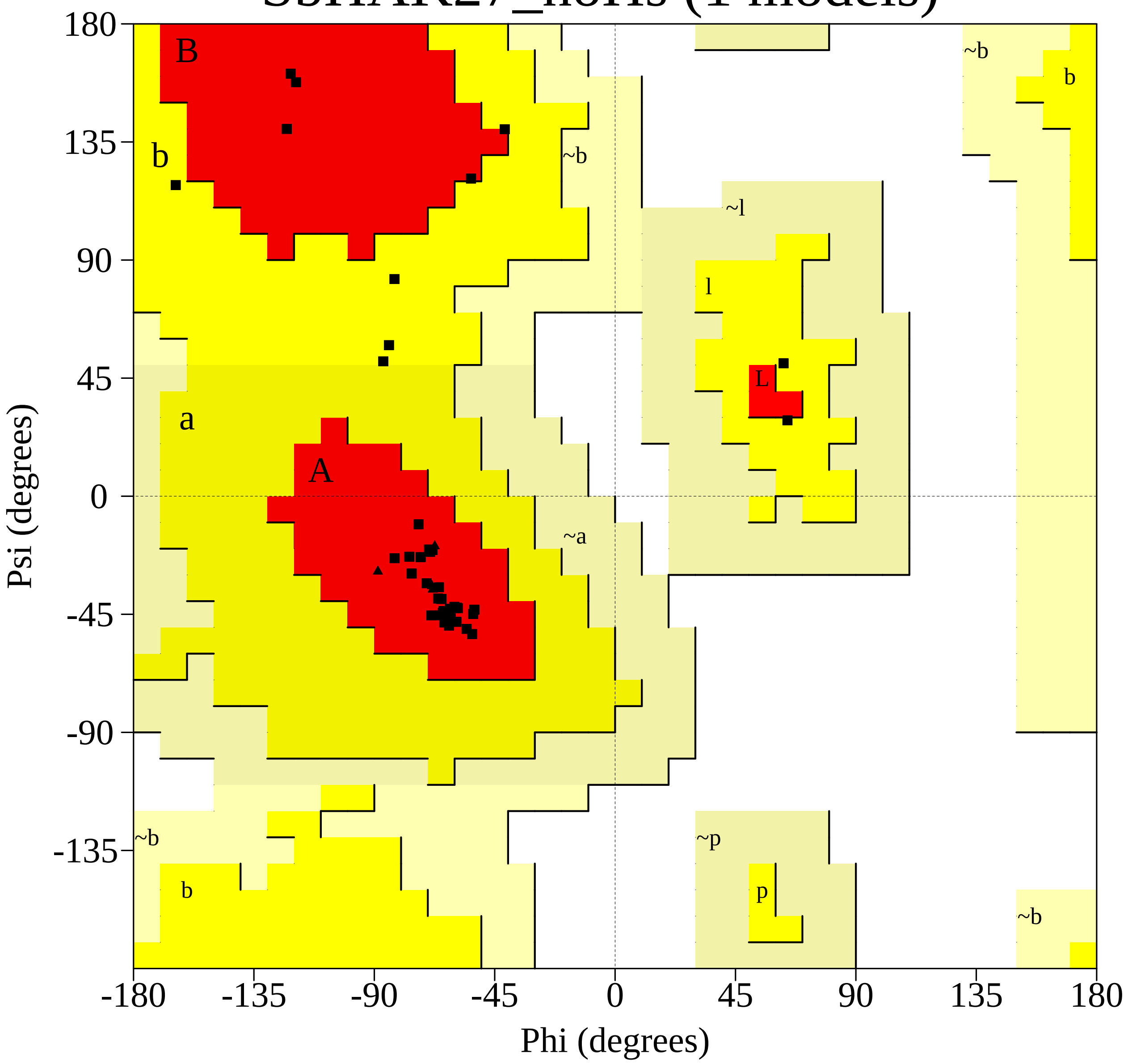

Plot statistics

Residues in most favoured regions [A,B,L]	34	85.0%
Residues in additional allowed regions [a,b,l,p]	6	15.0%
Residues in generously allowed regions [~a,~b,~l,~p]	0	0.0%
Residues in disallowed regions	0	0.0%
	----	------
Number of non-glycine and non-proline residues	40	100.0%
Number of end-residues (excl. Gly and Pro)	2	
Number of glycine residues (shown as triangles)	4	
Number of proline residues	3	

Total number of residues	49	

Based on an analysis of 118 structures of resolution of at least 2.0 Angstroms and R-factor no greater than 20%, a good quality model would be expected to have over 90% in the most favoured regions.

SbHAK27_noHs_01_ramachand.ps

PROCHECK-NMR

Ramachandran Plot

SbHKT2_noHs (1 models)

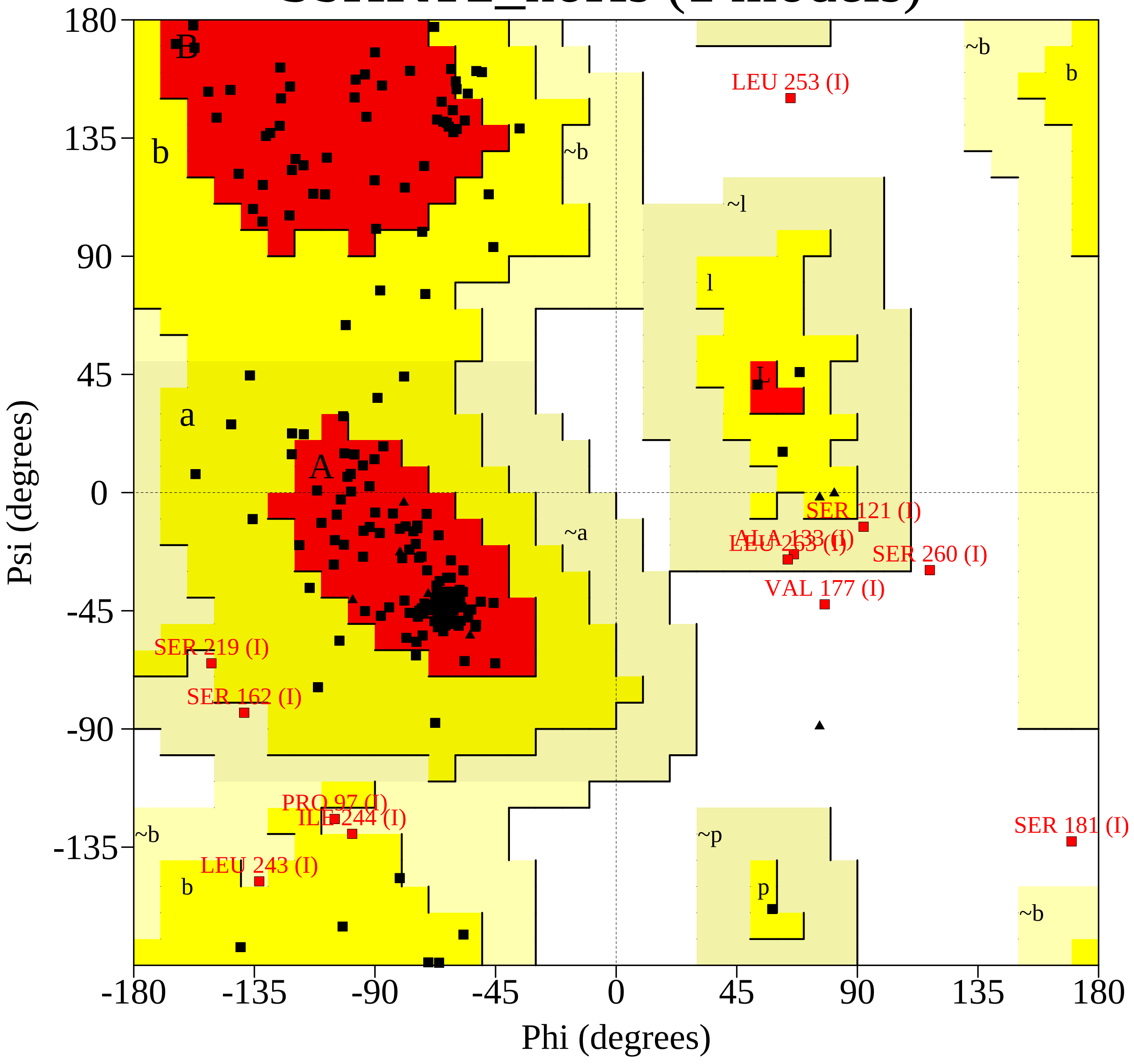

Plot statistics

Residues in most favoured regions [A,B,L]	171	78.8%
Residues in additional allowed regions [a,b,l,p]	35	16.1%
Residues in generously allowed regions [~a,~b,~l,~p]	7	3.2%
Residues in disallowed regions	4	1.8%
	----	------
Number of non-glycine and non-proline residues	217	100.0%
Number of end-residues (excl. Gly and Pro)	2	
Number of glycine residues (shown as triangles)	10	
Number of proline residues	5	

Total number of residues	234	

Based on an analysis of 118 structures of resolution of at least 2.0 Angstroms and R-factor no greater than 20%, a good quality model would be expected to have over 90% in the most favoured regions.

SbHKT2_noHs_01_ramachand.ps

PROCHECK-NMR

Ramachandran Plot

SbHKT3_noHs (1 models)

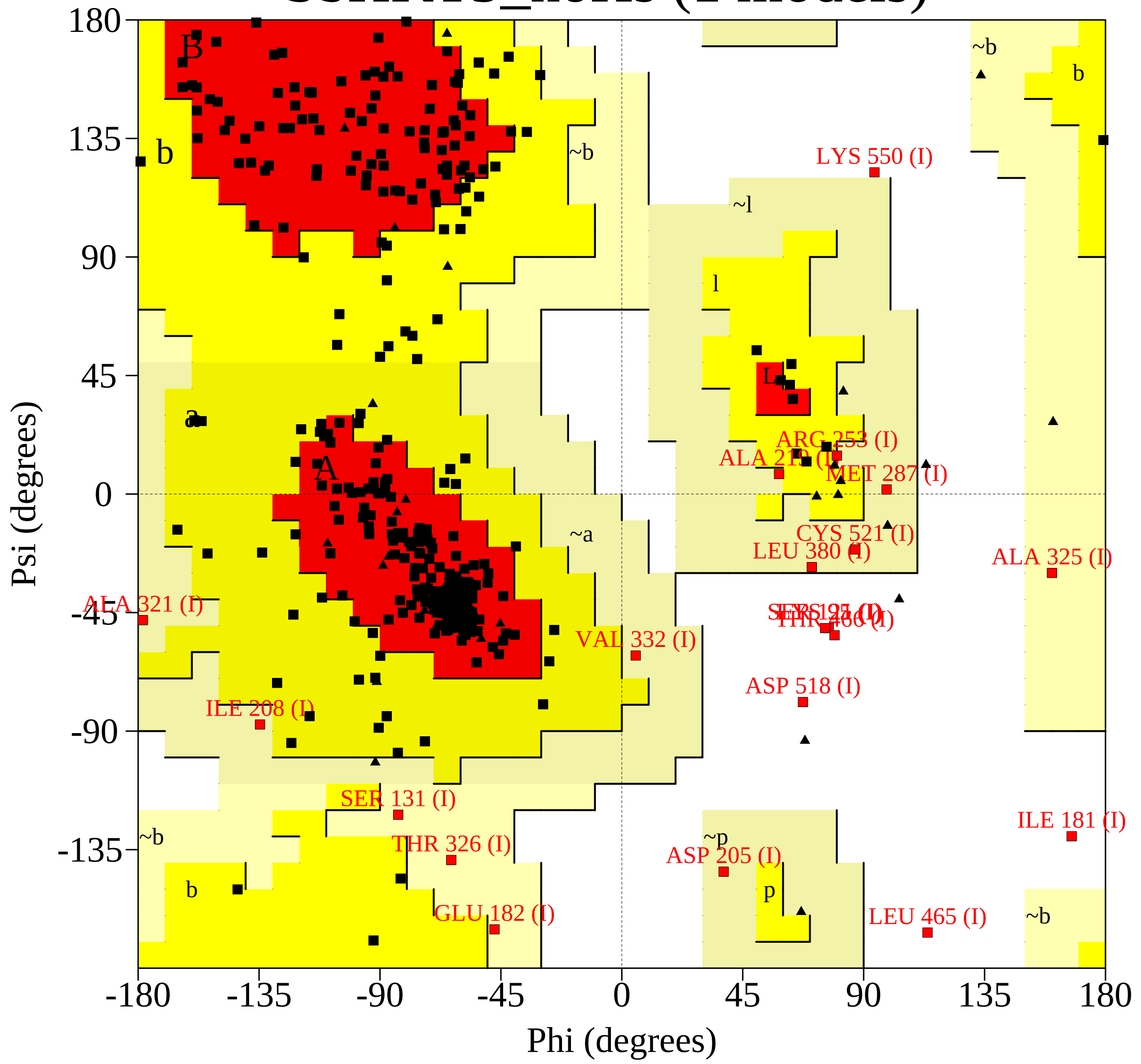

Plot statistics

Residues in most favoured regions [A,B,L]	346	79.9%
Residues in additional allowed regions [a,b,l,p]	67	15.5%
Residues in generously allowed regions [~a,~b,~l,~p]	13	3.0%
Residues in disallowed regions	7	1.6%
	----	------
Number of non-glycine and non-proline residues	433	100.0%
Number of end-residues (excl. Gly and Pro)	2	
Number of glycine residues (shown as triangles)	35	
Number of proline residues	15	

Total number of residues	485	

Based on an analysis of 118 structures of resolution of at least 2.0 Angstroms and R-factor no greater than 20%, a good quality model would be expected to have over 90% in the most favoured regions.

SbHKT3_noHs_01_ramachand.ps

PROCHECK-NMR

Ramachandran Plot

SbHKT4_noHs (1 models)

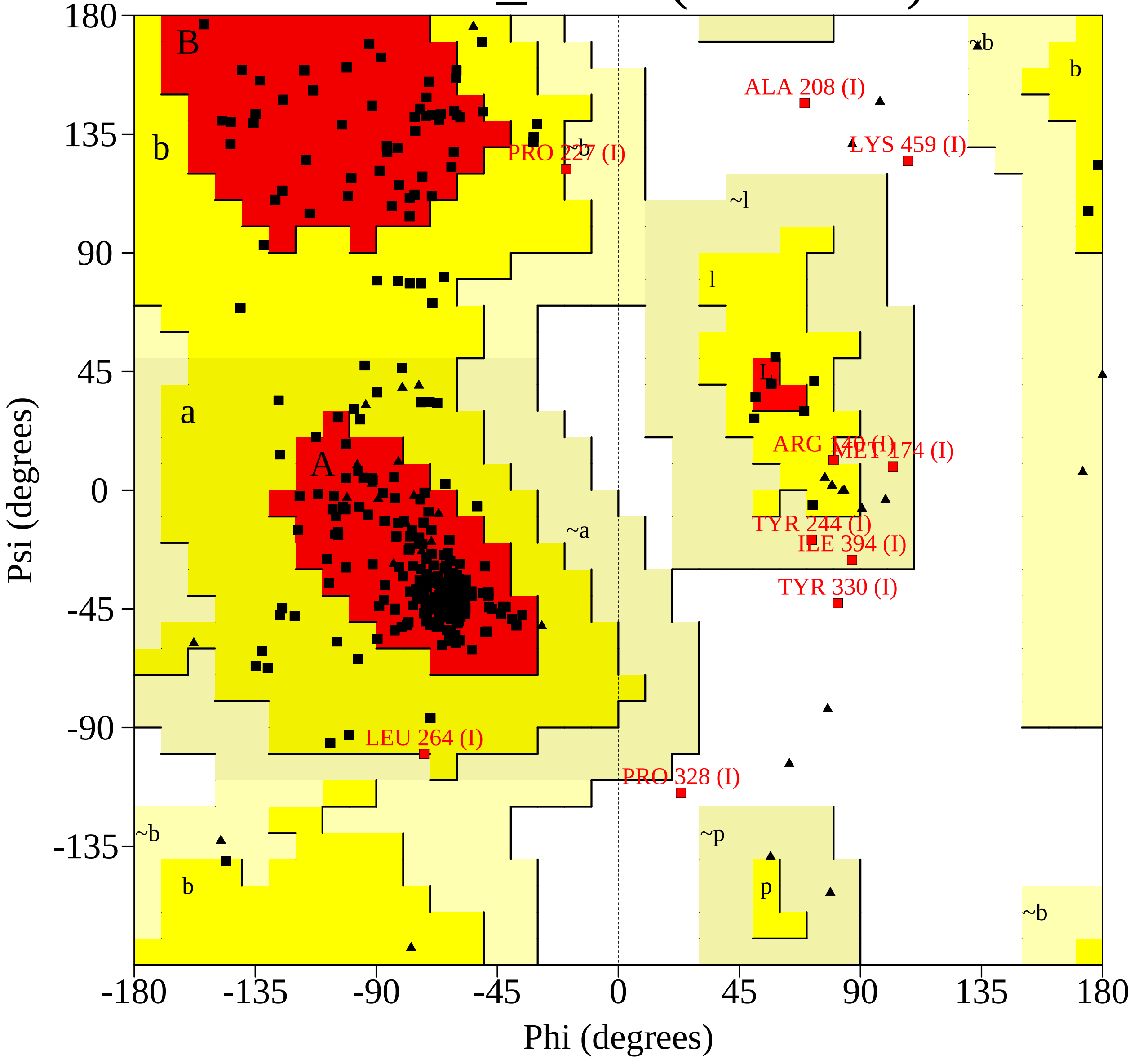

Plot statistics

Residues in most favoured regions [A,B,L]	262	84.5%
Residues in additional allowed regions [a,b,l,p]	40	12.9%
Residues in generously allowed regions [~a,~b,~l,~p]	5	1.6%
Residues in disallowed regions	3	1.0%
	----	------
Number of non-glycine and non-proline residues	310	100.0%
Number of end-residues (excl. Gly and Pro)	2	
Number of glycine residues (shown as triangles)	37	
Number of proline residues	14	

Total number of residues	363	

Based on an analysis of 118 structures of resolution of at least 2.0 Angstroms and R-factor no greater than 20%, a good quality model would be expected to have over 90% in the most favoured regions.

PROCHECK-NMR

Ramachandran Plot

SbHKT5_noHs (1 models)

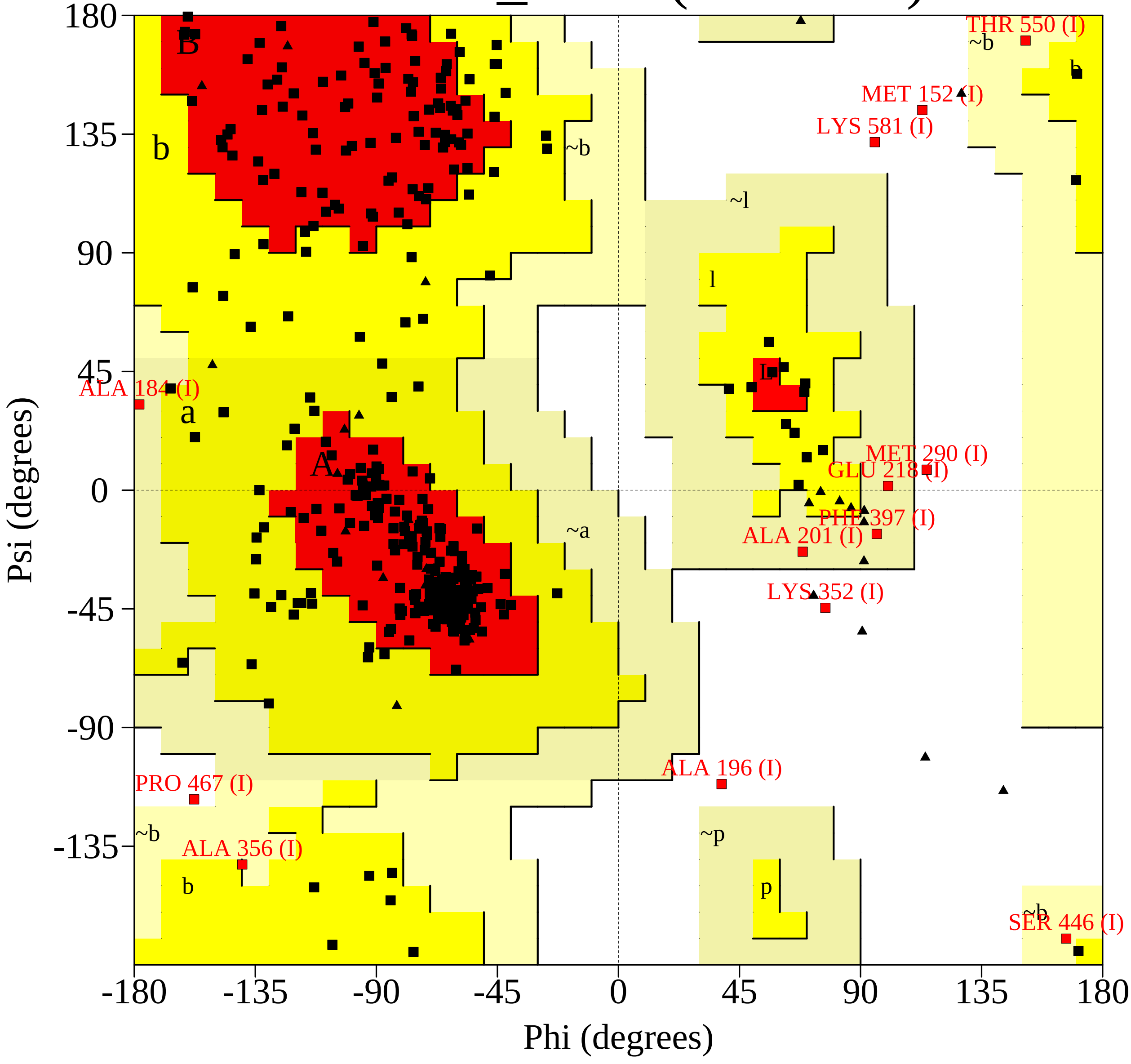

Plot statistics

Residues in most favoured regions [A,B,L]	369	82.4%
Residues in additional allowed regions [a,b,l,p]	67	15.0%
Residues in generously allowed regions [~a,~b,~l,~p]	7	1.6%
Residues in disallowed regions	5	1.1%
	----	------
Number of non-glycine and non-proline residues	448	100.0%
Number of end-residues (excl. Gly and Pro)	2	
Number of glycine residues (shown as triangles)	38	
Number of proline residues	23	

Total number of residues	511	

Based on an analysis of 118 structures of resolution of at least 2.0 Angstroms and R-factor no greater than 20%, a good quality model would be expected to have over 90% in the most favoured regions.

SbHKT5_noHs_01_ramachand.ps

PROCHECK-NMR

Ramachandran Plot

SbAKT1_noHs (1 models)

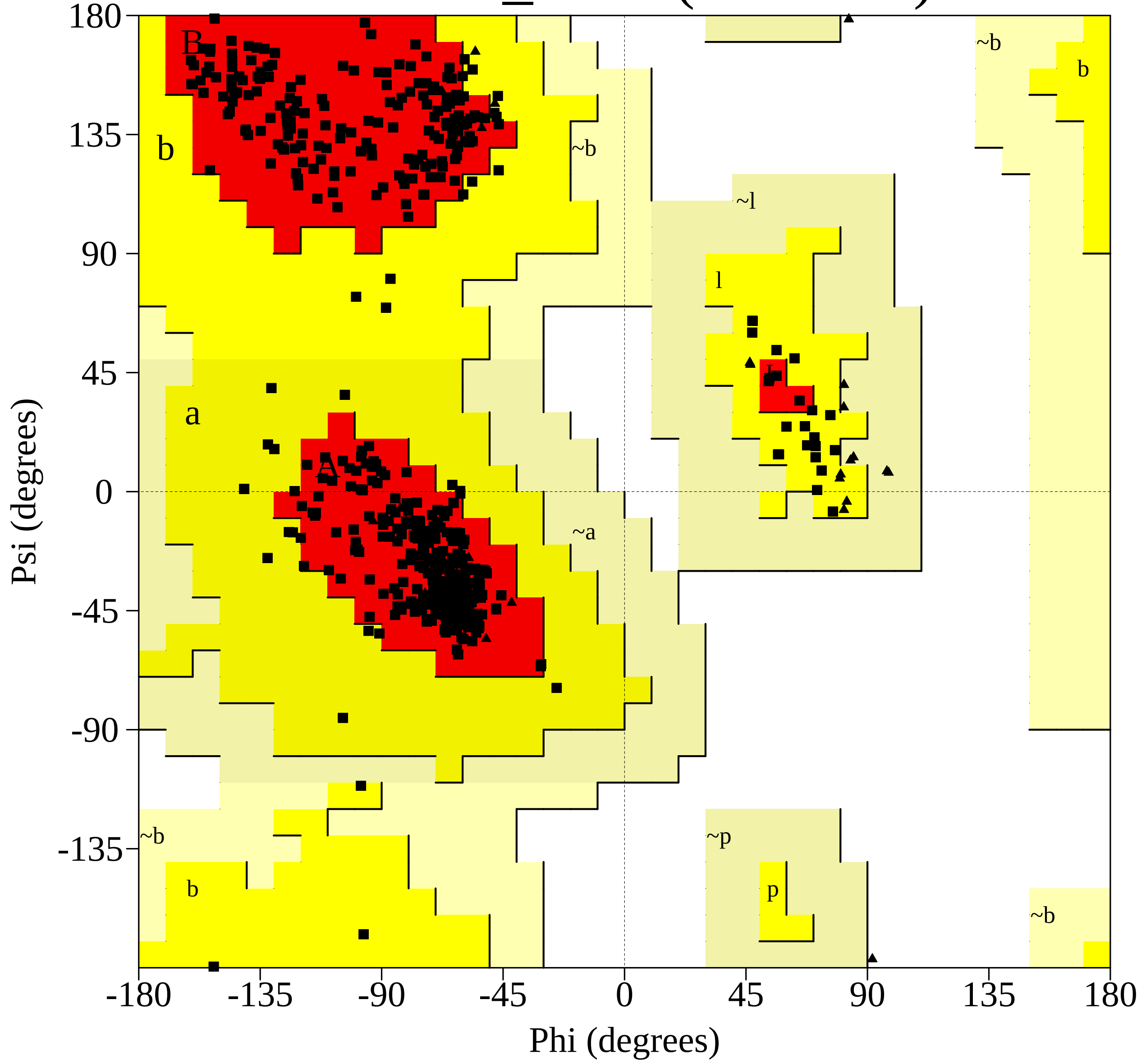

Plot statistics

Residues in most favoured regions [A,B,L]	1529	95.3%
Residues in additional allowed regions [a,b,l,p]	75	4.7%
Residues in generously allowed regions [~a,~b,~l,~p]	0	0.0%
Residues in disallowed regions	0	0.0%
	----	------
Number of non-glycine and non-proline residues	1604	100.0%
Number of end-residues (excl. Gly and Pro)	6	
Number of glycine residues (shown as triangles)	72	
Number of proline residues	66	

Total number of residues	1748	

Based on an analysis of 118 structures of resolution of at least 2.0 Angstroms and R-factor no greater than 20%, a good quality model would be expected to have over 90% in the most favoured regions.

PROCHECK-NMR

Ramachandran Plot

SbAKT2_noHs (1 models)

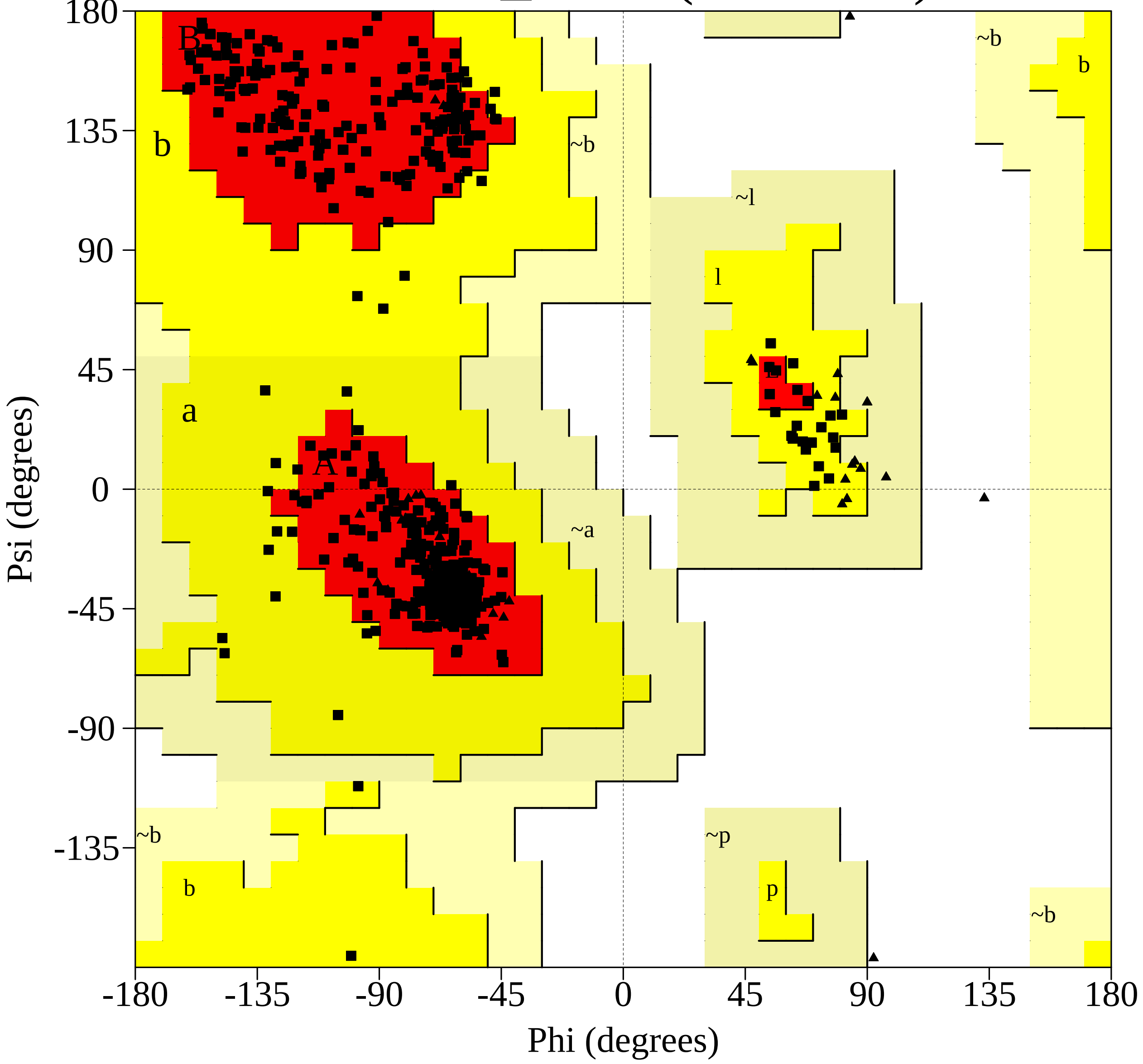

Plot statistics

Residues in most favoured regions [A,B,L]	1354	94.7%
Residues in additional allowed regions [a,b,l,p]	76	5.3%
Residues in generously allowed regions [~a,~b,~l,~p]	0	0.0%
Residues in disallowed regions	0	0.0%
	----	------
Number of non-glycine and non-proline residues	1430	100.0%
Number of end-residues (excl. Gly and Pro)	8	
Number of glycine residues (shown as triangles)	88	
Number of proline residues	60	

Total number of residues	1586	

Based on an analysis of 118 structures of resolution of at least 2.0 Angstroms and R-factor no greater than 20%, a good quality model would be expected to have over 90% in the most favoured regions.

SbAKT2_noHs_01_ramachand.ps

PROCHECK-NMR

Ramachandran Plot

SbAKT3_noHs (1 models)

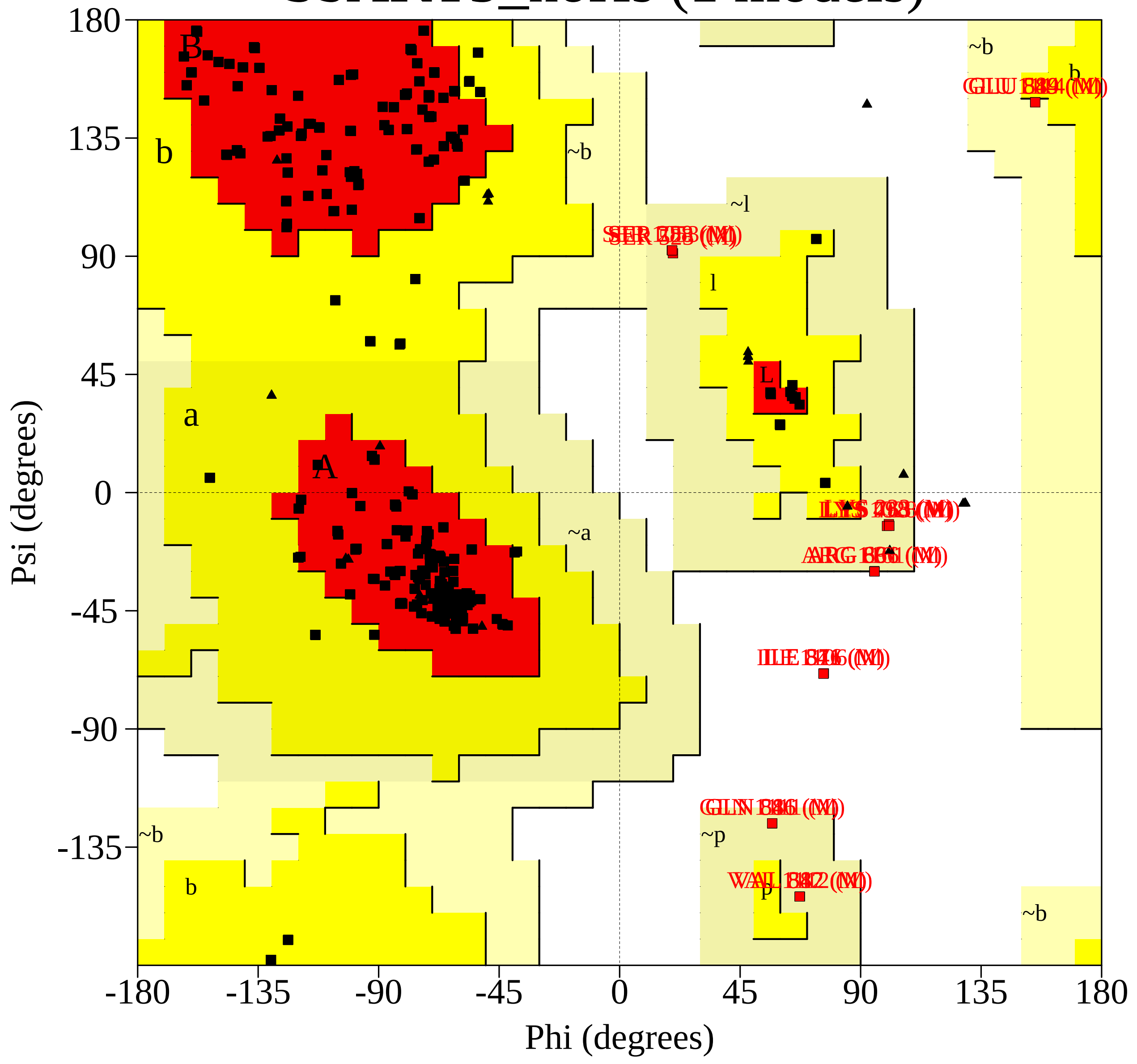

Plot statistics

Residues in most favoured regions [A,B,L]	819	89.4%
Residues in additional allowed regions [a,b,l,p]	69	7.5%
Residues in generously allowed regions [~a,~b,~l,~p]	21	2.3%
Residues in disallowed regions	7	0.8%
	----	------
Number of non-glycine and non-proline residues	916	100.0%
Number of end-residues (excl. Gly and Pro)	8	
Number of glycine residues (shown as triangles)	60	
Number of proline residues	36	

Total number of residues	1020	

Based on an analysis of 118 structures of resolution of at least 2.0 Angstroms and R-factor no greater than 20%, a good quality model would be expected to have over 90% in the most favoured regions.

SbAKT3_noHs_01_ramachand.ps

PROCHECK-NMR

Ramachandran Plot

SbAKT4_noHs (1 models)

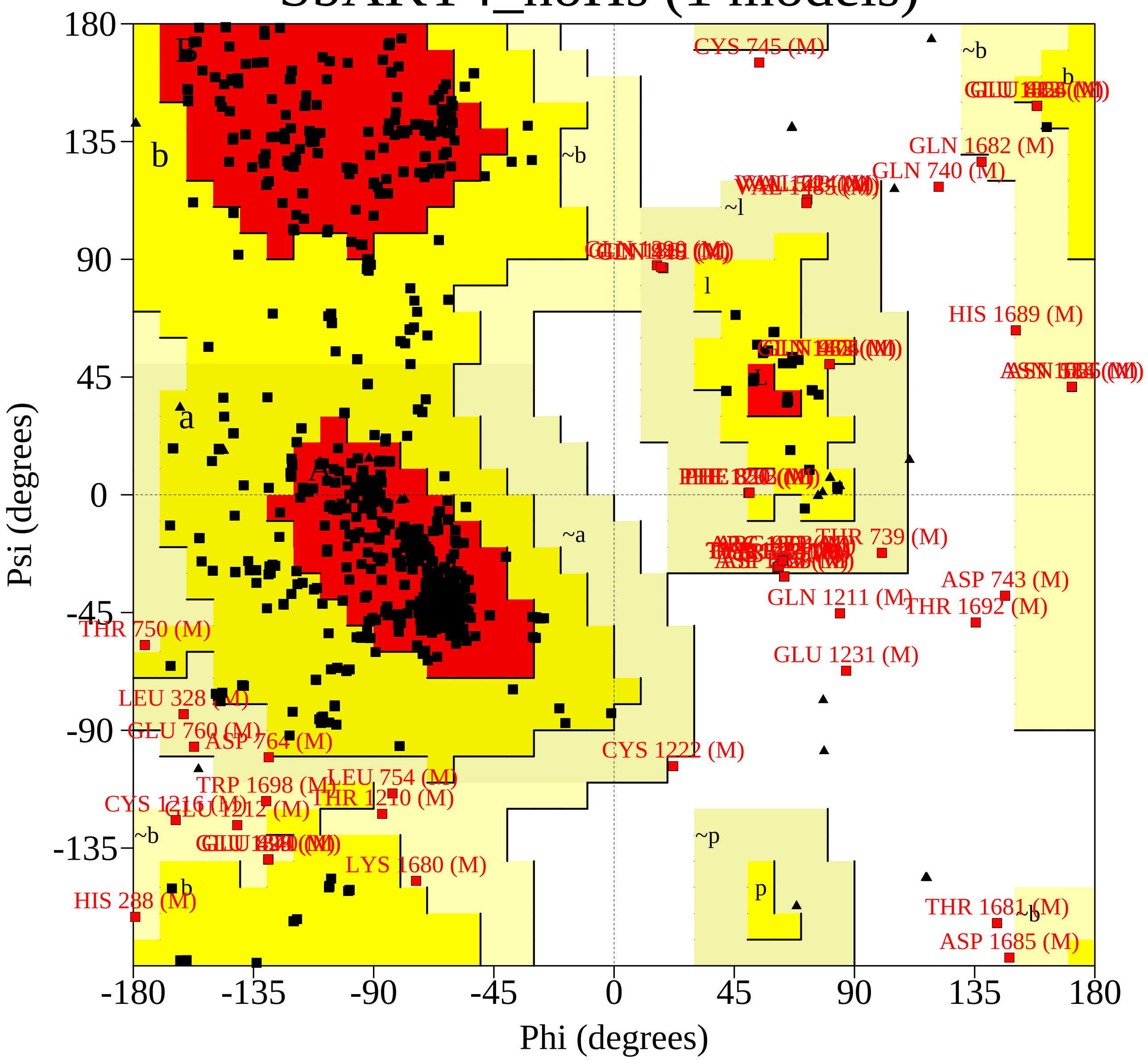

Plot statistics

Residues in most favoured regions [A,B,L]	1350	80.2%
Residues in additional allowed regions [a,b,l,p]	271	16.1%
Residues in generously allowed regions [~a,~b,~l,~p]	49	2.9%
Residues in disallowed regions	14	0.8%
	----	------
Number of non-glycine and non-proline residues	1684	100.0%
Number of end-residues (excl. Gly and Pro)	8	
Number of glycine residues (shown as triangles)	92	
Number of proline residues	60	

Total number of residues	1844	

Based on an analysis of 118 structures of resolution of at least 2.0 Angstroms and R-factor no greater than 20%, a good quality model would be expected to have over 90% in the most favoured regions.

SbAKT4_noHs_01_ramachand.ps

PROCHECK-NMR

Ramachandran Plot

SbAKT5_noHs (1 models)

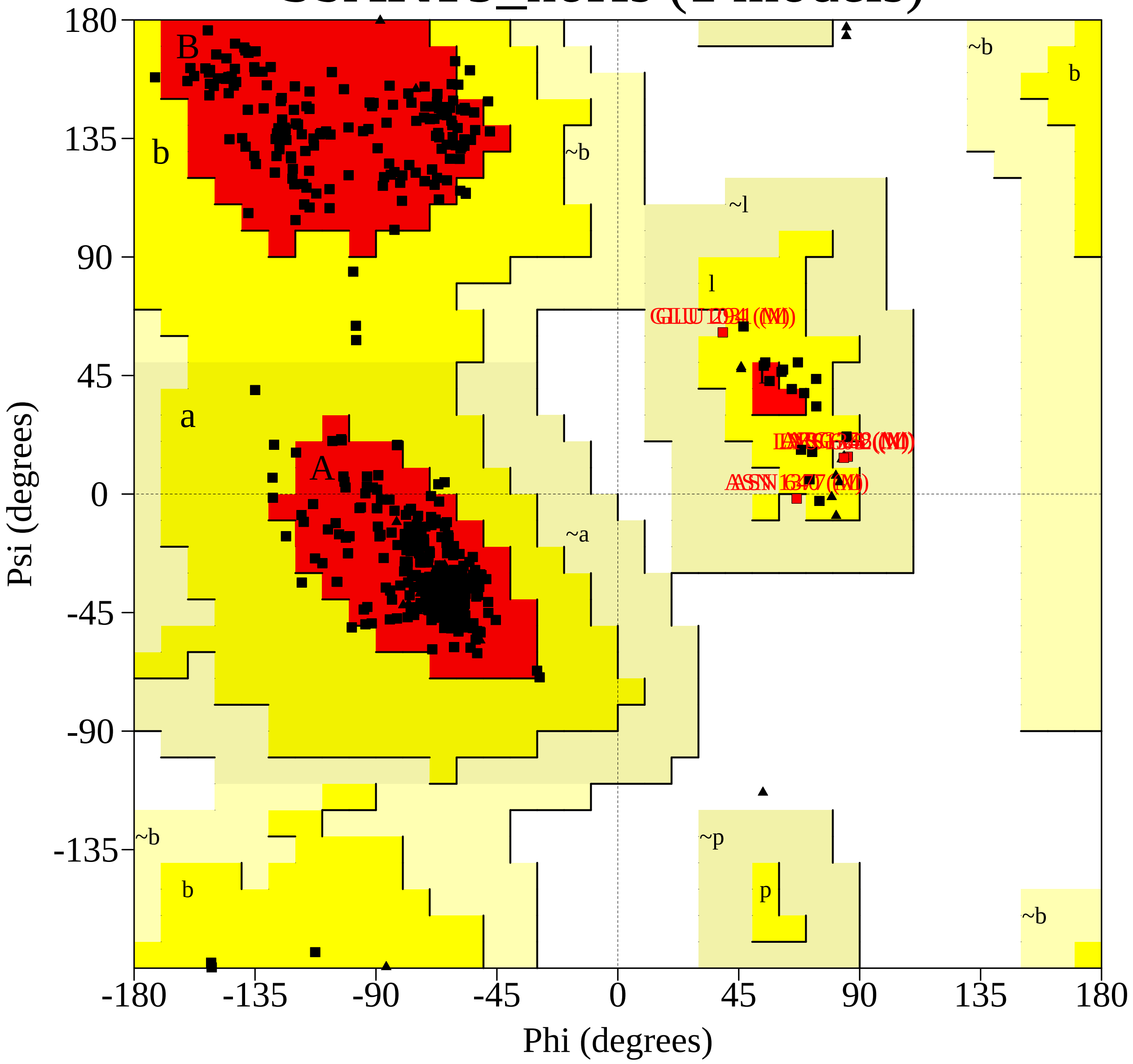

Plot statistics

Residues in most favoured regions [A,B,L]	1250	95.1%
Residues in additional allowed regions [a,b,l,p]	56	4.3%
Residues in generously allowed regions [~a,~b,~l,~p]	8	0.6%
Residues in disallowed regions	0	0.0%
	----	------
Number of non-glycine and non-proline residues	1314	100.0%
Number of end-residues (excl. Gly and Pro)	8	
Number of glycine residues (shown as triangles)	64	
Number of proline residues	48	

Total number of residues	1434	

Based on an analysis of 118 structures of resolution of at least 2.0 Angstroms and R-factor no greater than 20%, a good quality model would be expected to have over 90% in the most favoured regions.

PROCHECK-NMR

Ramachandran Plot

SbAKT6_noHs (1 models)

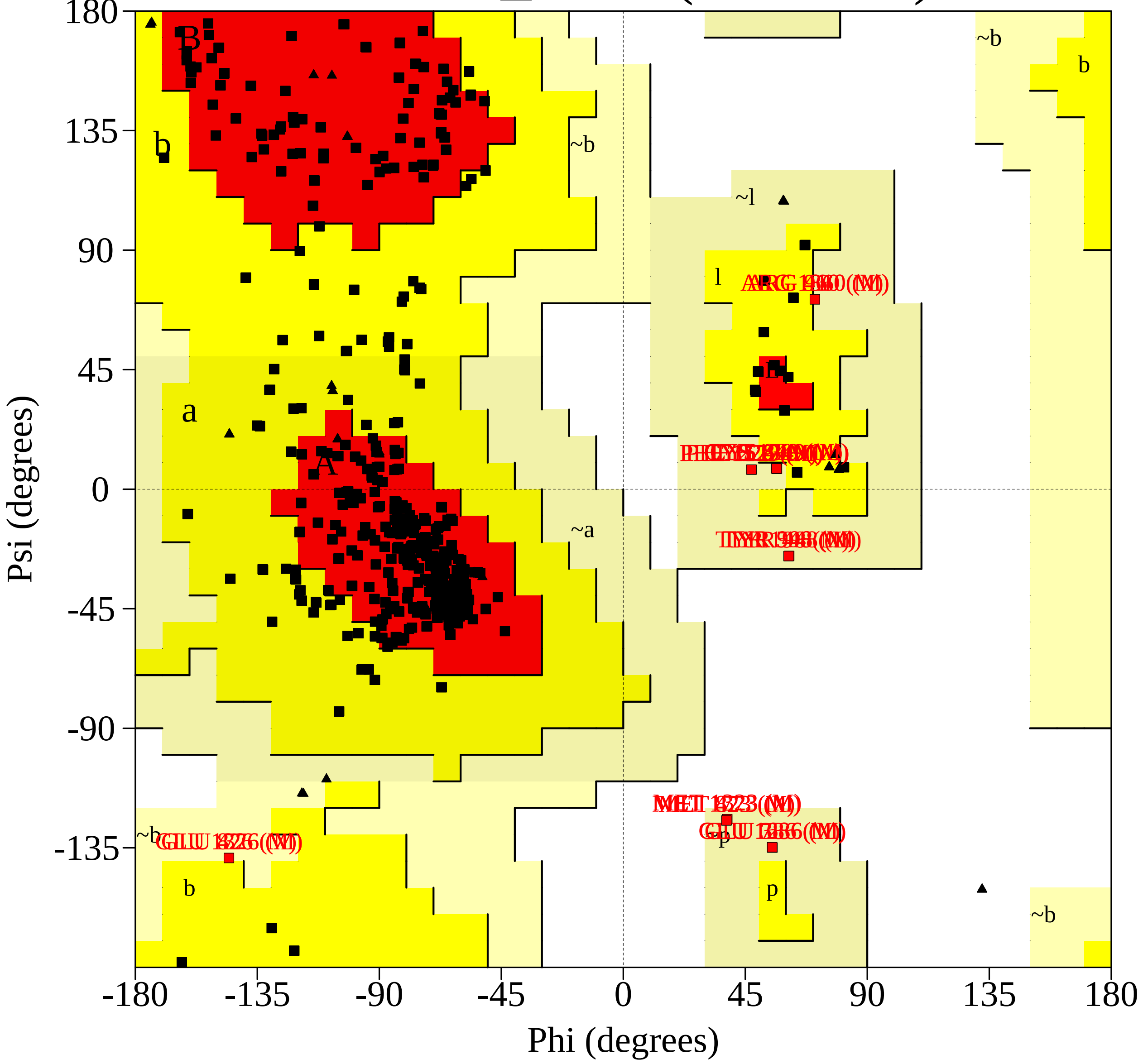

Plot statistics

Residues in most favoured regions [A,B,L]	1348	83.2%
Residues in additional allowed regions [a,b,l,p]	244	15.1%
Residues in generously allowed regions [~a,~b,~l,~p]	28	1.7%
Residues in disallowed regions	0	0.0%
	----	------
Number of non-glycine and non-proline residues	1620	100.0%
Number of end-residues (excl. Gly and Pro)	8	
Number of glycine residues (shown as triangles)	80	
Number of proline residues	52	

Total number of residues	1760	

Based on an analysis of 118 structures of resolution of at least 2.0 Angstroms and R-factor no greater than 20%, a good quality model would be expected to have over 90% in the most favoured regions.

PROCHECK-NMR

Ramachandran Plot

SbAKT7_noHs (1 models)

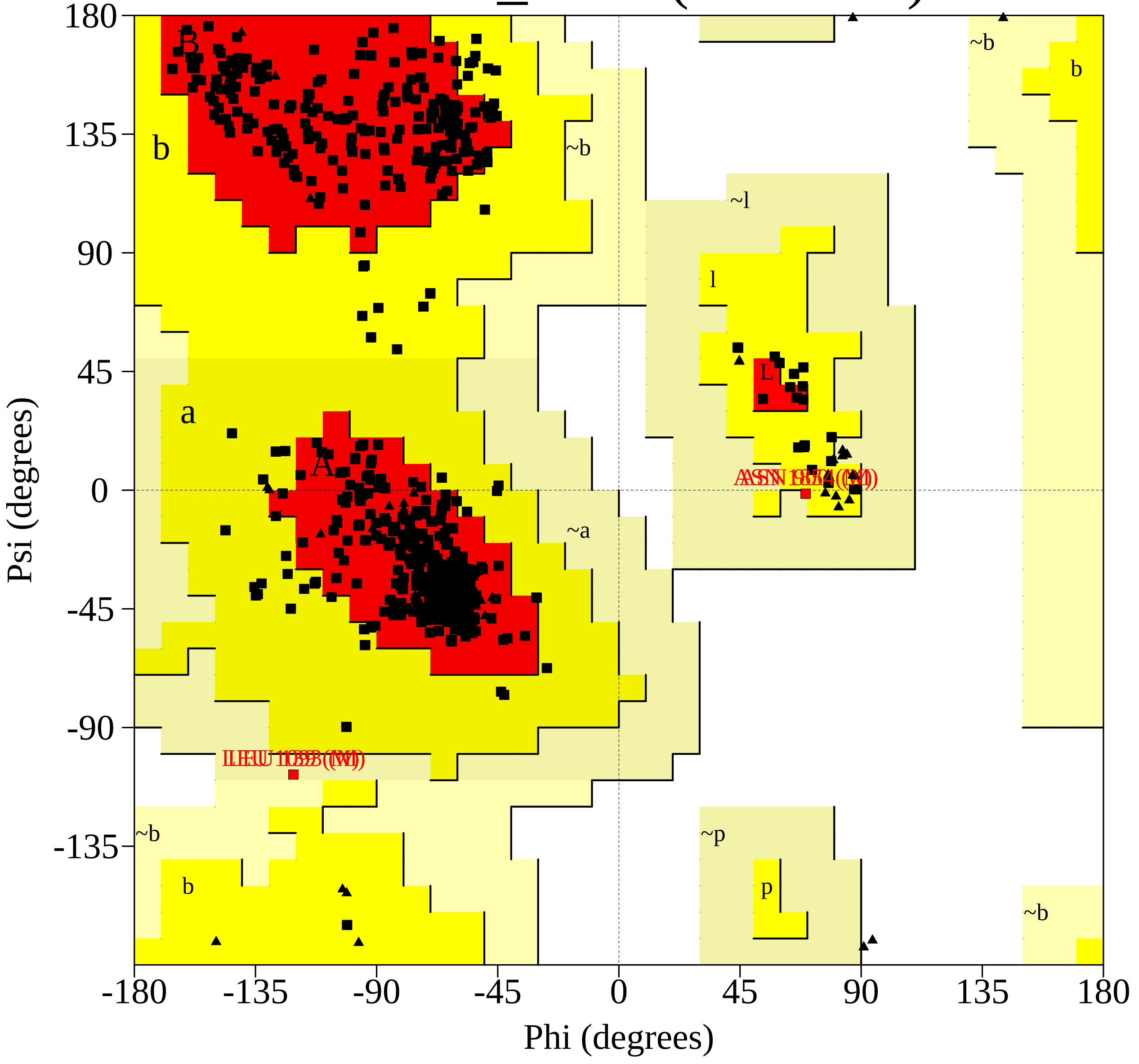

Plot statistics

Residues in most favoured regions [A,B,L]	1575	94.3%
Residues in additional allowed regions [a,b,l,p]	91	5.4%
Residues in generously allowed regions [~a,~b,~l,~p]	4	0.2%
Residues in disallowed regions	0	0.0%
	----	------
Number of non-glycine and non-proline residues	1670	100.0%
Number of end-residues (excl. Gly and Pro)	4	
Number of glycine residues (shown as triangles)	118	
Number of proline residues	76	

Total number of residues	1868	

Based on an analysis of 118 structures of resolution of at least 2.0 Angstroms and R-factor no greater than 20%, a good quality model would be expected to have over 90% in the most favoured regions.

PROCHECK-NMR

Ramachandran Plot

SbAKT8_noHs (1 models)

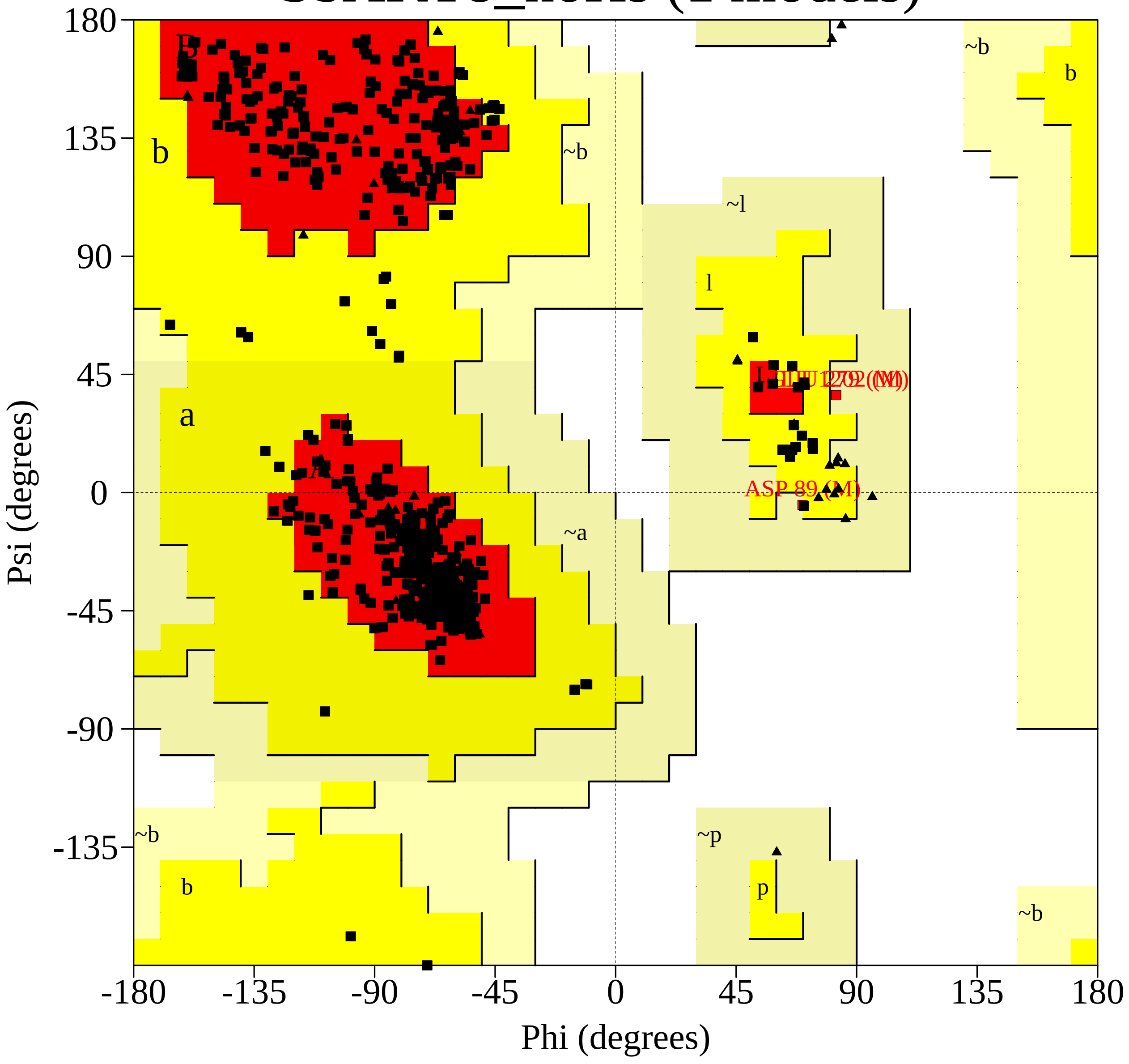

Plot statistics

Residues in most favoured regions [A,B,L]	1567	95.4%
Residues in additional allowed regions [a,b,l,p]	72	4.4%
Residues in generously allowed regions [~a,~b,~l,~p]	3	0.2%
Residues in disallowed regions	0	0.0%
	----	------
Number of non-glycine and non-proline residues	1642	100.0%
Number of end-residues (excl. Gly and Pro)	8	
Number of glycine residues (shown as triangles)	100	
Number of proline residues	56	

Total number of residues	1806	

Based on an analysis of 118 structures of resolution of at least 2.0 Angstroms and R-factor no greater than 20%, a good quality model would be expected to have over 90% in the most favoured regions.

PROCHECK-NMR

Ramachandran Plot

SbAKT9_noHs (1 models)

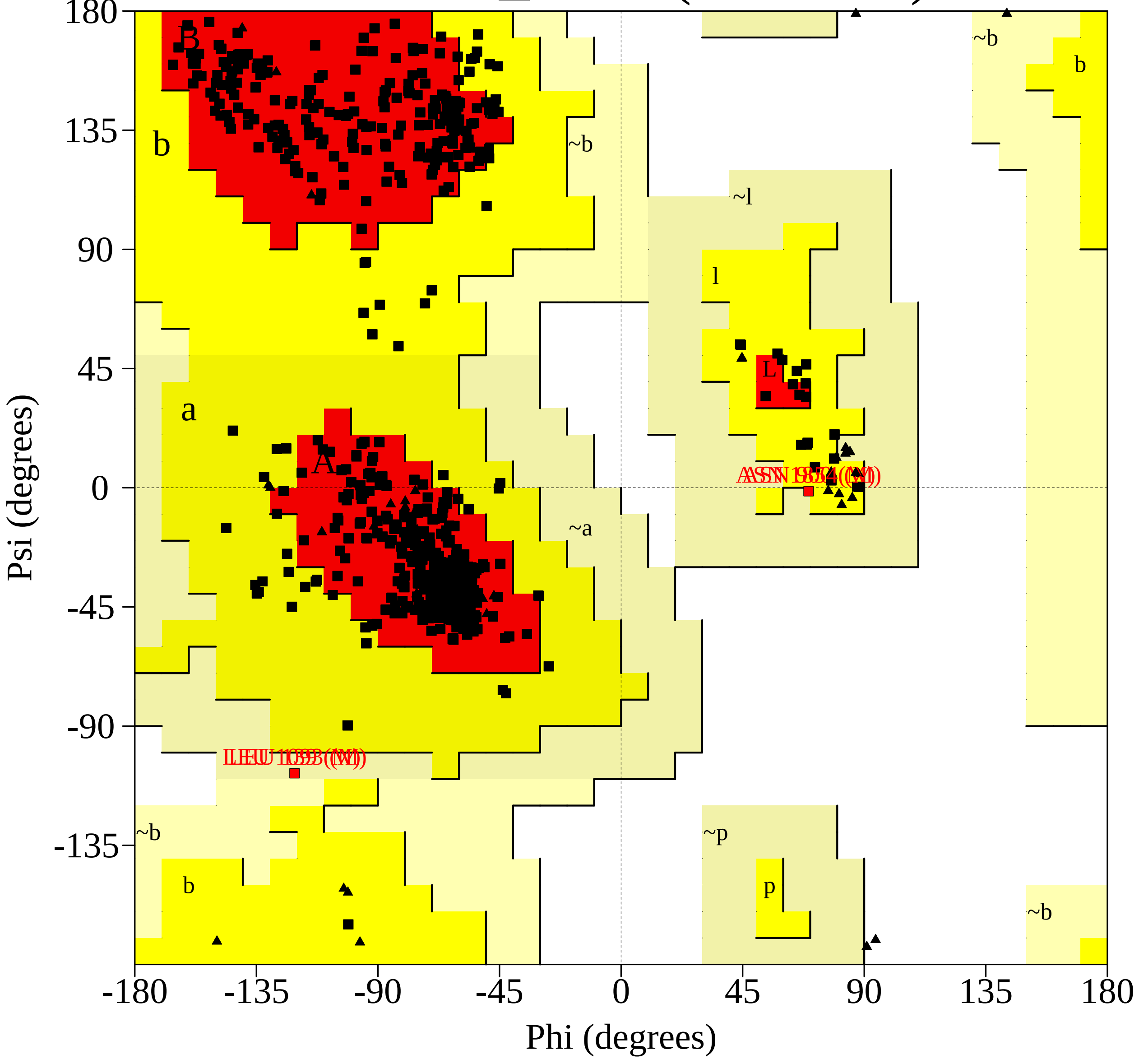

Plot statistics

Residues in most favoured regions [A,B,L]	1575	94.3%
Residues in additional allowed regions [a,b,l,p]	91	5.4%
Residues in generously allowed regions [~a,~b,~l,~p]	4	0.2%
Residues in disallowed regions	0	0.0%
	----	------
Number of non-glycine and non-proline residues	1670	100.0%
Number of end-residues (excl. Gly and Pro)	4	
Number of glycine residues (shown as triangles)	118	
Number of proline residues	76	

Total number of residues	1868	

Based on an analysis of 118 structures of resolution of at least 2.0 Angstroms and R-factor no greater than 20%, a good quality model would be expected to have over 90% in the most favoured regions.

SbAKT9_noHs_01_ramachand.ps

PROCHECK-NMR

Ramachandran Plot

SbKAT1_noHs (1 models)

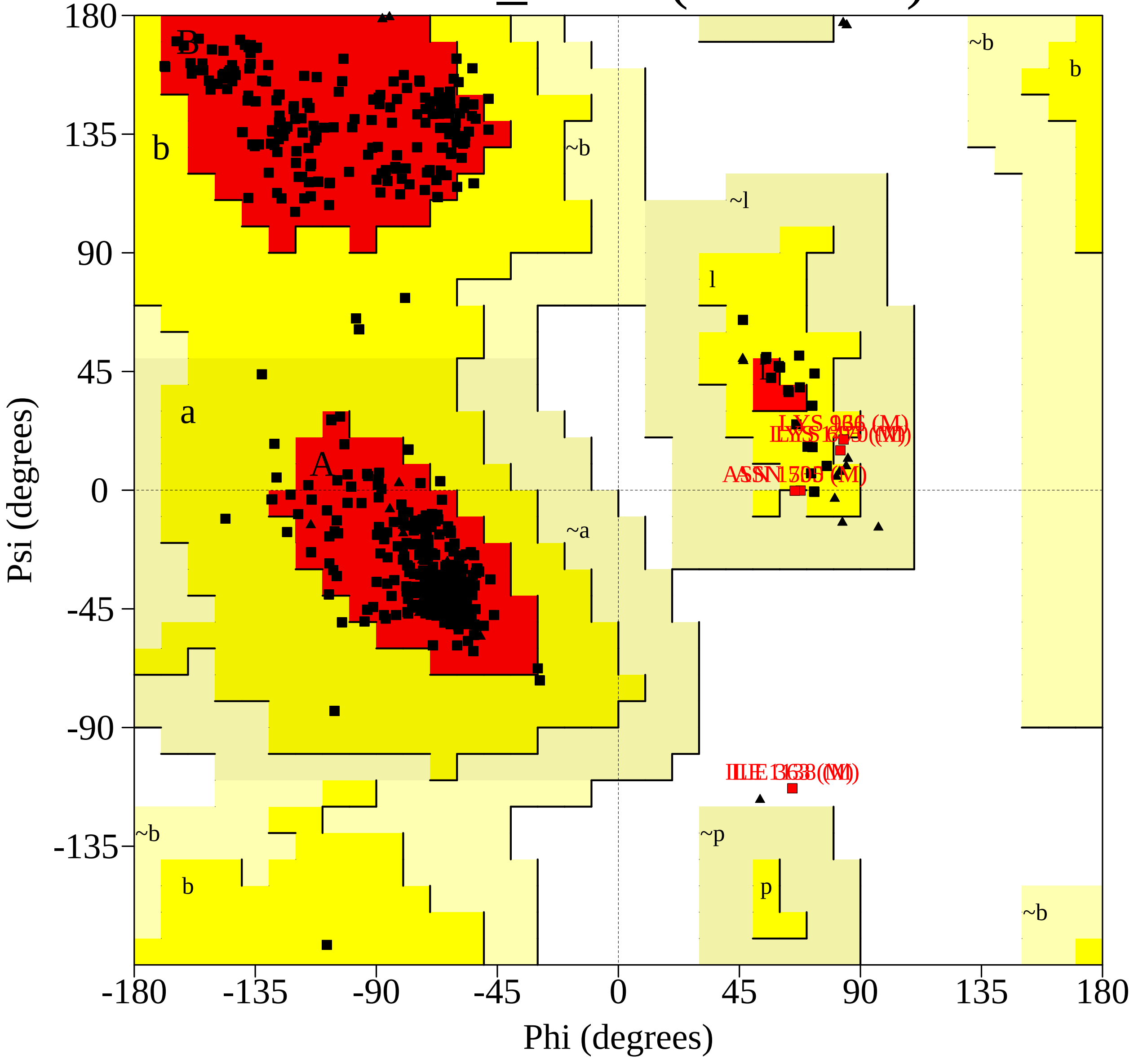

Plot statistics

Residues in most favoured regions [A,B,L]	1319	95.4%
Residues in additional allowed regions [a,b,l,p]	55	4.0%
Residues in generously allowed regions [~a,~b,~l,~p]	6	0.4%
Residues in disallowed regions	2	0.1%
	----	------
Number of non-glycine and non-proline residues	1382	100.0%
Number of end-residues (excl. Gly and Pro)	4	
Number of glycine residues (shown as triangles)	72	
Number of proline residues	52	

Total number of residues	1510	

Based on an analysis of 118 structures of resolution of at least 2.0 Angstroms and R-factor no greater than 20%, a good quality model would be expected to have over 90% in the most favoured regions.

PROCHECK-NMR

Ramachandran Plot

SbKAT2_noHs (1 models)

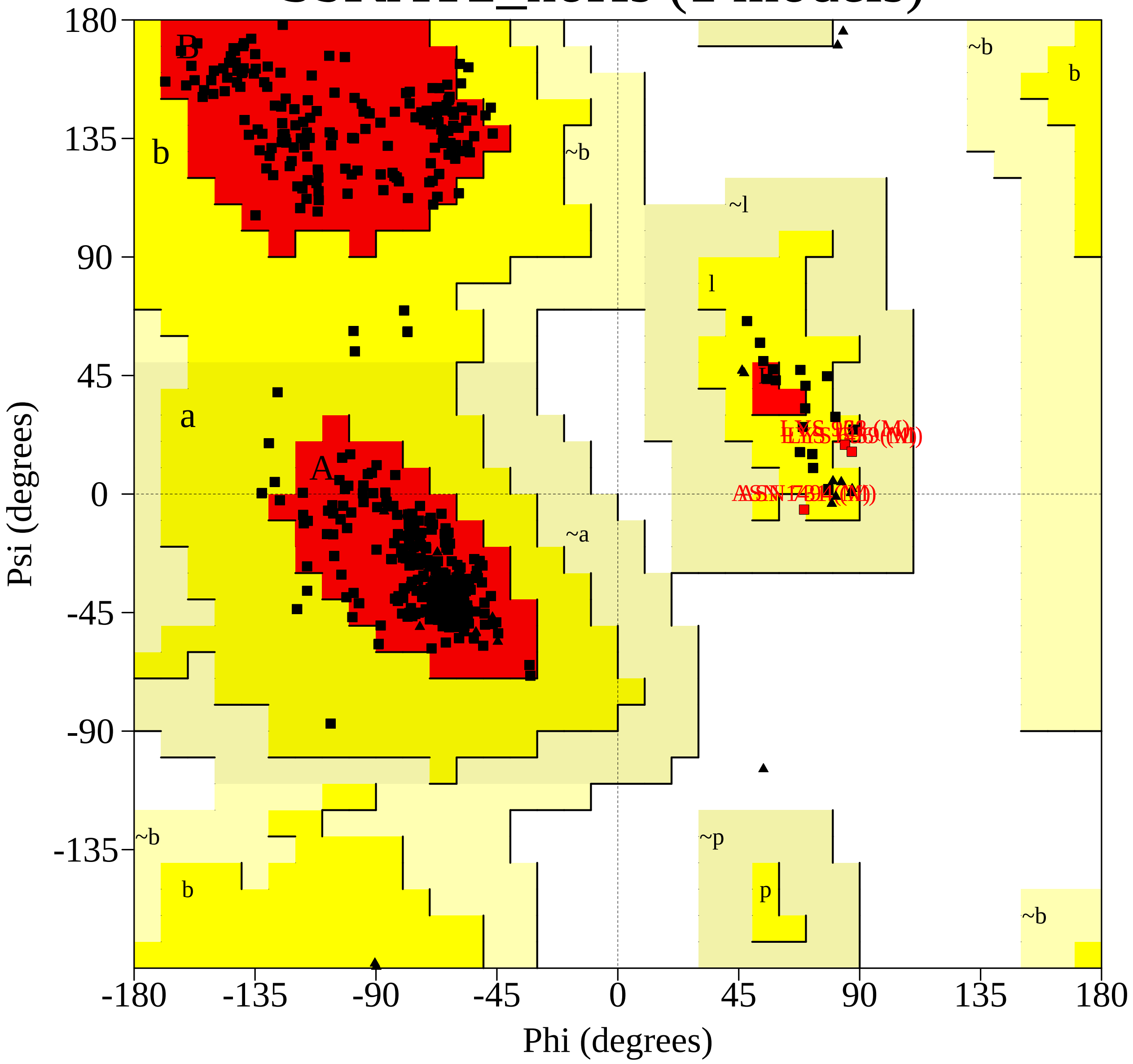

Plot statistics

Residues in most favoured regions [A,B,L]	1294	95.9%
Residues in additional allowed regions [a,b,l,p]	50	3.7%
Residues in generously allowed regions [~a,~b,~l,~p]	6	0.4%
Residues in disallowed regions	0	0.0%
	----	------
Number of non-glycine and non-proline residues	1350	100.0%
Number of end-residues (excl. Gly and Pro)	8	
Number of glycine residues (shown as triangles)	72	
Number of proline residues	56	

Total number of residues	1486	

Based on an analysis of 118 structures of resolution of at least 2.0 Angstroms and R-factor no greater than 20%, a good quality model would be expected to have over 90% in the most favoured regions.

PROCHECK-NMR

Ramachandran Plot

SbTPC1_noHs (1 models)

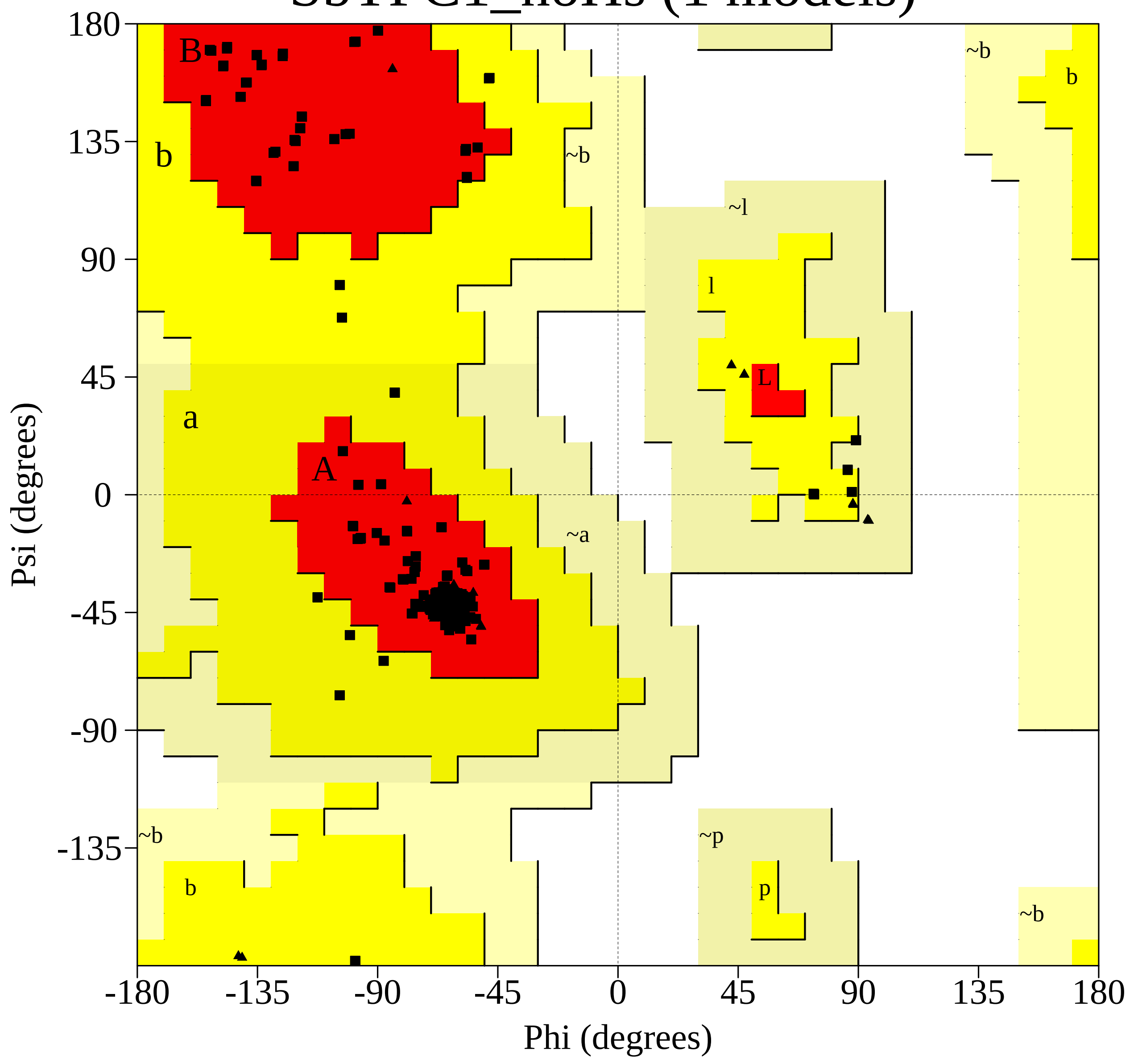

Plot statistics

Residues in most favoured regions [A,B,L]	360	93.3%
Residues in additional allowed regions [a,b,l,p]	26	6.7%
Residues in generously allowed regions [~a,~b,~l,~p]	0	0.0%
Residues in disallowed regions	0	0.0%
	----	------
Number of non-glycine and non-proline residues	386	100.0%
Number of end-residues (excl. Gly and Pro)	4	
Number of glycine residues (shown as triangles)	22	
Number of proline residues	2	

Total number of residues	414	

Based on an analysis of 118 structures of resolution of at least 2.0 Angstroms and R-factor no greater than 20%, a good quality model would be expected to have over 90% in the most favoured regions.

PROCHECK-NMR

Ramachandran Plot

SbTPC2_noHs (1 models)

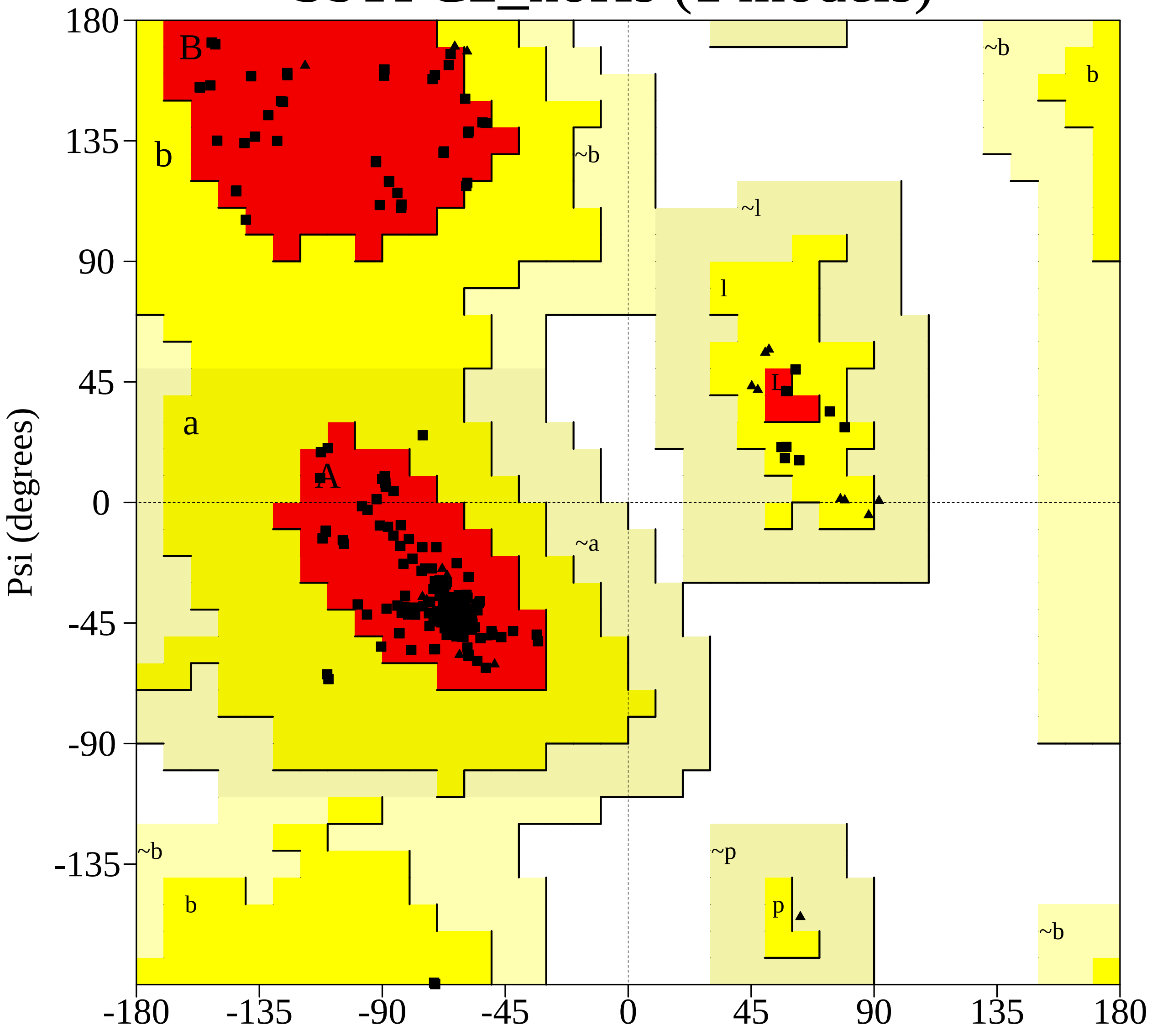

Plot statistics

Residues in most favoured regions [A,B,L]	303	95.3%
Residues in additional allowed regions [a,b,l,p]	15	4.7%
Residues in generously allowed regions [~a,~b,~l,~p]	0	0.0%
Residues in disallowed regions	0	0.0%
	----	------
Number of non-glycine and non-proline residues	318	100.0%
Number of end-residues (excl. Gly and Pro)	2	
Number of glycine residues (shown as triangles)	28	
Number of proline residues	10	

Total number of residues	358	

Based on an analysis of 118 structures of resolution of at least 2.0 Angstroms and R-factor no greater than 20%, a good quality model would be expected to have over 90% in the most favoured regions.

PROCHECK-NMR

Ramachandran Plot

SbKEA1_noHs (1 models)

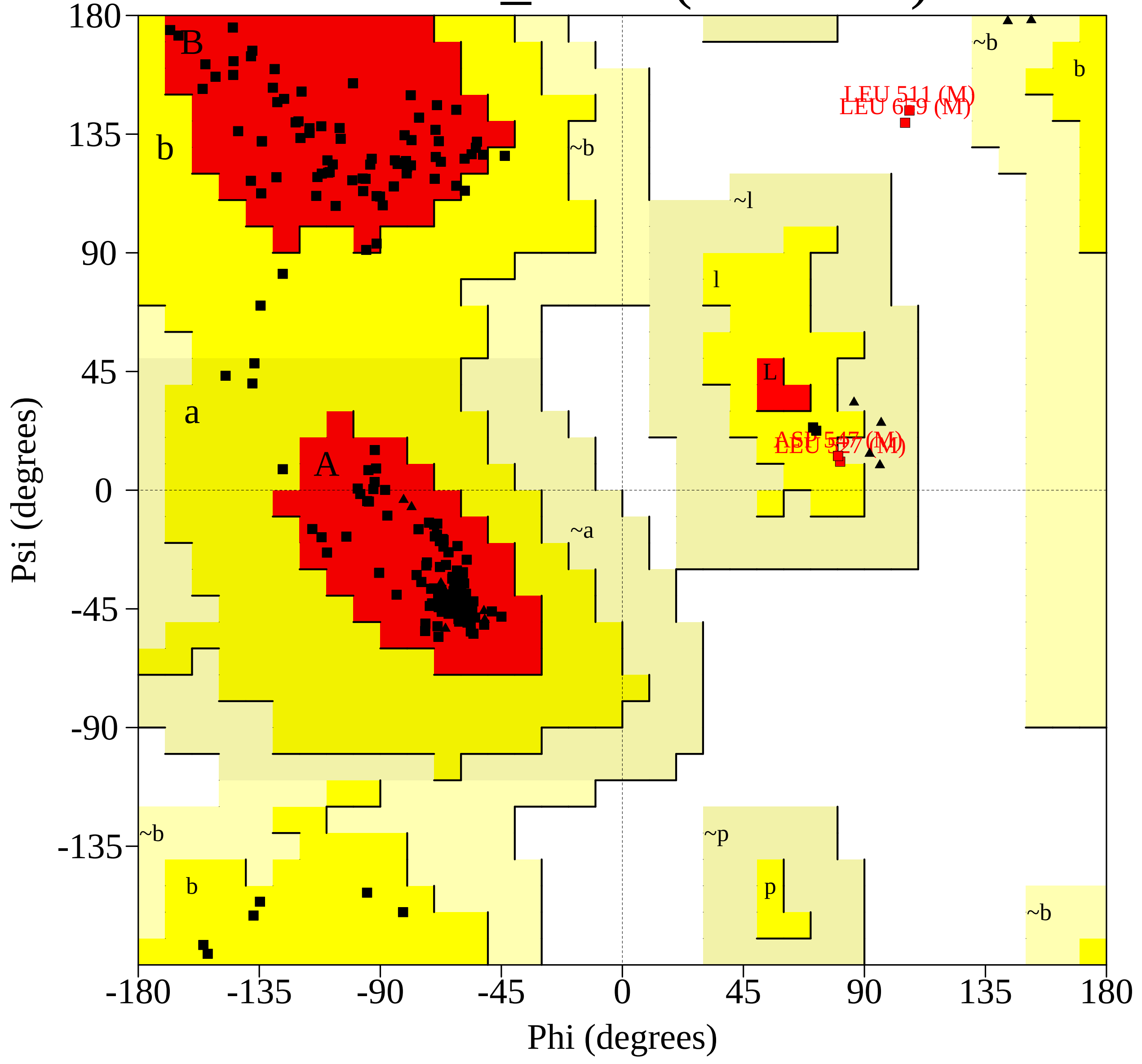

Plot statistics

Residues in most favoured regions [A,B,L]	228	92.7%
Residues in additional allowed regions [a,b,l,p]	14	5.7%
Residues in generously allowed regions [~a,~b,~l,~p]	2	0.8%
Residues in disallowed regions	2	0.8%
	----	------
Number of non-glycine and non-proline residues	246	100.0%
Number of end-residues (excl. Gly and Pro)	4	
Number of glycine residues (shown as triangles)	14	
Number of proline residues	12	

Total number of residues	276	

Based on an analysis of 118 structures of resolution of at least 2.0 Angstroms and R-factor no greater than 20%, a good quality model would be expected to have over 90% in the most favoured regions.

PROCHECK-NMR

Ramachandran Plot

SbKEA2_noHs (1 models)

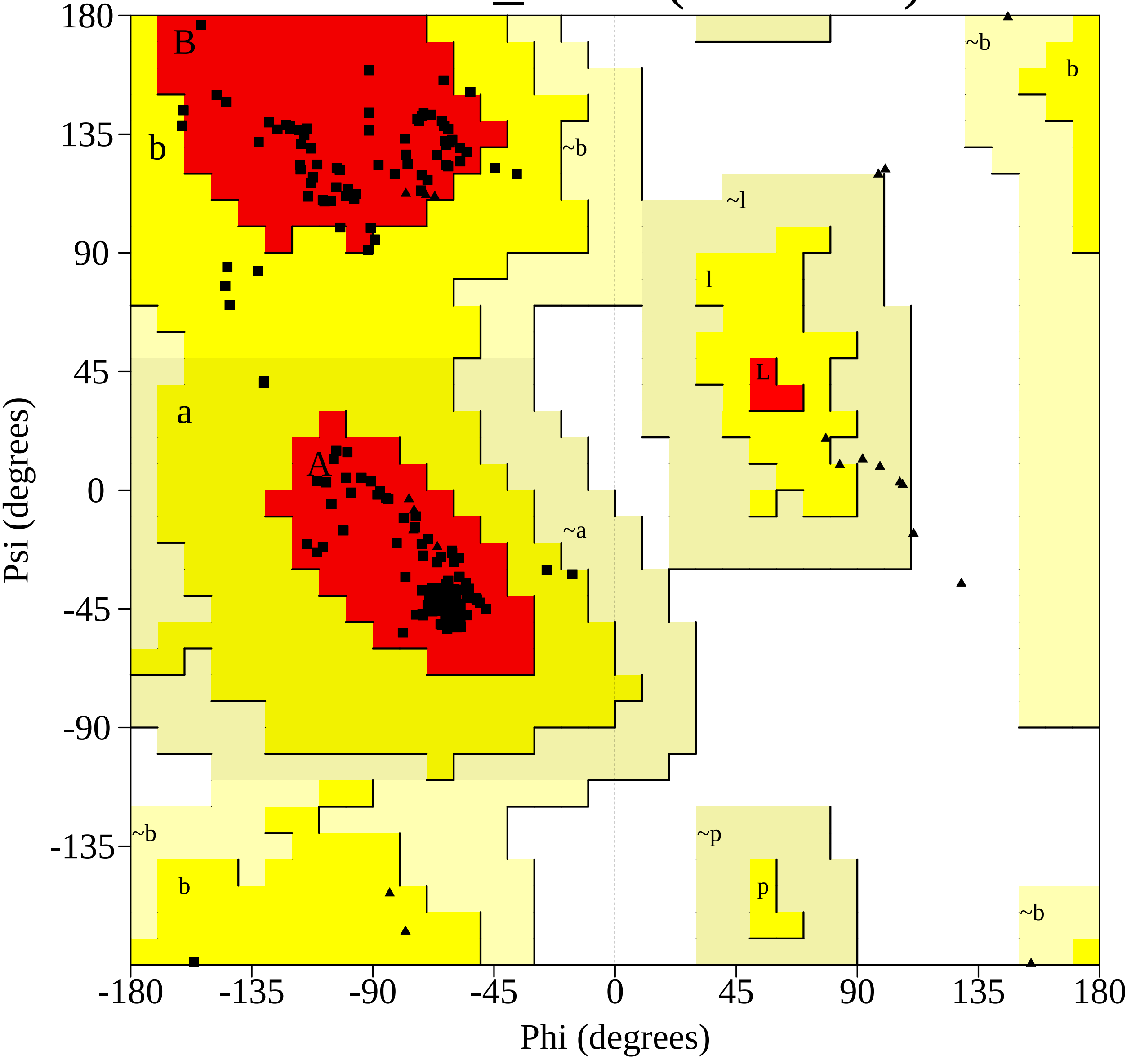

Plot statistics

Residues in most favoured regions [A,B,L]	225	93.8%
Residues in additional allowed regions [a,b,l,p]	15	6.2%
Residues in generously allowed regions [~a,~b,~l,~p]	0	0.0%
Residues in disallowed regions	0	0.0%
	----	------
Number of non-glycine and non-proline residues	240	100.0%
Number of end-residues (excl. Gly and Pro)	2	
Number of glycine residues (shown as triangles)	24	
Number of proline residues	8	

Total number of residues	274	

Based on an analysis of 118 structures of resolution of at least 2.0 Angstroms and R-factor no greater than 20%, a good quality model would be expected to have over 90% in the most favoured regions.

PROCHECK-NMR

Ramachandran Plot

SbVDPC1_noHs (1 models)

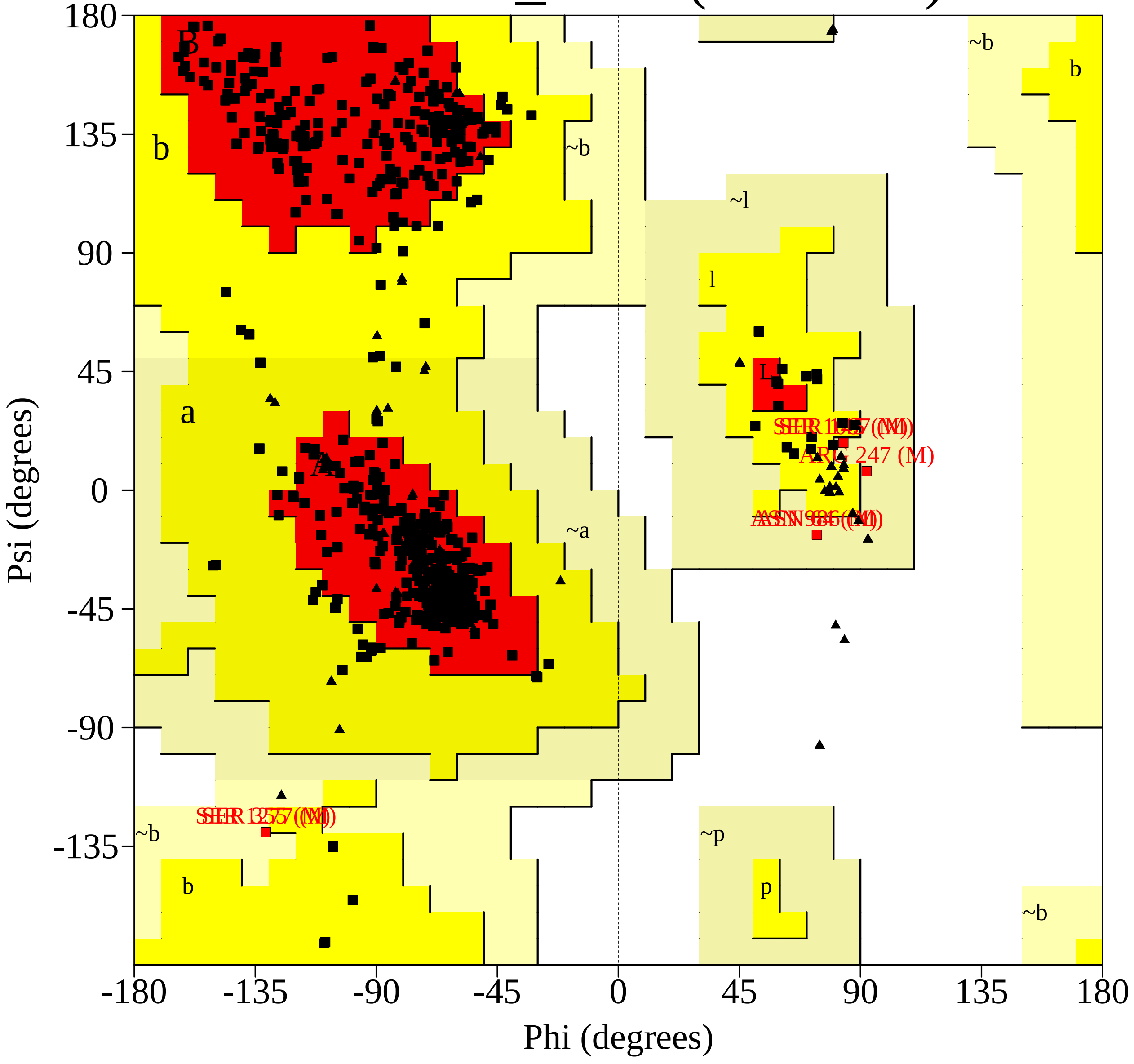

Plot statistics

Residues in most favoured regions [A,B,L]	1556	94.3%
Residues in additional allowed regions [a,b,l,p]	87	5.3%
Residues in generously allowed regions [~a,~b,~l,~p]	7	0.4%
Residues in disallowed regions	0	0.0%
	----	------
Number of non-glycine and non-proline residues	1650	100.0%
Number of end-residues (excl. Gly and Pro)	8	
Number of glycine residues (shown as triangles)	98	
Number of proline residues	48	

Total number of residues	1804	

Based on an analysis of 118 structures of resolution of at least 2.0 Angstroms and R-factor no greater than 20%, a good quality model would be expected to have over 90% in the most favoured regions.
